# A Computational Model for the Analysis of Lipoprotein Distributions in the Mouse: Translating FPLC Profiles to Lipoprotein Metabolism

**DOI:** 10.1371/journal.pcbi.1003579

**Published:** 2014-05-01

**Authors:** Fianne L. P. Sips, Christian A. Tiemann, Maaike H. Oosterveer, Albert K. Groen, Peter A. J. Hilbers, Natal A. W. van Riel

**Affiliations:** 1 Department of Biomedical Engineering, Eindhoven University of Technology, Eindhoven, The Netherlands; 2 Netherlands Consortium for Systems Biology, University of Amsterdam, Amsterdam, The Netherlands; 3 Department of Pediatrics, University Groningen, University Medical Center Groningen, Groningen, The Netherlands; 4 Department of Laboratory Medicine, University Groningen, University Medical Center Groningen, Groningen, The Netherlands; Ecole Polytechnique Fédérale de Lausanne, Switzerland

## Abstract

Disturbances of lipoprotein metabolism are recognized as indicators of cardiometabolic disease risk. Lipoprotein size and composition, measured in a lipoprotein profile, are considered to be disease risk markers. However, the measured profile is a collective result of complex metabolic interactions, which complicates the identification of changes in metabolism. In this study we aim to develop a method which quantitatively relates murine lipoprotein size, composition and concentration to the molecular mechanisms underlying lipoprotein metabolism. We introduce a computational framework which incorporates a novel kinetic model of murine lipoprotein metabolism. The model is applied to compute a distribution of plasma lipoproteins, which is then related to experimental lipoprotein profiles through the generation of an *in silico* lipoprotein profile. The model was first applied to profiles obtained from wild-type C57Bl/6J mice. The results provided insight into the interplay of lipoprotein production, remodelling and catabolism. Moreover, the concentration and metabolism of unmeasured lipoprotein components could be determined. The model was validated through the prediction of lipoprotein profiles of several transgenic mouse models commonly used in cardiovascular research. Finally, the framework was employed for longitudinal analysis of the profiles of C57Bl/6J mice following a pharmaceutical intervention with a liver X receptor (LXR) agonist. The multifaceted regulatory response to the administration of the compound is incompletely understood. The results explain the characteristic changes of the observed lipoprotein profile in terms of the underlying metabolic perturbation and resultant modifications of lipid fluxes in the body. The Murine Lipoprotein Profiler (MuLiP) presented here is thus a valuable tool to assess the metabolic origin of altered murine lipoprotein profiles and can be applied in preclinical research performed in mice for analysis of lipid fluxes and lipoprotein composition.

## Author Summary

Lipids such as triglycerides and cholesterol are important building blocks and energy suppliers for the body. However, because lipids are hydrophobic they require a matrix to circulate in the bloodstream. This function is fulfilled by lipoproteins. Altered lipoprotein metabolism is known to be associated with disease, for instance there is a well-known association between abnormal lipoprotein-mediated cholesterol transport and atherosclerosis. This change in lipoprotein metabolism is reflected in altered lipoprotein size and lipid composition as illustrated by a lipoprotein profile. We developed a mathematical approach, the Murine Lipoprotein Profiler, that links changes in lipoprotein profiles to alterations in lipoprotein metabolism. The model is developed to provide insight into the mechanisms underlying altered lipoprotein profiles in preclinical research in mice. The model can be used to gain further understanding of how a change in the lipoprotein phenotype can result from changes in lipoprotein metabolism.

## Introduction

The transport of lipids between mammalian tissues is largely facilitated by lipoproteins. Lipoproteins are spherically organized protein-lipid compounds that, unlike the individual lipids, are soluble in blood. To achieve this vital property the outer shell of the lipoprotein is a surface layer consisting mainly of phospholipids (PL), free cholesterol (FC) and proteins. The most hydrophobic lipids are stored in the core of the particle which consists largely of triglycerides (TG) and cholesteryl ester (CE).

The body produces three classes of lipoproteins [1], [2]. These classes differ with regard to size, lipid composition and protein content and have distinct origins [3], [4]. Chylomicrons, produced in the intestine from dietary lipids, contain mainly TG and represent the largest, least dense particles [5], [6]. The liver excretes TG-rich lipoproteins of a smaller size, which are known as very low density lipoproteins (VLDL) [4], [7]. High density lipoproteins (HDL), the smallest class, exhibit a relatively high protein and cholesterol content and originate in the intestine and liver [3], [8], [9]. Further processing of circulating chylomicrons, VLDL and HDL, in turn, generates additional classes of lipoproteins which vary in size and composition and exhibit great metabolic diversity [7], [10], [11], [12], [13].

Abnormalities of lipoprotein concentration, size and composition are known risk factors for cardiovascular diseases. More specifically, a low concentration of HDL cholesterol and/or a high concentration of low density lipoprotein (LDL) particles, as often observed in the Metabolic Syndrome and Type II Diabetes, is associated with an increased risk of atherosclerosis [2], [14], [15]. Preclinical research in mice is an important component in the search for treatment and prevention of these diseases [16], [17]. Despite the extensive use of mouse models the complex reactions determining plasma lipoprotein composition are not well understood. In this study we aim to develop a method which quantitatively relates murine lipoprotein size, composition and concentration to lipoprotein metabolism.

Lipoprotein size, composition and concentration can be quantified in a lipoprotein profile. In preclinical research, profiles are generated to analyse the impact of genetic, dietary and pharmacological interventions on the lipoprotein phenotype. These profiles are generally generated by means of fast protein liquid chromatography (FPLC), a method for size-based lipoprotein separation from serum or plasma [18], [19], [20]. The retrieved size fractions are subsequently analysed using biochemical assays to quantify lipid and protein contents. As lipoprotein size and composition are determined by multiple metabolic pathways, it is difficult to directly relate changes in lipoprotein profiles to the mechanisms that provoke these quantitative and qualitative differences.

The application of computational methods provides a powerful tool to retrieve insight into the complex relationship between lipoprotein metabolism and lipoprotein profiles. In recent years, two detailed computational models of human lipoprotein metabolism have been developed that relate lipoprotein distributions to underlying metabolism [21], [22]. Despite the importance of mouse models in preclinical research, there is no computational framework available to describe the emergence of lipoprotein distributions from biological principles in mice. Here we develop the Murine Lipoprotein Profiler (MuLiP), a computational strategy that quantitatively relates changes in lipoprotein characteristics to altered lipoprotein metabolism in mice. In the mathematical model, the distribution of the lipoprotein core composition is determined by kinetic models of VLDL, LDL and HDL metabolism. The model integrates these kinetic models with several sets of compositional information from literature [23], [24], [25], [26] and VLDL-TG production rates to provide a quantitative overview of the lipid fluxes between lipoproteins and their relationship to lipoprotein content. The model outcome is compared to experimental FPLC data by calculating an *in silico* FPLC profile from the model.

Due to the common generation of FPLC profiles in preclinical research of cardiometabolic diseases, MuLiP is widely applicable. The availability of lipoprotein production and composition data (e.g. in [24]) and FPLC profiles obtained from knock-out or transgenic mice is necessary for model application and validation, and therefore provides a further basis for the development and validation of the model. The MuLiPs comprehensive description of endogenous lipoprotein metabolism and compositional versatility allow a detailed description of the lipid content and associated metabolic state of plasma lipoproteins.

In this study, the model was first developed for wild-type C57Bl/6J mouse data. Following the successful description of the wild-type profile, model performance was evaluated by prediction of the profiles of several transgenic mouse models commonly used in preclinical cardiovascular research. Finally, the model was applied to analyse phenotype changes in profiles obtained from C57Bl/6J mice that were treated with the anti-atherosclerotic Liver X receptor (LXR) agonist T0901317 [27]. LXR has been identified as a central regulator of lipid metabolism [28], and its activation leads to complex and time-dependent regulatory actions which are incompletely understood.

## Methods

### Experimental data: FPLC profiles and VLDL-TG production

Two types of data were incorporated to develop the computational model: the experimentally determined VLDL-TG production, and the FPLC profile of plasma lipoproteins.

The FPLC profile was obtained from moderately fasted C57Bl/6J mice on a chow diet. The FPLC profile was collected as part of an extensive dataset, which was previously published in [27] and [29]. For the ethics statement and detailed experimental procedures, we refer to [29]. In short, the lipoprotein profile was determined by first separating lipoproteins through FPLC, followed by quantification of the TG and total cholesterol (TC) in each fraction.

VLDL-TG production was determined following administration of Triton WR-1339 (0.5 g/kg body weight).

FPLC profiles of three transgenic mouse models were retrieved from literature for model analysis and validation. The scavenger receptor class B type 1 (SR-B1) knock-out mouse [30] is a commonly used mouse model in cardiovascular research which has a plasma cholesterol concentration of between 50 and 200% higher than wild-type counterparts [30],[31],[32],[33],[34],[35],[36]. The HDL peak in the FPLC profile of SR-B1 knock-out mice is shifted to the left, indicative of larger sized particles [30].

Phospholipid transfer protein (PLTP) knock-out mice display decreased plasma cholesterol levels [37], [38] and smaller HDL particles containing less cholesterol. The HDL peak in the FPLC profile is shifted to the right in comparison to wild-type mice profiles [37].

While the deficiency of both previous mouse models is found in HDL metabolism, the low density lipoprotein receptor (LDLr) knock-out mouse ([39], [40], [41], [42], [43]) has a deficiency which mainly affects apolipoprotein (Apo) B containing lipoprotein metabolism. In chow-fed, wild-type mice, this deficiency leads to moderately increased plasma cholesterol concentrations, which are mostly due to increased cholesterol levels in the LDL size range. More moderate increases of VLDL and HDL cholesterol, as well as an increase of plasma triglycerides are also perceived. These profiles were reproduced *in silico* to qualitatively evaluate the results of simulation of a transgenic mouse model.

For computational analysis of the changes in murine lipoprotein metabolism in response to a pharmaceutical intervention, profiles of C57Bl/6J mice treated with LXR agonist T0901317 were analysed. FPLC profiles of treated mice, as well as the VLDL-TG production, were taken from the dataset described in [27] and [29]. The data set of treated mice consists of TC and TG FPLC profiles of C57Bl/6J mice following 1, 2, 4, 7, 14 and 21 days of treatment with T0901317. In addition to these profiles, measurements of the VLDL-TG production were performed in mice treated for 1, 7 or 14 days.

### Computational model

**Figure 1:**
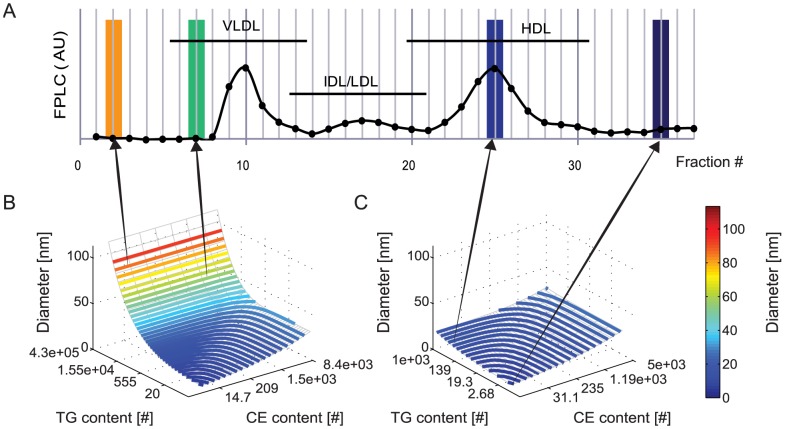
Relationship between modelled lipoprotein core composition and *in silico* lipoprotein profile. **A.** Representation of an FPLC profile. The profile was generated through calculation of the euclidean norm of the TG and TC content (in nmol) in each fraction of the untreated mouse FPLC profile, and is expressed in arbitrary units. **B.** Surface plot relating calculated VLDL diameter to CE and TG content. Contours represent the calculated positions of FPLC fraction boundaries. In addition to the depicted CE and TG contents the FC and PL contents are also determined for all lipoproteins. Figure 1 in Text S1 depicts the relationship between modelled lipoprotein core composition and surface components FC and PL. **C.** Surface plot relating calculated HDL diameter to CE and TG content. Contours represent FPLC fraction boundaries. A detailed description of the lipoprotein composition model is provided in Text S1. The calculation of the *in silico* lipoprotein profile is represented by the arrows, which connect FPLC fractions to the associated surface area in the model as bounded by the fraction boundary contours. All FPLC profiles are composed of discrete data points and are pictured as a series of dots, connected by a line which serves only to guide the eyes. For clarity, the highest measured fractions of the FPLC profile have not been pictured. Note that the highest pictured fraction, 37, corresponds to a diameter of 4 nm.

**Figure 2:**
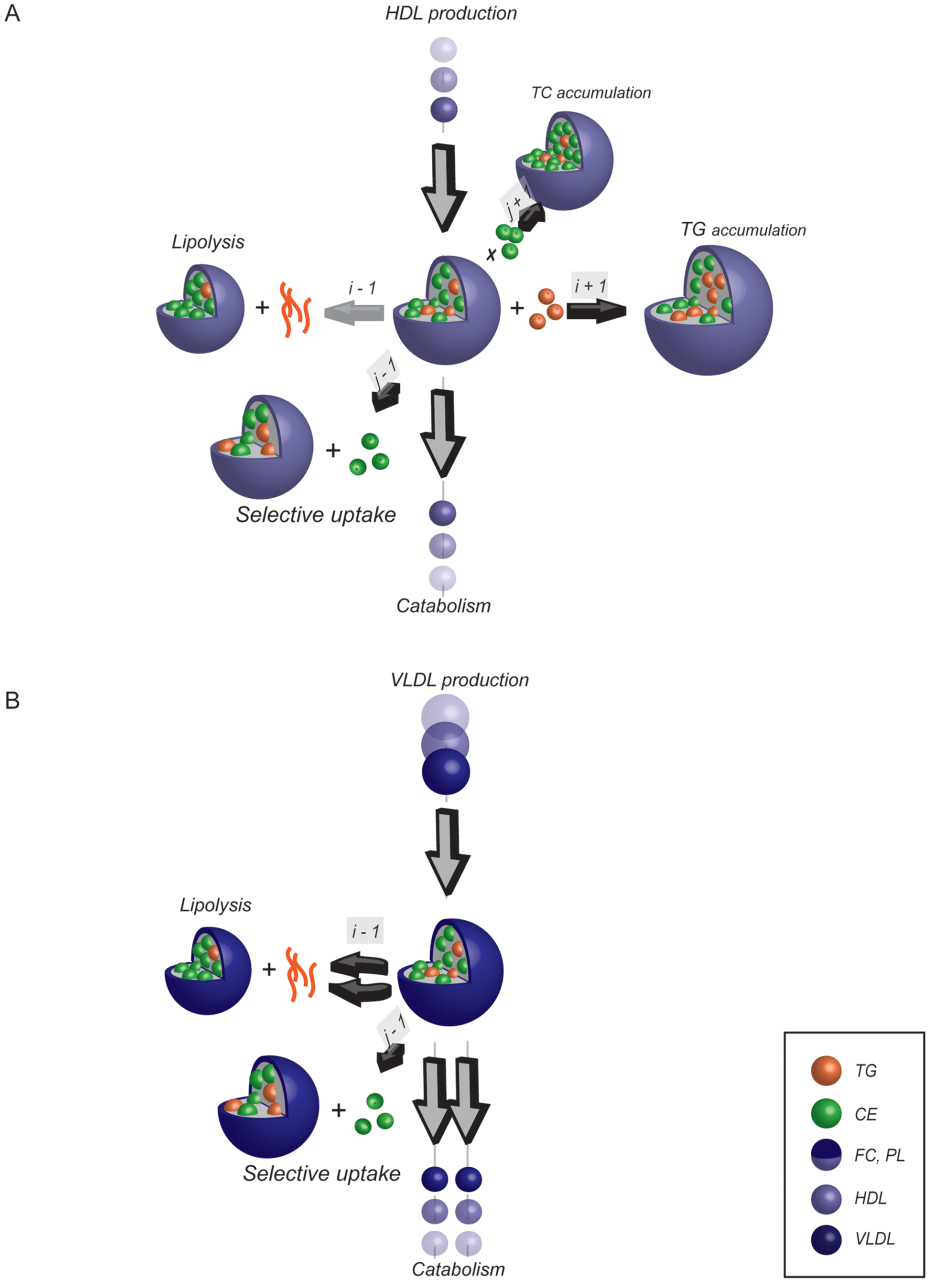
Lipoprotein metabolism model. **A.** Visualization of the HDL metabolism model. The central lipoprotein represents the lipoprotein with grid coordinates (*i*,*j*). The arrows represent the 6 processes that together determine the HDL distribution. HDL production and catabolism involve lipoprotein introduction to and removal from the grid and are pictured perpendicular to the plane of the grid. Within the lipoprotein grid, HDL may undergo lipolysis (resulting in a flux from (*i*, *j*) to (*i−1*, *j*)), obtain either CE or TG (resulting in a flux to (*i*, *j+1*) or (*i+1*, *j*) respectively) or undergo selective uptake of CE (resulting in a flux from (*i*, *j*) to (*i*, *j−1*)). A process in which the CE or TG content of the lipoprotein increases thus causes an increase of lipoprotein size and surface components, while a decrease of core lipid content is associated with a decrease in size and surface components (Figure 1 and Text S1). Each of the six fluxes is a function of lipoprotein composition and/or size. **B.** Visualization of the VLDL metabolism model. The VLDL sub-model structure is similar to the HDL sub-model structure, but the VLDL metabolism model differs from the HDL metabolism model. In the VLDL metabolism model lipoproteins may only decrease in size, and lipolysis and catabolism of VLDL are both the result of two equations. A full description of the lipoprotein metabolism model is given in Text S2.

In order to analyse the lipoprotein profiles, we developed a computational framework which will be described in the following three sections and is visualized in Figures 1 and 2 consecutively. The lipoprotein composition model (Figure 1 B and C), the models of lipoprotein metabolism (Figure 2 A and B) and the calculation of the *in silico* profile (Figure 1A) will be discussed.

#### Lipoprotein compositional model

In the computational model, we define a lipoprotein by three characteristics: type, CE content and TG content. Two types of lipoproteins are distinguished in the model: HDL and Apo B containing lipoproteins. The Apo B type, which will be further referred to as VLDL, comprises not only VLDL but also its derivative LDL. The content of the remaining major lipid constituents FC and PL as well as the size of the particle is then calculated from the lipoprotein core contents as described in this section. The type of lipoprotein and associated major Apo component are the basis for the metabolism model.

The lipoprotein diameter is calculated based on the volume of the two main core components TG and CE (as in [21], [44], [45], [46]), according to equation (1). This calculation of the lipoprotein sphere's diameter based on core volume uses two assumptions: (1) lipoprotein sphericity, and (2) a fixed surface layer thickness.
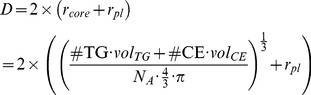
(1)


The calculation of lipoprotein diameter (*D*, in *nm*) is based on the number of molecules of core components (denoted by # TG and # CE respectively), the volumes of a TG and a CE molecule (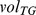 and 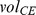, from [44], in 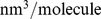), Avogadro's number (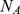) and the thickness of the surface layer (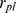, 2 nm, [44], [46], [47], [48], [49]). 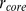 is the calculated radius of the lipoprotein core.

For calculation of the TC profile, we must define the FC content of the lipoprotein in addition to the CE content. The FC and PL content are calculated based on the experimentally determined ratio of surface lipids to core lipids for various lipoprotein sizes, as derived from published data (7 classes in [25], 1 class of nascent VLDL from [24]). The FC content is determined by linearly interpolating the ratio of # FC/(# CE+# TG) over the logarithm of the radius of the lipoprotein. Extrapolation, when necessary, is performed by taking the nearest ratios. An analogous calculation is performed to determine the amount of PLs, now using the ratio # PL/(# CE+# TG) (Text S1).

The absolute content of TG and CE molecules in a single lipoprotein spans a very wide range [21], [46], [50]. By defining the compositional model not in terms of absolute lipid content but in terms of derived indices *i* and *j* as will be described, the model is chosen to describe the metabolism and data more evenly. For the VLDL and HDL models, the same relation between the indices and lipid content is applied, but the model constants in this relation have values unique to the lipoprotein type. The values of the constants can be found in Text S2 and the diameters of the modelled HDL and VLDL are visualized in Figure 1C and Figure 1B respectively. In these figures, the diameter of the lipoproteins is plotted against the TG and CE content of the particle. In Text S1, the non-linear relationship between indices *i* and *j* and the TG and CE content is further demonstrated.

The TG content of a particle can be assumed to decline in an approximately exponential fashion when undergoing lipolysis [22], and therefore the expected distribution of lipoproteins over the triglyceride axis is not linear, but exponential. Using this assumption we define TG index *i* which relates to the triglyceride content via
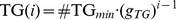
(2)Here 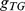 is the growth factor, which is fixed based on the maximal (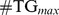) and minimal (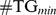) TG content via
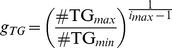




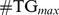
 is the TG content at 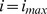. As stated previously, equation (2) is applicable to both the HDL and VLDL grids, but 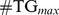, 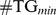 and 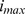 (and therefore also the values of 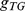 and 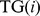) are defined separately for VLDL and HDL.

In HDL, the main murine cholesterol carrier, cholesterol metabolism is a dynamic process in which cholesterol is taken up by the particle and esterified, but also removed from the particle by a protein mediated selective lipid uptake process [3]. As accumulation of CE in the lipoprotein cannot be assumed to be proportional to its current CE content, an assumption that a change in a lipoproteins CE content is proportional to its current CE content is not appropriate. The equation that relates the CE index *j* to the CE content must differ from the relation introduced for TG. The CE index *j* is defined not on the basis of metabolism, but such that the distribution of lipoprotein diameter over *j* resembles the distribution of lipoprotein diameter over FPLC fractions. This strategy is employed to more evenly distribute the simulated lipoproteins over the data points in the FPLC profile.

We define the CE index *j* in equation (3), ensuring that for small HDL (i.e., HDL with minimal CE content), the *j* index is linearly related to the FPLC fraction number. In this equation, 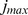 represents the maximal value of *j* (Text S2) and 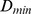 and 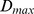 are the minimal and maximal lipoprotein diameters, which can be calculated from the minimal and maximal CE and TG contents. For a full derivation of this equation, we refer to Text S1.
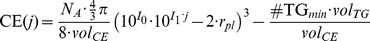
(3)with
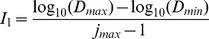
(4)and
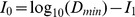
(5)


The parameters of the grid are defined for the physiological ranges of the lipoproteins. In VLDL composition the maximal TG content exceeds the maximal CE content and in HDL the maximal CE content exceeds the maximal TG content. TG index *i* therefore runs from 1 to 40 in VLDL and 1 to 8 in HDL whereas the CE index *j* runs from 1 to 8 in VLDL and from 1 to 40 in HDL.

#### Lipoprotein metabolism model

Lipoprotein metabolism as included in the model is biologically based [3], [4], [22], [24], [26], [27], [29], [45],[46], [50], [51], [52], [53], [54], [55], [56], [57], [58], [59], [60], [61] and is expressed as a series of ordinary differential equations describing lipoprotein production, remodelling and uptake. The lipoprotein metabolism models are visualized in Figure 2. For the full equations, description and rationale we refer to Text S2; the following section provides an overview of the kinetic models.

The series of differential equations in the HDL sub-model can be written as
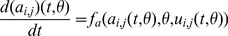
(6)Where 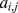 are HDL particles containing 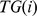 and 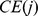, 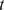 is time in hours, 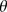 is a vector of 16 kinetic and physiological parameters, and 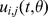 is the input of HDL with indices (*i*,*j*).

The concentration of particles is determined by production, remodelling, and catabolism. The positive term of the differential equation, the increase of lipoproteins with indices (*i*, *j*), consists of both an increase due to lipoprotein production and an increase due to remodelling of existing lipoproteins. Lipoprotein uptake and lipoprotein remodelling are a function of lipoprotein concentration and can be a function of lipoprotein composition and/or lipoprotein size.

Lipoprotein remodelling is always expressed as a change in one of the two main core components (Figure 2), and therefore a change in index *i* or index *j*. In order to prevent the lipoproteins exiting the model through remodelling instead of catabolism all model boundaries are closed. This is enforced by defining all fluxes over model boundaries as 0. As the boundaries have been defined much higher than the expected particle lipid content (Text S2), this does not influence the simulation.

The production of HDL in the framework is determined based on experimental data. The TC content of produced HDL is defined by *in vitro* data of nascent HDL [26]. The TG content of nascent HDL is chosen as the minimal TG content in the HDL model. Due to the exponential relation between 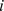 and 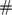 TG the model does not include particles without TG. The resulting production is written as:
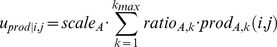
(7)Where 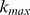 represents the number of different types of particles that may enter the system (here, *k* = 2). 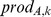 represents the distribution of the *k*-th type of HDL particle, and 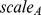 is a model parameter representing the amount of HDL particles produced (in 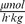). Finally, 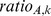 is a parameter that represents the relative contribution of the *k*-th type of particle to the HDL production. In order to preserve the definition of 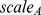 as the amount of particles entering the system, it must also hold that:
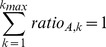
(8)In HDL remodelling four processes are defined (1) lipolysis of TG, (2) removal of CE from the particle (selective uptake), (3) uptake of peripheral FC by the lipoprotein and esterification of FC to CE (TC accumulation) and (4) uptake of TGs by the lipoprotein (TG accumulation). In general, the rate equations are phenomenological equations that describe the remodelling process based on lipoprotein size, composition and concentration. Remodelling steps in which lipids are removed from the lipoprotein are a function of the absolute amount of the targeted lipid in the complex. A correction factor is included in the rate equations to scale the fluxes to the rate of lipid content change (Text S2). This is necessary to correct for the non-linearity of the relation between indices *i* and *j* and lipid content and the variability of the grid dimensions in the model.

The general differential equation of the HDL sub-model (equation (6)) was extended to equation (9).
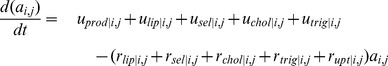
(9)Where lipoprotein production is denoted by the subscript *prod*, catabolism is represented by *upt* and the remodelling steps are indicated by *lip* (lipolysis), *sel* (selective uptake), *chol* (accumulation of TC) and *trig* (accumulation of TG).

HDL selective uptake is dependent on particle size and relative CE content. HDL lipolysis is a function of relative TG content. HDL TG and TC accumulation are a function of the rate of surface remnant entry into the system. HDL catabolism is inversely proportional to HDL size. Thus, HDL production is a function of 2 parameters (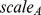 and 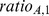, as 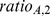 is fixed by equation (8)). HDL remodelling contains four equations, which are defined by a total of 5 parameters, and HDL catabolism is a single equation with a single parameter.

Analogous to equation 6, VLDL metabolism can be written as:
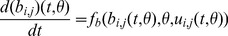
(10)


In the VLDL sub-model, the lipoprotein core content does not increase and equation 10 is extended to equation 11

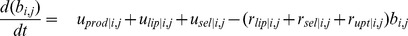
(11)


The production of VLDL in the framework is based on experimental data from [24], in which the ratio of TG and CE content in nascent VLDL is published (Text S2). In short, VLDL production is defined as a two-dimensional log-normal distribution over the TG and CE contents (in # of molecules). The nascent VLDL diameter is a model parameter which is used to calculate the value of the mean and variance of lipid content. The parameters of the log-normal distribution are then calculated based on this absolute mean and variance [24]. The rate at which lipoproteins are produced is finally scaled so that the simulated VLDL-TG production is equal to the measured value of the VLDL-TG. VLDL production is expressed by equation (12).
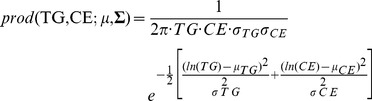
(12)with 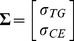 and 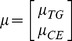.

VLDL metabolism contains only two remodelling processes: lipolysis and selective uptake. VLDL lipolysis and VLDL catabolism, however, are each described by two equations, and therefore the total VLDL metabolism model is composed of 6 equations. VLDL lipolysis comprises (1) the HDL lipolysis equation, which is presumed to be active on VLDL as well as HDL, and (2) a VLDL specific lipolysis equation, which is a function of VLDL size. The function of CE uptake from VLDL is chosen equivalent to HDL, but as the proteins involved are presumed different the parameter of the function is estimated separately for HDL and VLDL. VLDL catabolism is defined by (1) a constant catabolism term and (2) a surface area dependent catabolism function. VLDL production is defined by the single diameter parameter. VLDL lipolysis is described by four parameters. These are divided into the two parameters of the universal lipolysis function which have the same values as in the HDL sub-model and two parameters of the VLDL-specific lipolysis function. VLDL selective uptake is described by one parameter. The catabolism equations contain a total of four parameters. This brings the total model to 2×6 equations defined by 16 parameters, describing the concentration of 2×320 lipoprotein state variables.

#### Calculation of the *in silico* profile

FPLC fractions have been separated based on their diameter, according to equation (13) [62]. In order to transform the computed concentrations to a profile, we must therefore compare *in silico* size with FPLC fraction size range. FPLC fractionated size range is determined by calibrating the location of the VLDL, LDL and HDL peaks with their experimentally determined sizes in [23] via least squares.
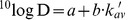
(13)


In this equation, 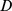 is the hydrodynamic diameter of the lipoprotein, 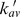 is the normalized value of the elution parameter 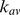 ([62]), and parameters 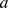 and 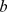 must be determined.

The calibration function is described by equation (13) with values of 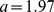 and 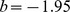. This yields the following sizes for (the median of) the three main lipoprotein classes: 41.5 nm (VLDL), 22.6 nm (LDL) and 11.3 nm (HDL) (Text S3).

To calculate the *in silico* lipoprotein profile, the diameter of the particles in the computational model is first calculated, according to equation (1). For each FPLC fraction, the size boundaries are calculated with the aid of equation (13). By combining both equations, a relationship between the (boundary) diameter 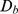 and indices *i* and *j* is determined. Finally, numerical integration between the fraction boundaries as expressed in *i* and *j* is performed to determine the number of simulated lipoproteins in each fraction. Multiplication by the TG or TC content of the lipoproteins results in *in silico* TG and TC profiles. The *in silico* profiles of PL, FC, CE, HDL and Apo B containing lipoproteins are generated through the same procedure. The calculation of the *in silico* profile is further visualized in Figure 1A and further explained in Text S3 ([63]).

#### Model simulation and analysis

Parameter estimation is performed using the FPLC profiles for control and LXR agonist treated mice [27] by simulating the computational framework as implemented in Matlab (7.10, The MathWorks, Natick, Massachusetts) and minimizing the cost function (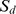) based on a sum of squared residuals. This is implemented using the non-linear least squares function *lsqnonlin*. The cost function is composed of a sum of squared residuals weighed with factors based on experimental data from literature ([23]), a penalty for a failure to reach steady state and a penalty for particles found in the unphysiological upper model boundaries (Text S4).

The VLDL and HDL sub-models were simulated consecutively. The rate of PL release from VLDL due to remodelling, which is an input in the HDL sub-model, was fixed to the steady state value calculated in the VLDL simulation. To estimate the 16 wild-type C57Bl/6J mouse parameters, the optimisation was initialized 1000 times with random 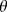 (Text S4). For the optimisation, the parameters are transformed to ensure a similar order of magnitude of all parameters. Geometrical parameters are transformed linearly, while kinetic parameters are transformed logarithmically. Details on parameter bounds and transformation are found in Text S4.

Model performance was assessed for each optimized parameter set by sampling the parameter space around the optimal parameter set and re-optimising. The calculation of a profile likelihood [64] and the generation of error plots of parameters of interest were used to further assess model performance and quality of the optimal parameter set.

## Results

### Computational analysis of lipoprotein metabolism in C57Bl/6J mice: From FPLC profiles to lipoprotein metabolism

**Figure 3:**
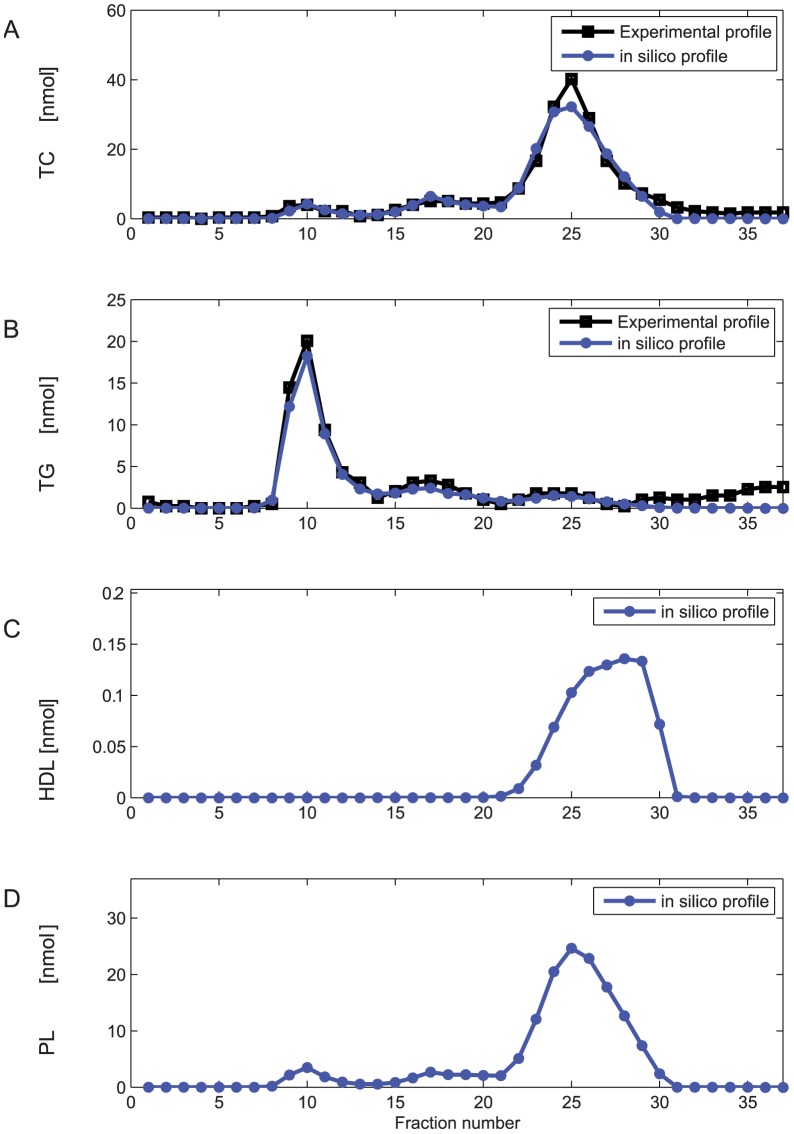
*In silico* fast protein liquid chromatography profiles of untreated C57Bl/6J mice. **A.** Cholesterol FPLC profile of untreated mice, experimental data and simulated profile. FPLC profile (black) of pooled plasma of moderately fasted, untreated C57Bl/6J mice and simulated FPLC profile (blue) total cholesterol content. The *in silico* profile was calculated with an optimized parameter set following model parametrisation (Text S4, parameter set X1). **B.** TG FPLC profile of untreated mice, experimental data and simulated profile. Experimental (black) and simulated (blue) TG profiles as in A. **C.** Computed profile of HDL. In addition to calculation of the measured profiles (A and B), calculation of profiles of all included model components is facilitated by the model. The parameters used to generate this profile are provided in Text S4. **D.** Computed PL profile, computed with the parameters provided in Text S4. For clarity, the highest measured fractions of the FPLC profile have not been pictured.

For model development, parameters were initially estimated based on control C57Bl/6J mouse lipoprotein profiles. Figure 3 A and B show the *in silico* TG and TC FPLC profiles with the experimental data for an optimized parameter set. The model describes the experimental data well in both the TG and the TC profile. We initiated 1000 optimization runs with randomly chosen initial parameter values. Two of the resulting optimized parameter sets described the data well in terms of number, location and height of the peaks. Moreover, these two optimized parameter sets displayed very reasonable values for the nascent VLDL diameter (Text S5). Figures 3 C–D demonstrate the added value of the model by providing estimates of particle number (Figure 3C, HDL) and unmeasured lipoprotein component (Figure 3D, PL) profiles.

We performed an analysis of the parameter space via re-sampling, profile likelihood analysis and calculation of the local error landscapes. The analysis revealed that the two parameter sets found represent the two local optima of the VLDL sub-model. While both optima result in a good fit of the FPLC profile and both are included in further analysis, the optimal parameter sets represent qualitatively different solutions. One of the solutions (to which X2 belongs) is the global optimum, while the other (to which X1 belongs) is a local optimum. The optima differ mainly in the size of nascent VLDL and the selective uptake parameter, as is illustrated in Text S5.

From analysis of the HDL sub-model parameter values, we concluded that the optimized ratio between five kinetic HDL parameters (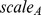, 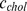, 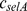, 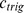 and 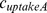) is consistent, but they are not identifiable individually. Thus one optimal set of correlated parameters and fluxes exists. This set can be straightforwardly scaled to desired, physiological values in the case that a single flux is known (Text S5). The sixth kinetic parameter, 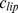, is found to not correlate with the remaining parameters. As this parameter is also present in VLDL sub-model, the value of 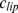 is instead constrained by the VLDL sub-model.

The value of HDL production parameter 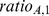 is found to be almost one (Text S5). This indicates that of the two distinct sizes of nascent HDL which were present in the experimental data on which the HDL production is modelled (Text S2), only the smaller particle is produced in the simulation. The wild-type model therefore simulates one type of newly produced HDL, which contains a mean of 24 cholesterol molecules. A comparison of parameter values with literature is found in Text S5 ([38], [46], [65], [66], [67]).

### Prediction of FPLC profiles of transgenic mouse models: From lipoprotein metabolism to FPLC profiles

For model validation we performed a series of simulations to generate lipoprotein profiles for three transgenic mouse models commonly used in cardiovascular research. The first two *in silico* transgenic mouse models have a deficiency in HDL metabolism, whereas the final modelled deficiency is in Apo B containing lipoprotein uptake.

**Figure 4:**
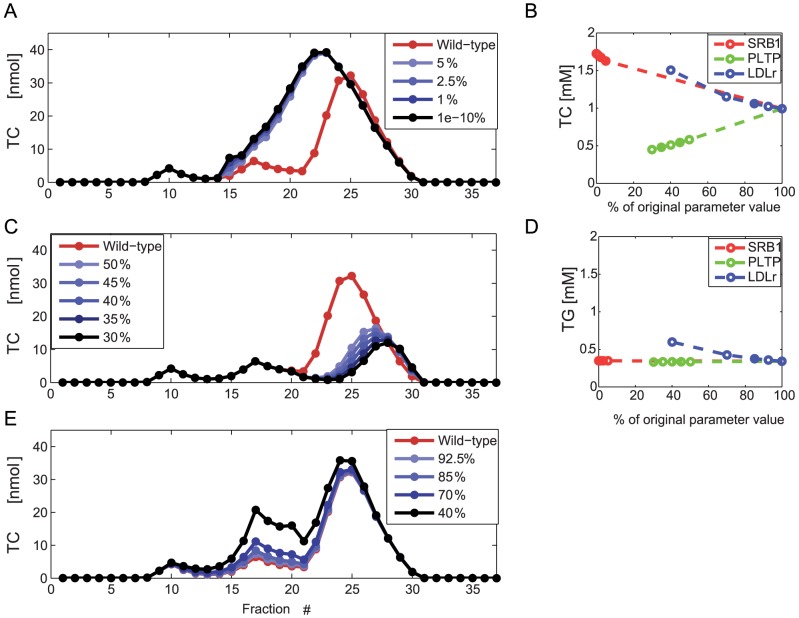
*In silico* cholesterol FPLC profiles of transgenic mice. **A.** Simulated cholesterol profile of the SR-B1 knock-out transgenic mouse. To simulate the SR-B1 knock-out mouse, the selective uptake parameter was set to between 10^−1^% (black) and 5% (blue) of the original value. For comparison, the untreated C57Bl/6J mouse cholesterol profile is drawn in red. For comparison, we refer to the FPLC profile of SR-B1 deficient mice in [30]. **B.** Change in plasma cholesterol concentration for the *in silico* transgenic mice as depicted in A, C and E. **C.** Simulated cholesterol profile of the PLTP knock-out transgenic mouse. To simulate the PLTP knock-out mouse, the parameter 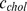 was diminished to values between 30% (black) and 50% (light blue) of its original value, increasing in steps of 5. The untreated C57Bl/6J mouse profile is again shown in red. Note that because the perturbed parameter in this case cannot be presumed to be solely dependent on PLTP activity, the parameter value was not reduced below 30%. For comparison, we refer to the FPLC profile of PLTP deficient mice in [37]. **D.** Change in plasma triglyceride concentration for the in silico transgenic mice as depicted in A, C and E. **E.** Simulated cholesterol profile of the LDLr knock-out mouse. To simulate the LDLr knock-out mouse, both VLDL sub-model whole-uptake parameters 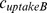 and 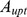 were diminished to a factor between 40% and 92.5% of their wild-type value. For comparison, we refer to the FPLC profile of LDLr deficient mice in [43]. The visualized *in silico* profiles have all been generated with parameter set X1. For clarity, the highest measured fractions of the FPLC profile have not been pictured. Further quantitative analysis of the results as well as the corresponding in silico profiles generated with X2 are presented in Text S5.

SR-B1 deficient mice exhibit impaired cholesterol exchange between HDL and tissues. To simulate SR-B1 deficiency the HDL selective uptake parameter 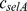 was decreased. To examine the results of the parameter perturbation for both a complete absence of the selective uptake mechanism as well as for a small amount of residual selective uptake, the simulation was performed for several values of the parameter 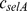. The results of the parameter perturbations are presented in Figure 4A. The predicted *in silico* SR-B1 knock-out lipoprotein profiles agree well qualitatively with published FPLC profiles of SR-B1 knock-out mice [30]. Consistent with phenotypes found in literature, the HDL particles of simulated SR-B1 knock-out mice are larger compared to wild-type mice. In the FPLC profile, this is visible as a shift to the left of the HDL peak. *In silico*, this is accompanied by a rise in HDL TC concentration of approximately 70% (Figure 4B).

In PLTP knock-out mice, PL transfer activity is markedly diminished as compared to wild-type mice [37], [38], [68], and HDL particles may be catabolised more rapidly than in wild-type mice [38], [68]. To simulate the PLTP knock-out mouse the HDL growth 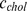 parameter was perturbed, representing a lower influx of surface remnants.


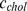
 is a parameter that represents a process in which not only PLTP, but also proteins such as Lecithin-cholesterol acyltransferase (LCAT) and ATP-binding cassette A1 (ABCA1) are involved. Furthermore, free cholesterol transfer activity may not be completely ablated in absence of PLTP [68]. Therefore, the knock-out phenotype is presumed to result from a perturbation in which 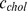 is in the range of 30 to 50% of the wild-type value. Choosing a value in this range for the simulation results in a reduction of plasma cholesterol corresponding to the 55% reduction of plasma cholesterol observed in the PLTP knock-out mouse [37]. The FPLC profile of the PLTP deficient mouse displays a slight shift to the right and the aforementioned reduction in plasma cholesterol [37]. The simulation results (Figure 4B) are in qualitative agreement with the results published in [37].

The third in silico transgenic mouse model simulated is the LDLr knock-out mouse ([39], [42], [43]). Experimental profiles of LDLr deficient mice show a marked increase in the LDL TC profile, a moderate increase in the VLDL and HDL size ranges and in most cases, an increase of plasma TG ([42], [39], [43]). The LDLr mediates both Apo E and Apo B dependent lipoprotein uptake. The in silico profile (Figure 4E) is produced by moderating both whole-particle uptake rate parameters 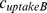 and 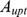 by the same factor. As mice produce around 70% of VLDL with the truncated Apo B48, around 30% is dependent on LDLr for uptake following conversion to LDL and the rates would be expected to decline by at least 30%. The resulting profiles (shown for different attenuation factors in Figure 4E) show qualitatively the same behaviour when compared to the wild-type profiles as seen in the experimental profiles [42]. The LDL cholesterol is moderately raised, and a lower increase is seen within VLDL and HDL size ranges. Plasma triglycerides show an increase that is lower (in an absolute sense) than the increase observed rise of plasma cholesterol (Figure 4D).

Further information on the simulation of knock-out phenotypes can be be found in Text S5.

### Computational analysis of lipoprotein metabolism in C57Bl/6J mice treated with an LXR agonist

Treatment with an LXR agonist affects many aspects of lipid and lipoprotein metabolism simultaneously [28], [69], [70]. The many targets of LXR include PLTP, SR-B1, Lipoprotein lipase, ApoE, ABCA1, ATP binding cassette G1 and other members of the ABC transporter family [27], [28], [71]. The modulation of lipoprotein metabolism is accompanied by changes in lipoprotein size and lipid content, including the presence of both enlarged VLDL [24], [58] and enlarged HDL [72], [71]. The enlargement of VLDL has been found to be associated with increased PLTP activity that results in the secretion of larger nascent VLDL, and the increase in plasma TGs is normally ablated by particle uptake [58]. The precise mechanism of HDL enlargement has not been fully elucidated, but has been associated with ApoE [71].

To simulate the lipoprotein profile of mice that have been treated with an LXR agonist several model constants were modified to comply with the experimental value (Text S6, [2], [3], [24], [51], [58], [59], [71], [73], [74]). The main modification encompasses the VLDL production, which was modified such that nascent VLDL in LXR agonist treated mice is of a larger size and higher TG content than nascent VLDL of wild-type C57Bl/6J mice. In Grefhorst *et al.* [24] the increase in VLDL-TG secretion was found to result mainly from an increase in TG content. In the simulation of T0901317 treated mice the nascent VLDL particles were therefore enlarged in diameter by retaining untreated particle secretion rate and nascent VLDL CE content while increasing the TG content. To achieve this, the TG content of nascent VLDL in treated mice was calculated by multiplying the untreated TG content with the relative increase in VLDL-TG production. The diameter of nascent VLDL was hereby fixed in relation to the diameter of VLDL of untreated mice, and the number of parameters in the model was reduced from 16 to 15.

Parameter optimization was performed by adding 50 random variations to the parameter sets obtained for untreated mice and re-optimizing as described for the untreated wild-type mouse (see Methods, Text S6). While many optimized parameter sets were successful in describing the general size of HDL, VLDL and LDL, the model was not able to reproduce the characteristic enlarged HDL, i.e. the second peak of the HDL which elutes in the LDL size range. From these simulations, we concluded that the model is unable to describe the mechanism responsible for the appearance of these particles and an extension of the model was necessary. We formulated three qualitatively different extensions to the model for untreated mice based on biological mechanisms that could be responsible for the appearance of large HDL particles.

The first model extension (E1) involves an extension in HDL remodelling, in particular the HDL TC accumulation equation. The cholesterol uptake equation is elaborated to include additional uptake for large lipoproteins. The equations represent a mechanism whereby Apo E may stimulate the uptake and esterification of cholesterol, e.g. by activation of LCAT activity [73].

The second extension (E2) that was tested modifies HDL catabolism by incorporating uptake of large, Apo E-containing HDL by Apo E-binding receptors, such as the the LDL receptor [73]. As such, it is represented by an equation (Text S6) similar to the equation (Text S2) designed to simulate Apo E-dependent uptake of VLDL.

The third extension (E3) expands HDL catabolism to include the additional appearance of larger than normal HDL particles [74]. This is simulated by including an input of very large, relatively TG-poor and CE-rich particles.

**Figure 5:**
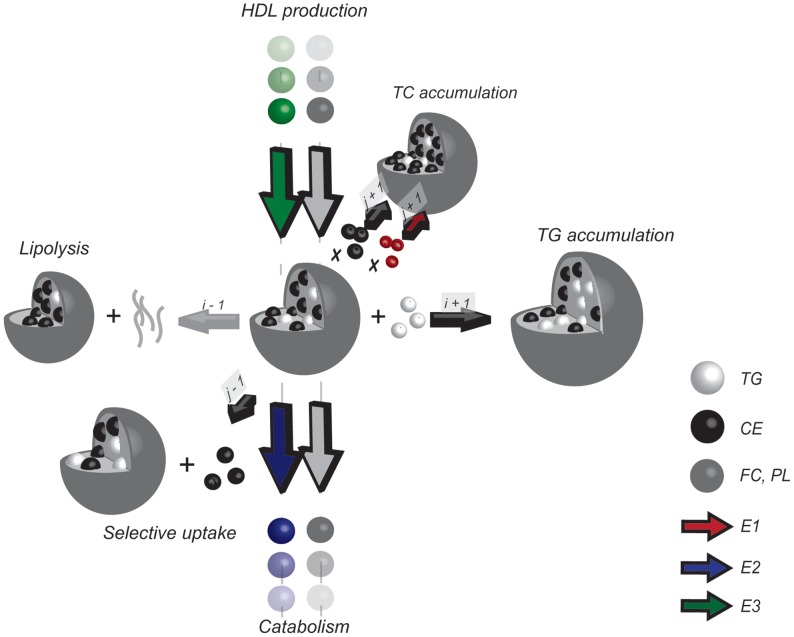
Overview of extended HDL metabolism. HDL metabolism, as depicted in Figure 2B, with extensions. For clarity, the original model of HDL metabolism is shown in grey-scale, while the three extensions are shown in red, blue and green respectively. The additional cholesterol accumulation of E1 is depicted in red. The additional lipoprotein uptake modelled in E2 is shown in blue. Finally the extension to include additional large nascent HDL as described by E3 is included via the green arrow. Note that while all extensions are shown here in the same figure, the three extensions are included in the model separately. More details on the model extensions are provided in Text S6.

The extended models comprise an addition of two or three to the 15 parameters in the original model. The extensions to the HDL model are visualized in Figure 5, and further details and equations are provided in Text S6.

Parameter estimation of the extended models was performed by optimizing the cost function as described in Text S4 for the profile obtained following 14 days of treatment. The parameters were initialized by initiating the 2, 3 or 3 parameters of the extended equation randomly within their bounds while initiating the 15 core model parameters at their wild-type value. The ability of a parameter set to simulate a profile was judged on both quantitative (i.e. TG and TC profile) and qualitative (i.e. appearance of the “enlarged HDL”) considerations. For a description of the VLDL in the profile on days one and two of treatment, it was necessary to estimate separately the previously described fixed VLDL size and TG content.

**Figure 6:**
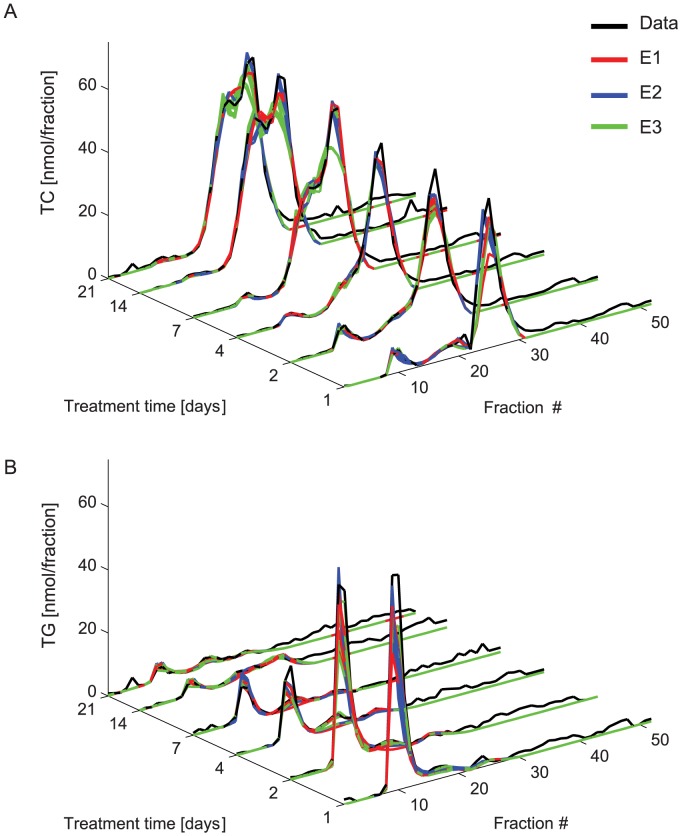
*In silico* fast protein liquid chromatography profiles for the C57Bl/6J mouse, treated with LXR agonist T0901317. Cholesterol (A) and triglyceride (B) profiles of the treated mouse at all time points. The 3-dimensional plot shows the *in silico* and experimental profiles as measured and simulated at six time points following initiation of treatment. For clarity, the time axis is scaled logarithmically. At each time point, the experimental profile is shown in black and *in silico* profiles generated with all accepted parameters sets of E1 (red), E2 (blue) and E3(green) are shown in colour. The vertical axis represents the fraction lipid content in nmol. The number of acceptable fits differs per time point and/or model extension, as optimized fits were evaluated for acceptability. Profiles from acceptable parameter sets are in many cases quite similar, and may not in all cases be distinguishable from each other. For clarity, the FPLC profiles have been pictured as lines; we note that both experimental and *in silico* profiles are in fact composed of discrete fraction measurements.

In Figure 6, the experimental and simulated profiles of the T0901317-treated mice are plotted for each time point following the initialization of treatment. Each of the three extended models is able to describe the enlarged HDL peak observed in the experimental data.

**Figure 7:**
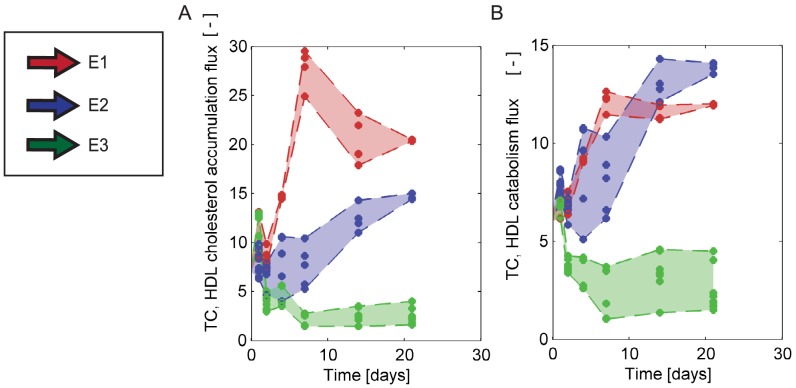
Quantitative distinction between hypotheses: fluxes. Following the acquisition of sets of acceptable parameters for all treatment durations, the fluxes of the (extended) HDL sub-model are plotted, as a function of time in days, as a ratio to the HDL TC production flux. Red = E1; Blue = E2, Green = E3. Fluxes are shown as lipid fluxes, of either cholesterol or triglycerides in various processes. **A** Cholesterol flux due to cholesterol uptake by the HDL particle, **B** Cholesterol flux due to HDL catabolism.

To identify differences between the three tested models, HDL lipid fluxes are plotted for all accepted parameters sets in Figure 7. As for the wild-type mouse, only the ratio between fluxes can be identified and therefore in Figure 7 the ratio of fluxes to the HDL TC production flux is depicted. Figure 7A shows the course of the peripheral cholesterol efflux to existing particles relative to cholesterol production. In Figure 7B, catabolism of HDL in terms of TC content is plotted. The fluxes calculated from the three extended models are distinguishable in their magnitude as well as their development over time (Figure 7A and B). Although the three different models yield equivalent lipoprotein profiles, there are clear differences in the predictions of lipid fluxes and lipoprotein metabolism. These differences can provide a basis for experimental differentiation between the mechanisms.

## Discussion

The well-established association between plasma lipoprotein concentrations and disease risk underscores the need to quantitatively analyse the factors determining lipoprotein distribution. In preclinical research, mouse models are often used to study the effect of a treatment on lipoprotein metabolism and disease risk. However for mice the computational tools available for human lipoprotein distributions ([21], [22]) are absent. In this study we have developed and applied MuLiP, which is a computational framework to analyse lipoprotein metabolism in mice. MuLiP can be used to expand understanding of lipoprotein metabolism based on commonly measured FPLC profiles. With this strategy, insight into lipoprotein concentration and composition, lipid and lipoprotein fluxes and lipoprotein remodelling processes can be gained. We showed that the model is able to analyse wild-type murine lipoprotein metabolism by deriving underlying metabolic processes from the lipoprotein profile. Furthermore, the model was shown to predict the profiles of transgenic mouse strains following a simulated perturbation of the underlying metabolism. Finally, the model was applied to provide novel insights into the metabolic adaptations underlying the response to a pharmacological intervention.

For many years, the interpretation of tracer experiments in humans has been aided by compartmental models [2], which have yielded valuable insights into lipoprotein life cycles. The application of these models to describe long-term effects on lipoprotein size and composition has limitations because the composition and size of lipoprotein classes are generally predefined in such methods [21], [22], [75]. Moreover, these models often focus on a single lipoprotein class (e.g. HDL: [76], [77], [78], [79]; or VLDL: [80], [81], [82], [83], [84], [85], [86], [87], [88], [89], [90], [91], [92]). In addition to these tracer dynamics models several mathematical models have been published which describe the dynamics of isolated lipids, proteins or lipoproteins ([93], [94], [95], [96], [97]), but cannot provide an integrated view of the relationship between lipid fluxes and lipoprotein distributions.

Two models of human lipoprotein metabolism have been proposed that assume a semi-continuous distribution of lipoprotein components [21], [22]. These two models have been shown to retrieve valuable insight into lipoprotein metabolism [21] and disease risk [22], [98], [99] that can be extracted by quantitatively evaluating human lipoprotein distributions. The Particle Profiler developed by van Schalkwijk *et al.* [22], [98], [99] describes Apo B containing lipoprotein metabolism and assumes that Apo B containing lipoprotein metabolism and composition rely solely on lipoprotein diameter. The computational model developed by Hübner *et al.* [21] describes the detailed metabolism of HDL and Apo B containing lipoprotein particles. Lipoprotein particles are represented by a large number of possible configurations of TG, TC and protein content.

Previous models are inapplicable to murine lipoprotein profiles for several reasons. First of all, to create a complete FPLC profile VLDL, LDL and HDL should all be described by the model. However, the majority of existing models focus on a single major lipoprotein class. Additionally, the models include assumptions of lipoprotein composition which are not suitable to describe the composition of murine lipoproteins. Finally, several differences between human and mouse (fasting) lipoprotein metabolism preclude human lipoprotein metabolism models from application to murine physiology. For instance, in mice HDL contains the majority of cholesterol present in plasma. In humans LDL is the primary cholesterol carrier [100]. Also, several differences in protein presence and localization must be considered in the development of the metabolic model. An important lipid exchanger in HDL and LDL metabolism in humans, Cholesterylester transfer protein (CETP), is absent in wild-type mice [100]. Furthermore hepatic lipase is found mainly in the liver in humans, but is present free in plasma in mice [100]. The non-specificity of Apo B 48 to chylomicrons in mice can be considered a final complicating factor.

In contrast to the aforementioned models, our novel computational model was designed specifically for the analysis of profiles obtained from mice. This required a novel model of murine lipoprotein metabolism. Future applications of MuLiP will require the integration of new data, extension of the model and therefore re-optimization of model parameters. Therefore, computational complexity and speed were important design criteria.

A coarse-grained non-linear description of the lipoprotein composition space was applied to reduce computation time and distribute the data evenly over the model. Both the relation between fraction number and lipoprotein size and the relation between lipoprotein diameter and lipid content are non linear. In the case of a linear grid, these relations will result in an imbalance in the large amount of states corresponding to a single fraction for fractions on the left-hand side of the profile, which correspond to a relatively large diameter range. The flexibility of the lipoprotein composition is ensured through the inclusion of both CE and TG as variables and inclusion of multiple remodelling processes. The metabolic model has been designed to focus on lipoproteins of which the FPLC profile contains most detail, e.g. spherical HDL as opposed to discoïdal HDL. By including VLDL, LDL and HDL, different aspects of lipoprotein metabolism can be examined simultaneously. The model is capable of providing detailed information on the metabolism of a certain (sub)class of lipoproteins. MuLiP is also capable of describing the large compositional variation observed in murine lipoproteins during genotypical or pharmaceutical interventions, such as the ones described in this study.

The framework was developed for analysis of lipoprotein metabolism based on two distinct data types: FPLC profiles (TC and TG) and VLDL-TG production flux. This combination of data provides the types of information necessary for the application of a detailed computational model of lipoprotein metabolism. The FPLC profile contains information on lipoprotein concentration, size and lipid content. The VLDL-TG production provides a VLDL sub-model flux which allows absolute determination of lipoprotein kinetics [98]. In this study, VLDL-TG production is estimated following the injection of Triton WR-1339 into both wild-type mice and mice treated with T0901317. In the model, this measure of VLDL-TG production is directly used to determine VLDL production. A possible alternative implementation of the measured rise in VLDL-TG production following Triton WR-1339 injection is the calibration of the model to the rise in plasma TG during 4 hours while *in silico* peripheral lipolysis is “turned off.” In Text S5, this alternative implementation is demonstrated. The predicted difference between total VLDL-TG production and the Triton WR-1339 estimate of VLDL-TG production *in silico* is found to be relatively small. This is in accordance with literature data that quantifies the reduction of the VLDL fractional catabolic rate following injection of a lipolysis inhibitor at 90% [67]. In the current study, a measurement of HDL sub-model fluxes following the pharmaceutical intervention is absent. While it is not within the scope of the current study, several strategies to determine these fluxes are available. Two computational models are available that could provide lumped HDL lipid fluxes in the mouse (Tiemann *et al.*: [70] and [29] and van de Pas *et al.*: [101]). We note that in [29], the computational model is applied to the same pharmaceutical intervention analysed in this study. A similar approach to scale the HDL sub-model with an absolute flux is described for the wild-type mouse in Text S5.

FPLC is a technique used to separate lipoproteins based on size [62]. However, the relationship between particle size and fraction number is non-trivial. In many cases the particle sizes in FPLC profiles found in literature are unquantified. In our computational framework, a calibration based on a quantified, untreated mouse profile [23] is applied to determine lipoprotein size.

A second challenge of FPLC profiles reported in the scientific literature is that most studies only report a single measurement of an FPLC profile. FPLC profiles are highly reproducible [25], however the absence of information on data uncertainty precludes the application of many statistical methods. Weighting functions and model acceptance have therefore been developed based on (1) observed variations in lipid content in literature data [23] and (2) analysis of the variability in the dataset applied in the current study (Text S4).

Murine lipoprotein metabolism in MuLiP is described by the interconnected VLDL and HDL sub-models. The VLDL sub-model describes VLDL and LDL content, composition and metabolism. We note that the lipoproteins in the VLDL sub-model can be computationally separated into VLDL and LDL metabolism. The low concentrations of LDL in mice complicate the analysis of LDL metabolism via an FPLC profile. However, by coupling VLDL and LDL metabolism we increase the information available on LDL metabolism and the associated risk of cardiovascular disease. The HDL sub-model describes the metabolism of spherical HDL. Both metabolic sub-models take into account lipoprotein heterogeneity as they are functions of lipoprotein composition and size. The composition of lipoproteins in the model is based on the TG and CE content of the particles. The remaining lipid constituents of the lipoproteins are calculated from the TG and CE content of the lipoprotein. The interpolation functions for this calculation are obtained from experimental data [25]. Computational model analysis revealed the existence of two qualitatively and kinetically distinct local optima of the metabolic model, which describe the data well (Text S5). The model was further analysed by the simulation of several transgenic phenotypes.

The SR-B1 and PLTP knock-out phenotypes have been successfully reproduced by perturbing a single parameter in the HDL sub-model. These transgenic models have been studied extensively in cardiovascular research (SR-B1: [30], [31], [32], [33], [35], [36], [34], PLTP: [37], [72]) and the predictions serve as a validation of model performance. SR-B1 is the main protein responsible for HDL cholesterol selective uptake and both heterozygous and homozygous SR-B1 knock-out mice display increased levels of plasma and HDL cholesterol [30]. In homozygous SR-B1 knock-out mice, a shift of the HDL peak to the left in the FPLC profile is observed [30], indicative of increased HDL size. The *in silico* SR-B1 knock-out profiles (Figure 4) predict the same behaviour as seen in the experimental models; the plasma cholesterol concentration is increased by approximately 70% (Text S5). This increase is slightly lower than the typical value of 100% ([35], [36]) and falls within the range of HDL cholesterol increases observed in SR-B1 knock-out mice experimentally ([30], [31], [32], [33], [34]). The shift to the left, indicating the presence of larger HDL, can clearly be observed in the *in silico* profile (Figure 4A).

Mice deficient for PLTP show clear reductions in all major components of HDL, including reduced HDL C, Apo A1 and PL levels [37]. This reduction of HDL cholesterol in PLTP deficient mice has been attributed to reduced transfer of phospholipids from triglyceride-rich lipoproteins to HDL [37] combined with increased catabolism of HDL [38]. Such HDL “hypercatabolism” is thought to result from changes in HDL composition in PLTP knock-out mice [38]. In the model, PLTP deficient mice have been simulated by decreasing the PL flux to HDL by decreasing the combined lipid accumulating parameter 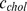. In Figure 4B, this was shown to generate an *in silico* FPLC profile which predicts a decrease of HDL cholesterol similar to that seen in literature.

The in silico PLTP and SR-B1 deficient mice demonstrate the validity of the HDL sub-model cholesterol metabolism. However, in preclinical research, mouse models deficient in Apo B containing lipoprotein metabolism are often used due to their higher, more human-like LDL cholesterol concentration and propensity to develop atherosclerosis. The LDLr and Apo E knock-out models, for example, are two of the models most often used in cardiovascular research [40]. Therefore these models are of interest for the validation of the model. Simulation of an Apo E knock-out mouse, however, may require extension of the MuLiP model. First of all, the clearance of chylomicron remnants requires Apolipoprotein E [5], [40] and therefore, absence of Apo E results in accumulation of chylomicron remnants. As the computational model currently does not incorporate chylomicron metabolism, it is unsuited to simulate chylomicron remnant accumulation. Secondly, Apo E is known to have several functions in not only chylomicron, but also VLDL and HDL metabolism. Apo E may mediate the uptake of both VLDL and chylomicrons and regulate LPL activity, and VLDL production is changed in Apo E knock-out mice [40], [67]. Because the MuLiP model is based on processes rather than genes or proteins, an *in silico* Apo E knock-out mouse is possible only if data is available that will guide the necessary parameter changes.

The LDL receptor is a related protein, however is not necessary for chylomicron clearance and LDLr knock-out mice do not show modulation of VLDL production [67]. The qualitative evaluation of an *in silico* LDLr knock-out mouse requires the perturbation of at least two parameters. By scaling both parameters with the same factor the LDLr knock-out mouse is simulated in the most simple way, however this results in qualitative agreement between the experimental and *in silico* profiles (Figure 4 E). The *in silico* LDLr deficiency reproduces the main characteristics of LDLr knock-out FPLC profiles: moderately raised LDL cholesterol, mild increases in cholesterol in the VLDL and HDL size ranges and an increase in plasma triglyceride which is seen *in vivo* ([41], [42], [43]).

Application of the original model, developed with wild-type mouse data to profiles of mice treated with pharmaceutical agent T0901317 did not retrieve an accurate description of the data. VLDL production was modified in accordance with experimental data of VLDL production following treatment with an LXR agonist [27], [29]. The model was applied to profiles of mice treated with an LXR agonist for 14 days first without re-optimizing parameters. The 15 model parameters were then re-optimized. The model was not able to adequately reproduce the data in either case (Text S6). In particular, the enlarged HDL typically observed upon LXR activation ([72]) could not be described. We concluded that the model was missing (a) mechanism(s) involved in the generation of the enlarged HDL and applied general extensions to the production (E3), remodelling (E1) and catabolism models of HDL (E2).

From the results generated by the three extended models of LXR-activation we conclude that all three models were equally capable of simulating the appearance of larger HDL. Biologically, the interpretation of each of the three extensions is distinct. E1 hypothesizes that an additional influx of cholesterol takes place in particles of a certain size. We base this hypothesis on the possibility of a mechanism that increases cholesterol accumulation of large-Apo E-rich particles. Apo E is known to interact with a variety of lipid metabolism related proteins [73]. In E2, we hypothesize that the Apo E present on HDL may interact with lipoprotein receptors [51], [73]. This results in increased catabolism of Apo E containing particles. In E3 the appearance of the second peak is caused by the sudden appearance of large particles. Kurano *et al.* [74] found that hepatocytes, when stimulated with an LXR-agonist *in vitro*, can produce larger, Apo E-rich HDL. However, as with the previous two hypotheses, the implemented equation is of a general nature and allows multiple biological interpretations. The third extension, e.g., may represent a mechanism that converts HDL to particles of a larger size. It is also conceivable that a combination of mechanisms is responsible for the observed size shift.

The model results incorporate not only the parameter perturbations. The results of the LXR agonist treated models e.g. quantify the differences in the relationship between total HDL production, remodelling and catabolism for the presented mechanisms, and can also be used to create flux FPLC profiles, which depict the simulated distribution of these processes over the FPLC fractions. The differences between models can be quantified for each of the lipids or processes included in the framework. The results also quantify of which HDL particles (i.e. which diameter, which composition) the catabolism or remodelling has been changed. Finally, the extended models can be used to predict the response of an LXR agonist treated mouse when a second intervention or genetic perturbation is applied. In Text S8, for instance, simulations of SR-B1 and PLTP knock-out mice treated with T0901317 were generated. Comparison of the predicted profiles with experimental results can give further insight into the biological relevance of the proposed mechanisms.

In the future, MuLiP could be used as an additional analysis tool for FPLC profiles. While processes are currently incorporated into the model in a phenomenological manner, isolating the effect of a gene or protein on lipoprotein metabolism will require the inclusion of more (flux) data, model expansion and parameter (re-)optimization. In cases where the VLDL production differs substantially from the assumptions made in this study, the implementation of a case-specific VLDL production distribution may be necessary. We note that with data of nascent VLDL composition and particle size distribution a novel VLDL production type can be derived and implemented without further model adaptation.

Another genetic modification commonly used in mouse models is the addition of CETP [102]. This neutral lipid exchanger links HDL and VLDL metabolism in humans, but is normally absent in mice. To allow application of the framework to mouse models that express CETP, the lipoprotein profile of which more closely resembles human lipoprotein profiles, it will be necessary to incorporate CETP in the model as a further link between HDL and VLDL metabolism. Depending on the data available and desired application, various implementations differing in complexity could be included. The most simple implementation of such a neutral lipid exchanger could entail a pair of coupled additional remodelling functions, i.e. a VLDL remodelling function that decreases TG content and increases CE content, quantitatively coupled to an HDL function that decreases HDL CE content and in turn increases HDL TG content. A more sophisticated equation could entail the definition of an explicit CETP protein moiety (as in [21]), the composition of which would determine the orientation of both the remodelling functions.

In conclusion, we developed a computational strategy to analyse FPLC profiles based on mouse lipoprotein metabolism and composition. The model predicts lipoprotein production, remodelling and catabolism fluxes which collectively determine changes in lipoprotein profiles. Following initial application of the model to FPLC profiles of the C57Bl/6J mouse, it was used to successfully predict phenotypes of transgenic mouse models commonly used in cardiovascular research. Furthermore, the model was applied to analyse and elucidate the complex phenotypes resulting from a pharmacological activation of the nuclear receptor LXR. MuLiP can contribute to insight into changes in lipoprotein metabolism underlying observed changes of a lipoprotein profile.

## Supporting Information

Text S1 **Additional calculations.** Extended calculation of CE index *j*, calculation of the free cholesterol content and phospholipid content and overview of the compositional model.
(PDF)

Text S2 **Wild-type model equations.** Derivation and equations of the wild-type HDL and VLDL sub-models.
(PDF)

Text S3 **FPLC profile calculation.** Steps 2 and 3 of the calculation of the FPLC profile. Both the calculation of the concentration FPLC profiles and the calculation of the flux FPLC profiles is explained.
(PDF)

Text S4 **Wild-type model parametrisation.** In this text the cost function, the parameter transformation, model simulation and the analysis of the results are discussed. Also, the resulting parameter values are reported.
(PDF)

Text S5 **Wild-type model analysis.** This text describes the performed re-sampling of parameter sets, the profile likelihood, the comparison of parameters with values from literature and additional information on the knock-out phenotypes.
(PDF)

Text S6 **LXR model development.** Derivation and equations of the adaptations and extensions included in the computational models of the LXR activated mouse.
(PDF)

Text S7 **LXR model parametrisation.** Parameters of the extended LXR models.
(PDF)

Text S8 **Supplemental analyses of the LXR model.** Additional visualisations of the parameters and fluxes of the LXR models. Simulations of a knock-out in the LXR activated models.
(PDF)

Text S9 **Flux FPLC profiles.** Flux FPLC profiles of wild-type, knock-out, and 14 days LXR activated models.
(PDF)
