# Supplementary material for: A Computational Model for the Analysis of Lipoprotein Distributions in the Mouse: Translating FPLC Profiles to Lipoprotein Metabolism
Source: PLoS Comput Biol. 2014 May 1;10(5):e1003579. doi: 10.1371/journal.pcbi.1003579 (PMC4006703; doi:10.1371/journal.pcbi.1003579)
Supplement: Text S1 — Additional calculations. Extended calculation of CE index j, calculation of the free cholesterol content and phospholipid content and overview of the compositional model. (PDF) [file pcbi.1003579.s001.pdf]

Supporting information – Text S1: Additional calculations

A computational model for the analysis of lipoprotein distributions in the mouse: Translating FPLC profiles to lipoprotein metabolism

F. L. P. Sips, C. A. Tiemann, M. H. Oosterveer, A. K. Groen, P. A. J. Hilbers, N. A. W. van Riel

This text provides additional information regarding the build-up of the lipoprotein grids. It consists of (a) a more detailed derivation and argumentation of equation (3) (Main Text), (b) the derivation and application of the interpolation functions for FC and PL determination and (c) a short overview of the complete compositional model.

Extended calculation of the CE index: *j*

In this section, we provide the derivation of equation (3) (Main text). The objective of this calculation is to find an equation that defines *CE(j)*. The equation should provide a value of # CE that is independent of *i* and that leads to a linear increase in the *log10(D)* in the progression from (i_{min}; j_{min}) to (i_{min}; j_{max}).
We note that for all other values of *i*, the increase in *log10(D)* will therefore not be strictly linear. We impose this distribution because the resolution of the FPLC profile is highest for small particles. By distributing the particles in the model in the same way as in the data a smaller grid will be sufficient to describe all particles.
First, we define $logD_{min}$ and $logD_{max}$ as the minimal and maximal values of the logarithm of particle diameter for any *j*, while $i = i_{min}$ (# TG = # $\mathrm{TG_{min}}$):

$$logD_{min} = \log_{10}(D(\#\mathrm{CE}_{min}, \#\mathrm{TG}_{min}))$$

$$logD_{max} = \log_{10}(D(\#\mathrm{CE}_{max}, \#\mathrm{TG}_{min}))$$

The diameters can be calculated using equation (1) (Main text). Using these values for the minimal and maximal logarithm of particle diameter, we can impose a linear increase in *log10 (D)* if $i = i_{min}$ by defining that equation (1) holds.

$$\log_{10}\left(D\left(\mathrm{CE}\left(j\right),\mathrm{TG}\left(i_{min}\right)\right)\right)=logD_{min}+\left(j-1\right)\cdot\frac{logD_{max}-logD_{min}}{j_{max}-1} \tag{1}$$

This equation is expressed in D instead of *log10(D)* by

$$D\left(\mathrm{CE}\left(j\right),\mathrm{TG}\left(i_{min}\right)\right)=10^{logD_{min}}\cdot 10^{\left(j-1\right)\cdot\frac{logD_{max}-logD_{min}}{j_{max}-1}} \tag{2}$$

If we now define the constants I0 and I1 as:

$I_1=\frac{logD_{max}-logD_{min}}{j_{max}-1}$ and $I_0=logD_{min}-I_1$, we can define I(j) as a linear function of *j* :

$I\left(j\right)=I_0+I_1\cdot j$, and express equation (2) as

$$D(i_{min},j)=10^{I(j)}=10^{I_0}\cdot 10^{I_1\cdot j} \tag{3}$$

So that finally, by rewriting equation (1) (main text) with equation (3), we find equation (4) for *CE(j)*(independent of the value of *i*).

$$\mathrm{CE}\left(j\right)=\frac{N_A\cdot\frac{4}{3}\pi}{8\cdot vol_{\mathrm{CE}}}\left(10^{I_0}\cdot 10^{I_1\cdot j}-2\cdot r_{ph}\right)^3-\frac{\#\mathrm{TG}_{min}\cdot vol_{\mathrm{TG}}}{vol_{\mathrm{CE}}} \tag{4}$$

Calculation of the free cholesterol and phospholipid content

For each modelled lipoprotein particle, free cholesterol content and phospholipid content must be calculated to determine total cholesterol content and phospholipid fluxes respectively.

Several compositional models of lipoproteins exist in literature for humans (notably [1], [2], [3]). In [4], a human compositional model [2] is applied to determine lipid and protein content, while alternative lipid metabolism models use straightforward assumptions on the ratio between lipids in a lipoprotein ([5], [6]). Due to the large differences in human and murine lipoprotein metabolism, these compositional models may not be applicable to murine lipoprotein composition. The absence of CETP and the relative distribution of cholesterol between HDL and LDL may especially interfere with the ability of human models to describe mouse data.

The human lipoprotein compositional models [1] and [2] were found to not be able to describe the compositional data of lipoprotein composition in C57Bl/6J mice found in [7]. Data from literature is therefore used in the framework directly to calculate the content of the remaining major lipid components.

For calculation of the free cholesterol and phospholipid content of the particles, data from two sources was used: [7] and [8]. In general, the data was transformed so that both the (natural) logarithm of the radius and the ratio of surface lipid to core lipids could be extracted. This data is used as an interpolation function by the model to determine the FC and PL contents for every lipoprotein $L_{(i;\, j)}$ of which the logarithm of the radius and the CE and TG content are known. Due to the different nature of the datasets, we will discuss the calculation separately.

In [7] the data includes (a) the distributions of sizes of lipoproteins and (b) 4 measurements of the relative lipid composition of these lipoproteins; both in 7 classes of lipoprotein. To determine the logarithm of the radius of the lipoprotein in a fraction, the diameter distribution was extracted from [7] and normalized so that the integral of the distribution equals one. 10^7 random samples of the diameter distribution were generated for each of the seven fractions. For each sample the logarithm of the radius was calculated, and finally the mean logarithm of the radius was computed by calculating the mean of all 10^7 samples per fraction.

To determine the ratios of lipids in every fraction measurements of relative lipid content were processed. The measurements of relative lipid content available per fraction were used to calculate the parameters of log-normal distributions of the relative lipid content for the PL, TG, TC and FC content, which are the measured quantities in [7]. Two outliers were removed before calculation[1]. The relative composition was first sampled 10^7 times per fraction. The sampling of the relative distributions will not necessarily result in a total content of 100 % for the combination of TG, TC and PL. Therefore the samples were normalized to 100% with only minor changes to the observed standard deviations.

The molecular mass of each lipid component of a lipoprotein as measured and reported in [9] (TG: 859.2 ± 6.1, CE: 647.9 ± 0.9 , PL: 786 ± 4.0 grams / mol) was also sampled seven x 10^7 times, assuming a normal distribution. The weight of free cholesterol (386.7 grams / mol [9]) is not dependent on fatty acid chain length and therefore considered constant. Each relative distribution

[1] 1The removed outliers are: [7], Table 2, C57Bl/6J d 1.04-1.06, sample a; and [7], Table 2, C57Bl/6J d 1.08-1.10, sample c. This leaves 4 samples in most fractions, and 3 samples in both mentioned fractions.

sample set (of the four relative mass distributions; PL, TC, TG and FC) was converted to an absolute sample set with the four molecular weights of lipids (of which three are sampled).

For each sample, a molar ratio of # PL /(# CE + # TG) and # FC /(# CE + # TG) was calculated.

Finally, the mean over the 10^7 samples per fraction was calculated for each ratio, and the results are reported in Table 1.

The eighth data point included in the interpolation function describes very large VLDL [8]. This measurement is composed of particle size (94 ± 12 nm), and molar lipid content (TG, CE, FC and PL). A conversion to molar units was not necessary, as the data was already in molar units. This data point was sampled via a normal distribution (as mean and standard deviation were given) 10^7 times and the ratios were again calculated. The diameter was calculated by sampling 10^7 diameters from an assumed normal distribution, computing the logarithm of the radius of each sample, and calculating the mean of these samples.

The final table is given in Table 1.

Table 1. Ratio of FC and PL to core lipids for eight values of log_r.

Look up tables used to calculate FC and PL. The values were derived from [7] and [8] as described. Ratios are provided as mean ± SD.

Size class	log_r	$\frac{\#PH}{\#CE+\#TG}$	$\pm$	$\frac{\#FC}{\#CE+\#TG}$	$\pm$
	log (nm)	-	-	-	-
1	3.6400	1.8495	0.1136	0.5745	0.1007
2	3.7295	2.0155	0.2292	0.9124	0.2325
3	3.7895	2.0451	0.4234	1.0771	0.2719
4	4.1628	1.0599	0.1172	0.8358	0.1664
5	4.4311	0.8287	0.1323	0.8136	0.2348
6	4.6721	0.5127	0.0782	0.6294	0.2233
7	4.9551	0.2005	0.0322	0.1143	0.0339
8	6.1444	0.1059	0.0435	0.0539	0.0207

Overview of compositional model

To reiterate, expressed in indices i and j the CE and TG content of the model is defined through equations 2 and 3 (Main text). The size of the lipoprotein is calculated through the application of equation (1) (Main text). To calculate the FC and PL content, interpolation functions were calculated based on [7] and [8], in which the distributions of molar masses as given in [9] were used to convert the samples from weight distributions to molar distributions. These are interpolated linearly over the logarithm of the radius of the lipoprotein in the model (Table 1). If the logarithm of the radius of the modelled lipoprotein is outside the described range, extrapolation is performed by taking the value of the nearest point. To calculate the FC or PL content, the interpolated ratio is multiplied by the core lipid content.

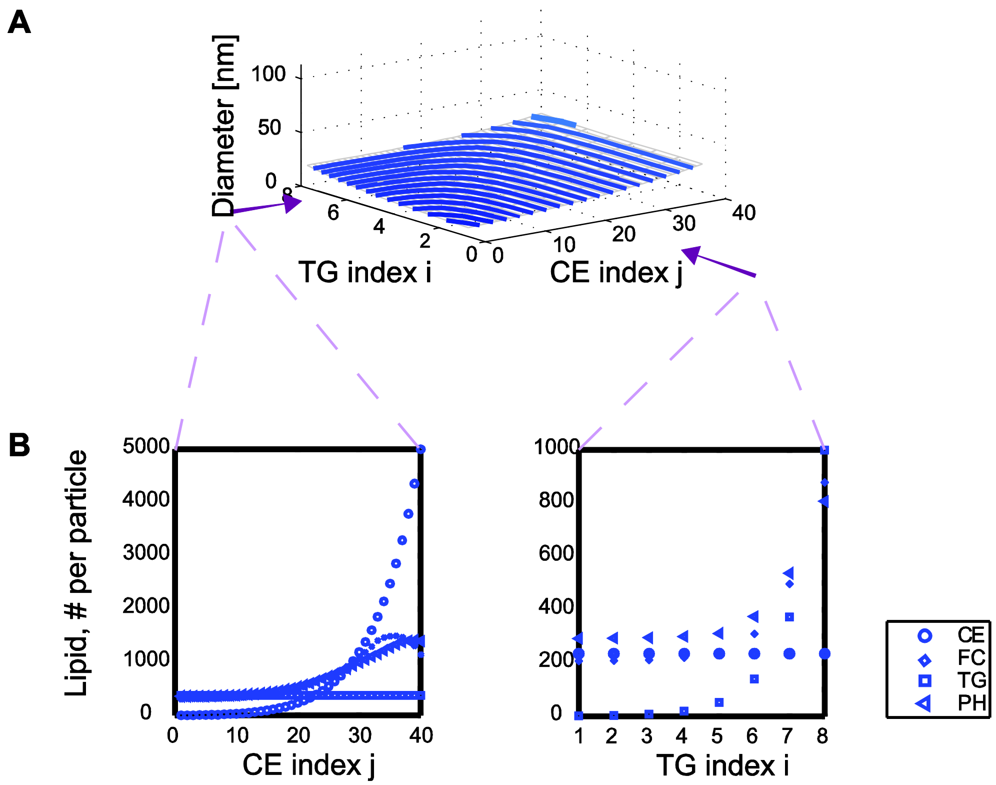

Figure 1 - Relationship between modelled lipoprotein core composition and lipoprotein surface components

A. Core composition and size of HDL particles. Figure as in Figure 1C. **B.** (left) Relationship between *j* and the four lipid components (TG, CE, FC and PL, in # of molecules) for a fixed *i* with *i* = 7. (right) Relationship between *i* (for a fixed *j* of *j* = 20) and the lipid components of the HDL particle. The figure illustrates model dimensions as well as the compositional model.

References

1. Shen BW, Scanu AM, Kézdy FJ (1977) Structure of human serum lipoproteins inferred from compositional analysis. Proc Natl Acad Sci U S A 74: 837-841.

2. Tuzikov F, Tuzikova N, Galimov R, Panin L, Nevinsky G (2002) General model to describe the structure and dynamic balance between different human serum lipoproteins and its practical application. Med Sci Monit 8: MT79-88.

3. Kumpula L (2011) Computational models and methods for lipoprotein research. Ph.D. thesis, Aalto University, Dept. of Biomedical engineering and Computational science.

4. van Schalkwijk DB, de Graaf AA, van Ommen B, van Bochove K, Rensen RCN, et al. (2009) Improved cholesterol phenotype analysis by a model relating lipoprotein life cycle processes to particle size. J Lipid Res 50: 2398-2411.

5. Hübner K, Schwager T, Winkler K, Reich JG, Holzhütter HG (2008) Computational lipidology: predicting lipoprotein density profiles in human blood plasma. PLoS Comput Biol 4: e1000079.

6. van de Pas NC (2011) A physiologically based kinetic model for the prediction of plasma cholesterol concentrations in mice and man. Ph.D. thesis, Wageningen University.

7. de Silva HV, Más-Oliva J, Taylor JM, Mahley RW (1994) Identification of apolipoprotein B-100 low density lipoproteins, apolipoprotein B-48 remnants, and apolipoprotein E-rich high density lipoproteins in the mouse. J Lipid Res 35: 1297-310.

8. Grefhorst A, Elzinga BM, Voshol PJ, Plosch T, Kok T, et al. (2002) Stimulation of lipogenesis by pharmacological activation of the liver X receptor (LXR) leads to production of large, triglyceride-rich VLDL particles. J Biol Chem 277: 34182-34190.

9. Teerlink T, Scheffer PG, Bakker SJL, Heine RJ (2004) Combined data from LDL composition and size measurement are compatible with a discoid particle shape. J Lipid Res 45: 954-966.
