# Supplementary material for: A Computational Model for the Analysis of Lipoprotein Distributions in the Mouse: Translating FPLC Profiles to Lipoprotein Metabolism
Source: PLoS Comput Biol. 2014 May 1;10(5):e1003579. doi: 10.1371/journal.pcbi.1003579 (PMC4006703; doi:10.1371/journal.pcbi.1003579)
Supplement: Text S2 — Wild-type model equations. Derivation and equations of the wild-type HDL and VLDL sub-models. (PDF) [file pcbi.1003579.s002.pdf]

Supporting information – Text S2: Wild-type model equations

A computational model for the analysis of lipoprotein distributions in the mouse: Translating FPLC profiles to lipoprotein metabolism

F. L. P. Sips, C. A. Tiemann, M. H. Oosterveer, A. K. Groen, P. A. J. Hilbers, N. A. W. van Riel

This text provides the mathematical description of the ordinary differential equations and boundary conditions - first an overview of the governing differential equations, followed by more detailed descriptions of the individual processes. Also, it provides model parameters (Table 1), nascent HDL parameters (Table 2) and an overview of model equations (Table 3).

Model structure and boundaries

The sub-models are divided into 8 by 40 and 40 by 8 compartments of lipoprotein composition, which throughout the supplemental material will be referred to as cells. The metabolism of lipoproteins in cell (*i ,j*) can be described by equation (1) for cells in the HDL grid and by equation (2) if the cell is in the VLDL grid (see equations (9) and (11) in the Main Text). Note that in the following equations, $a_{i,j}$ and $b_{i,j}$ represent the concentration of lipoproteins in cell (*i,j*) of the HDL or VLDL grid respectively.

$$\frac{d(a_{i,j})}{dt} = u_{prod|i,j} + u_{lip|i,j} + u_{sel|i,j} + u_{chol|i,j} + u_{trig|i,j} - (r_{lip|i,j} + r_{sel|i,j} + r_{chol|i,j} + r_{trig|i,j} + r_{upt|i,j})\, a_{i,j} \quad (1)$$

$$\frac{d(b_{i,j})}{dt} = u_{prod|i,j} + u_{lip|i,j} + u_{sel|i,j} - (r_{lip|i,j} + r_{sel|i,j} + r_{upt|i,j})\, b_{i,j} \quad (2)$$

For the HDL grid the input into a cell due to remodelling can be defined as:

$$u_{lip|\,i,j} = \begin{cases} r_{lip|\,i+1,j} \cdot a_{i+1,j} & if\ i \in [1, i_{max} - 1] \\ 0 & if\ i = i_{max}\ and\ j \in [1, j_{max}] \end{cases} \tag{3}$$

$$u_{sel|\,i,j} = \begin{cases} r_{sel|\,i,j+1} \cdot a_{i,j+1} & if\ j \in [1, j_{max} - 1] \\ 0 & if\ j = j_{max}\ and\ i \in [1, i_{max}] \end{cases} \tag{4}$$

$$u_{chol|\,i,j} = \begin{cases} r_{chol|\,i,j-1} \cdot a_{i,j-1} & if\ j \in [2, j_{max}] \\ 0 & if\ j = 1\ and\ i \in [1, i_{max}] \end{cases} \tag{5}$$

$$u_{trig|\,i,j} = \begin{cases} r_{lip|\,i-1,j} \cdot a_{i-1,j} & if\ i \in [2, i_{max}] \\ 0 & if\ i = 1\ and\ j \in [1, j_{max}] \end{cases} \tag{6}$$

The input into a cell is thus zero if the cell is on a boundary in the referenced direction. To close the boundaries, we also define the complementary flux, from a cell on the boundary out of the model, as 0.

$$r_{chol|i,j_{max}} = 0 \qquad i \in [1, i_{max}] \tag{7}$$

$$r_{sel|i,1} = 0 \qquad i \in [1, i_{max}] \tag{8}$$

$$r_{trig|i_{max},j} = 0 \qquad j \in [1, j_{max}] \tag{9}$$

$$r_{lip|1,j} = 0 \qquad j \in [1, j_{max}] \tag{10}$$

For the VLDL sub-model (composed of only two types of remodelling steps) we can suffice by defining:

$$u_{lip|i,j} = \begin{cases} r_{lip|i+1,j} \cdot b_{i+1,j} & if\ \ i \in [1, i_{max} - 1] \\ 0 & if\ \ i = i_{max} \end{cases} \tag{11}$$

$$\text{for} \quad \mathrm{j} \in [1, \mathrm{j}_{\mathrm{max}}]$$

$$u_{sel|i,j} = \begin{cases} r_{sel|i,j+1} \cdot a_{i,j+1} & if\ \ j \in [1, j_{max} - 1] \\ 0 & if\ \ j = j_{max} \end{cases} \tag{12}$$

$$\text{for} \quad \mathrm{i} \in [1, \mathrm{i}_{\mathrm{max}}]$$

And

$$r_{lip|1,j} = 0 \qquad j \in [1, j_{max}] \tag{13}$$

$$r_{sel|i,1} = 0 \qquad i \in [1, i_{max}] \tag{14}$$

In these equations, rate equations r (and remodelling-derived inputs u) are not constants, but phenomenological equations based on lipoprotein characteristics. These equations will be discussed individually, preceded by the full equations for lipoprotein production. VLDL metabolism will be discussed first, followed by HDL metabolism.

Table 1. **Structural model parameters**

The values of the minimal and maximal CE and TG content in a grid, i.e. the values of # TG and # CE for $i = 1$, $j = 1$, $i = i_{max}$ and $j = j_{max}$. The exponential function describing the TG content excludes a minimal value of zero and the minimal values are therefore set to one molecule. The maximal values of the HDL components are based on the values found for the TG and CE content of human HDL2 [21], which are multiplied by an approximate factor of 50 as a safety margin. The maximal values of the VLDL components are derived from the lognormal distributions of the nascent components. The values are calculated as $e^{\mu+3\sigma}$ for the lognormal distribution with the diameter of 94 nm as the value of *D*.

Name	Unit	Value	Interpretation
$i_{min,A} = i_{min,B} = j_{min,A} = j_{min,B}$	-	1	-
$i_{max,A}$	-	8	-
$j_{max,A}$	-	40	-
$i_{max,B}$	-	40	-
$j_{max,B}$	-	8	-
$TG_{min,A}$	#	1	TG_{min} in the HDL grid
$CE_{min,A}$	#	1	CE_{min} in the HDL grid
$TG_{min,B}$	#	1	TG_{min} in the VLDL grid
$CE_{min,B}$	#	1	CE_{min} in the VLDL grid
$TG_{max,A}$	#	1000	TG_{max} in the HDL grid
$CE_{max,A}$	#	5000	CE_{max} in the HDL grid
$TG_{max,B}$	#	$4.3 \cdot 10^5$	TG_{max} in the VLDL grid
$CE_{max,B}$	#	$8.4 \cdot 10^3$	CE_{max} in the VLDL grid

VLDL metabolism

Data-based VLDL production

Production of VLDL is based on a combination of literature data and variable parameters. As with all processes in the model, the production of VLDL must be defined via the triglyceride and cholesteryl ester content of the particle. The final production of VLDL in the model is calculated from: (a) experimental data of the triglyceride and cholesteryl ester content of a nascent VLDL particle, (b) the distribution of the triglyceride and cholesteryl ester content, (c) the diameter of the nascent lipoprotein and (d) the measured value of the VLDL-TG production.

(a) Experimental data: In Grefhorst *et al.*, (2002) [6] the individual lipid content in nascent VLDL is obtained from male C57Bl/6J mice.
(b) Distribution: Size profiles of nascent VLDL show evidence of a tail towards larger particles [12]. For this reason, we have chosen to model the production of VLDL with a log-normal distribution.
(c) Nascent VLDL diameter: Due to the large range of values found in literature for nascent VLDL size measured under comparable conditions (e.g. [6], 94 ± 12 nm and [7] 59.3 ± 3.6 nm), the size of the nascent VLDL is estimated from the FPLC data. We define the characteristic diameter of nascent VLDL as D.
(d) VLDL-TG production: The nascent VLDL-TG production has been measured (see again [8], [17]) and the VLDL-TG production in the model is scaled to this value.

Based on these values and functions, we can define the VLDL production into a cell by the following equation, in which $prod_B$ is the nascent lipoprotein distribution.

$$u_{prod|i,j} = scale_B \cdot \int_{\mathrm{TG}(i-0.5)}^{\mathrm{TG}(i+0.5)} \int_{\mathrm{CE}(j-0.5)}^{\mathrm{CE}(j+0.5)} prod_B(\#\mathrm{CE}, \#\mathrm{TG}, \boldsymbol{\mu}, \boldsymbol{\Sigma}) \quad \mathrm{d}\#\mathrm{CE}\ \mathrm{d}\#\mathrm{TG} \tag{15}$$

$$= scale_B \cdot \int_{i-0.5}^{i+0.5} \int_{j-0.5}^{j+0.5} prod_B(\mathrm{TG}(i), \mathrm{CE}(j), \boldsymbol{\mu}, \boldsymbol{\Sigma}) \quad \mathrm{d}j\ \mathrm{d}i \tag{16}$$

In order to determine the production of VLDL in each cell we must define the distribution $prod_B$, and calculate the values of parameter(vectors) μ, Σ and $scale_B$.
As the production function must be defined over both CE and TG content, the equation for $prod_B$

is based on the general equation for a multivariate log-normal distribution (equation (17)).

$$f(x_1, x_2; \boldsymbol{\mu}, \boldsymbol{\Sigma}) = \frac{1}{2\pi \cdot x_1 \cdot x_2 \cdot \sigma_{x_1}\sigma_{x_2}\sqrt{1-\rho^2}} e^{-\frac{1}{2\left(1-\rho^2\right)}\left[\frac{\left(ln(x_1)-\mu_{x_1}\right)^2}{2\sigma_{x_1}^2}+\frac{\left(ln(x_2)-\mu_{x_2}\right)^2}{2\sigma_{x_2}^2}-\frac{2\rho\left(ln(x_1)-\mu_{x_1}\right)\left(ln(x_2)-\mu_{x_2}\right)}{\sigma_{x_1}\sigma_{x_2}}\right]} \tag{17}$$

Where the parameter vectors $\boldsymbol{\mu}$ and $\boldsymbol{\Sigma}$ are parameters of the log-normal distribution. These parameter vectors are defined as $\boldsymbol{\mu} = \begin{bmatrix} \mu_{x_1} \\ \mu_{x_2} \end{bmatrix}$ and $\boldsymbol{\Sigma} = \begin{bmatrix} \sigma_{x_1}^2 & \rho\sigma_{x_1}\sigma_{x_2} \\ \rho\sigma_{x_1}\sigma_{x_2} & \sigma_{x_2}^2 \end{bmatrix}$.

Assuming there is no correlation between the distributions, i.e. $\rho = 0$, and substituting TG for x_1 and CE for x_2 the log-normal distribution for VLDL production can be written as:

$$prod_B(TG, CE; \boldsymbol{\mu}, \boldsymbol{\Sigma}) = \frac{1}{2\pi \cdot TG \cdot CE \cdot \sigma_{TG}\sigma_{CE}} e^{-\frac{1}{2}\left[\frac{(ln(TG)-\mu_{TG})^2}{\sigma_{TG}^2}+\frac{(ln(CE)-\mu_{CE})^2}{\sigma_{CE}^2}\right]} \tag{18}$$

with $\boldsymbol{\mu} = \begin{bmatrix} \mu_{TG} \\ \mu_{CE} \end{bmatrix}$, and $\boldsymbol{\Sigma} = \begin{bmatrix} \sigma_{TG}^2 & 0 \\ 0 & \sigma_{CE}^2 \end{bmatrix}$.

The measured metrics (the mean of the TG content ($E[\#\mathrm{TG}]$) and the variance of the TG content $Var[\#\mathrm{TG}] = SD[\#\mathrm{TG}]^2$) are related to the distribution parameters via:

$$E[\#\mathrm{TG}] = e^{\left(\mu_{\mathrm{TG}}+\frac{1}{2}\sigma_{\mathrm{TG}}^2\right)} \tag{19}$$

$$Var[\#\mathrm{TG}] = \left(e^{\sigma_{TG}^2}-1\right)e^{2\mu_{TG}+\sigma_{TG}^2} = \left(e^{\sigma_{TG}^2}-1\right)E[\#\mathrm{TG}]^2 \tag{20}$$

And therefore the distribution parameters are a function of the mean and variance of lipid content:

$$\sigma_{\mathrm{TG}} = \sqrt{ln\left(1+\frac{Var[\#\mathrm{TG}]}{E[\#\mathrm{TG}]^2}\right)} \tag{21}$$

And

$$\mu_{TG} = ln\left(E[\#\mathrm{TG}]\right) - \frac{1}{2}\sigma_{\mathrm{TG}}^2 \tag{22}$$

And analogous equations for the CE content.

If the mean and variance of the number of CE and TG particles is known, equations (21) and (22) can be applied to calculate the parameters of the log-normal distribution. However, (a) provides only the relative CE and TG content and variances relative to mean content. Via equation (1) (Main Text), and by defining a variable c which makes calculates absolute from relative lipid content, we can calculate the values of the parameters of the log-normal distribution.

We define c as the scaling factor relating the relative content of CE and TG in the core to the absolute value of lipid particles present in the lipoprotein and then calculate the parameters of the production distribution as follows:

$$E[\#\mathrm{TG}] = c \cdot \frac{[TG]}{[TG]+[CE]} = c \cdot \frac{28.53}{28.53+0.63} \tag{23}$$

$$E[\#\mathrm{CE}] = c \cdot \frac{[CE]}{[TG]+[CE]} = c \cdot \frac{0.63}{28.53+0.63} \tag{24}$$

$$r\left(D_{nascent}, r_{pl}\right) = \left(\frac{\#\mathrm{TG}_{nascent} \cdot \mathrm{vol}_{\mathrm{TG}} + \#\mathrm{CE}_{nascent} \cdot \mathrm{vol}_{\mathrm{CE}}}{\frac{4}{3}\pi N_{\mathrm{A}}}\right)^{\frac{1}{3}} \tag{25}$$

$$\frac{D_{nascent}}{2} - r_{pl} = \left(\frac{c \cdot \frac{\#\mathrm{TG}_{rel}}{core_{rel}} \cdot \mathrm{vol}_{\mathrm{TG}} + c \cdot \frac{\#\mathrm{CE}_{rel}}{core_{rel}} \cdot \mathrm{vol}_{\mathrm{CE}}}{\frac{4}{3}\pi N_{\mathrm{A}}} \right)^{\frac{1}{3}} \tag{26}$$

$$\left(\frac{D_{nascent}}{2} - r_{pl} \right)^3 = c \cdot \frac{\frac{\#\mathrm{TG}_{rel}}{core_{rel}} \cdot \mathrm{vol}_{\mathrm{TG}} + \frac{\#\mathrm{CE}_{rel}}{core_{rel}} \cdot \mathrm{vol}_{\mathrm{CE}}}{\frac{4}{3}\pi N_{\mathrm{A}}} \tag{27}$$

So that finally, variable c can be calculated via:

$$c = \frac{\left(\frac{D_{nascent}}{2} - r_{pl} \right)^3}{\left(\frac{\frac{\#\mathrm{TG}_{rel}}{core_{rel}} \cdot \mathrm{vol}_{\mathrm{TG}} + \frac{\#\mathrm{CE}_{rel}}{core_{rel}} \cdot \mathrm{vol}_{\mathrm{CE}}}{\frac{4}{3}\pi N_{\mathrm{A}}} \right)} \tag{28}$$

Following the calculation the mean and variance of the $\#\mathrm{TG}$ and $\#\mathrm{CE}$ using the result of equation (28), the parameters of the log-normal distribution (equations (21) and (22) can now be determined.

The final step of calculating VLDL production is the determination of $scale_B$. The production of VLDL must be scaled so that the production of VLDL-TG matches the measured mean value of 188 $\mu mol/kg/hour$ for the untreated mouse. This is done by calculating the current VLDL-TG production as in equation (29) and consequently scaling the VLDL production function with a value of $scaleB$ (equation (30)) to scale VLDL production correctly. The unit in which fluxes in the model are expressed is $\mu mol/kg/mol$.

$$\mathrm{VLDL} - \mathrm{TG}_{unscaled} = \sum_{j=1}^{j_{max}} \sum_{i=1}^{i_{max}} u_{prod}(i, j) \cdot \mathrm{TG}(i) \tag{29}$$

$$scale_{B,WT} = \frac{187.866}{\mathrm{VLDL} - \mathrm{TG}_{unscaled}} \tag{30}$$

VLDL remodelling

VLDL remodelling includes both lipolysis and selective uptake of CE.

Lipolysis of triglycerides in lipoproteins is mediated by several enzymes (e.g. lipoprotein lipase (LPL), hepatic lipase (HL) and endothelial lipase (EL) [13], [2]). In the model, lipolysis is described by two processes.

$$r_{lip|i,j} = r_{lip_B|i,j} + r_{lip_{A,B}|i,j} \tag{31}$$

Here, lip_B is a VLDL-specific, size-driven lipolysis which is mainly modelled on LPL activity. The equation for lip_B includes a surface area threshold and a surface area dependency because LPL is known to prefer large, TG-rich VLDL as a substrate [20], [1].

$$r_{lip_B|i,j} = \begin{cases} s(i,j) \geq s_{min} & (s(i,j) - s_{min}) \cdot c_{LPL} \cdot \mathrm{TG}(i) \cdot \dfrac{1}{\Delta_S \mathrm{TG}(i,j)} \\ s(i,j) \leq s_{min} & 0 \end{cases} \tag{32}$$

The general lipolysis equation ($lip_{A,B}$) incorporates the fact that HL and EL also have phospholipase activity [11], [13] by incorporating the ratio between TG and total TG and PL, to the power n.

$$r_{lip_{A,B}|i,j} = c_{lip,scaled} \cdot \left(\frac{\mathrm{TG}(i)}{\mathrm{TG}(i) + \mathrm{PL}(i,j)} \right)^n \cdot \mathrm{TG}(i) \cdot \frac{1}{\Delta_S \mathrm{TG}(i,j)} \tag{33}$$

Additionally, both equations include a parameter to-be-estimated (c_{lip} and c_{LPL}), the amount of triglycerides and a correction factor for the scale and non-linearity of the grid. This correction factor is defined, for the lipolysis equations, as $\Delta_S \mathrm{TG}(i,j) = \mathrm{TG}(i,j) - \mathrm{TG}(i-1,j)$.

Following the definition of parameter values, the value of parameter c_{lip} is adjusted to the value of n, to correct for the difference in the integral of $\left(\frac{\mathrm{TG}(i)}{\mathrm{TG}(i) + \mathrm{PL}(i,j)} \right)^n$ for a value of n, as follows:

$$c_{lip,scaled} = c_{lip} \cdot \frac{1}{2} \cdot (n-1)$$

It is important to note that HL in mice, in contrast to humans may not be exclusively hepatically

bound [9], [3]. The equation governing mainly HL activity therefore does not resemble e.g. the equations in [19].

VLDL selective uptake is modelled similarly to HDL selective uptake, but with different parameter values due to the differences in apolipoprotein content of the particle. In HDL selective uptake, Apo A plays an important role in SR-B1 binding [18], while this is not the case for VLDL metabolism. The equation is given by:

$$r_{sel|i,j} = c_{selB} \cdot D(i,j) \cdot \frac{\mathrm{CE}(j)}{\mathrm{CE}(j) + \mathrm{TG}(i)} \cdot \mathrm{CE}(j) \cdot \frac{1}{\Delta_A \mathrm{TC}(i,j)} \tag{34}$$

Where

$$\Delta_A \mathrm{TC}(i,j) = \mathrm{TC}(i,j) - \mathrm{TC}(i,j-1) \tag{35}$$

The equation will be explained in more detail in the HDL sub-model.

VLDL catabolism

VLDL catabolism is mediated by a number of receptors and proteins (e.g. Apo B100, Apo E, various hepatic lipoprotein receptors such as the LDL receptor, heparan sulfate proteoglycans, as well as LPL and HL (as a bridging function) [15]). Here, we define two equations, where one equation has an optimum surface area representing an optimum configuration or concentration of a lipoprotein bound protein such as Apo E on lipoproteins of an intermediate size. Following accumulation of Apo E in the delipidation process, intermediate density lipoproteins have a higher concentration of Apo E and affinity to lipoprotein receptors [5] than large VLDL, while LDL are again Apo E-poor [10]. This is modelled by an equation in which an optimum lipoprotein size for catabolism is defined by a Gaussian function. The second VLDL (or LDL) catabolism equation is constant. The equations are be given by:

$$r_{upt_B|i,j} = r_{upt_{B,c}|i,j} + r_{upt_{B,E}|i,j} \tag{36}$$

Where the constant uptake is defined as:

$$r_{upt_{B,c}|i,j} = c_{uptB} \tag{37}$$

And the surface-area based uptake is given by:

$$r_{upt_{B,E}|i,j} = \frac{A_{upt}}{\sigma_{upt}} e^{-\frac{\left(s-\mu_{upt}\right)^2}{2\sigma_{upt}^2}} \tag{38}$$

HDL metabolism

Data-based HDL production

The production of (spherical) HDL particles is a complex process. First, apoA1 is lipidated with phospholipids and free cholesterol effluxed from tissues through multiple pathways, in which ATP-binding cassette transporters are involved, [16], resulting in a discoïdal shaped particle. Following further acquisition of (free) cholesterol from tissues, free cholesterol is converted to cholesteryl ester by lecithin-cholesterol acyltransferase and the core of the particle is filled, resulting in a more spherical particle.

As only a minority of HDL has a discoïdal shape and these particles do not carry either of the core lipids that the model is based on, the model does not contain the individual processes but rather the result of HDL production. Nascent HDL in the model contain the amount of cholesterol as measured in the heterogeneous production of mouse hepatocytes observed *in vitro*.

As measurements revealed more than one type of particles being produced ([4]), both types of particles described will be included in the model. The standard deviation of the data is again used to determine a distribution of nascent HDL particles, $prod_A$. The total production of HDL is the sum of the particles produced of each type.

In the data, the number of cholesterol present on a nascent particle is available. This is translated to a particle with minimal TG (minimal in the TG grid) and a value of CE such that the total cholesterol ($CE + FC$) will be equal to the measured value of cholesterol in the nascent particle. To create the distributions, samples (10^6) are taken from normal distributions with mean TC and standard deviation as given in Table 2. These samples are distributed over the grid (cells with TG = TG_{min}) and the distribution is normalized to the amount of samples. The area under a distribution $prod_{A,k}$ is therefore always one.

$$u_{prod|i,j} = scale_A \cdot \sum_{k=1}^{k_{max}} ratio_{A,k} \cdot prod_{A,k}(i, j) \tag{39}$$

Where we define $scale_A$, like as the total production of HDL particles in the model. In order for this definition to be applicable, the values of $ratio_{A,k}$ must satisfy:

$$\sum_{k=1}^{k_{max}} ratio_{A,k} = 1 \tag{40}$$

Table 2 : Characteristics of the nascent HDL particles .

The distributions of C in nascent particles as derived from [4]. The TC content was calculated by multiplying the FC to Apo A1 ratio with the average Apo AI content. So for large HDL, multiply 39 FC : 1 Apo A1 by 3.5 to obtain a mean of 136.5 particles and a standard deviation of 14. Data from [4]

Fraction	ApoA-1 number	Cholesterol molecules	Hydrodynamic diameter, [4]
	molecules	molecules	nm
Large nascent HDL	3.5	136.5 ± 14	12 ± 0.4
Small nascent HDL	2	24 ± 2	9 ± 0.5

HDL remodelling

As previously discussed, HDL can be remodelled by 4 processes: cholesterol uptake, selective cholesteryl ester uptake, lipolysis and triglyceride uptake. Each process will be discussed in this section.

Cholesterol uptake by HDL is a complex process in which several periphery-bound and several HDL-bound particles play an important role. Peripheral cholesterol efflux is mediated by e.g. ATP binding cassette A1 and ATP binding cassette G1 [16], [14]. Esterification is performed by lecithin-cholesterol acyltransferase. The phospholipid transfer protein PLTP transfers phospholipids necessary for HDL maturation from the surplus of phospholipids on (shrinking) VLDL to the HDL surface layer [16], [14]. Cholesterol accumulation by HDL is modelled based on a combination of variable parameters and a flux of phospholipids. The rate of phospholipid release from the VLDL grid due to lipolysis $y_{B,surfremn}$ is multiplied with a factor of $\frac{D(i,j)}{4}$. This is done to account for the fact that large particles will gain more CE relative to the increase in surface area than small particles, because the volume of the core rises faster with increasing diameter than the surface area. We note that the peripheral efflux to discoïdal particles is included in the HDL production flux in the model.

$$r_{chol|i,j} = c_{chol} \cdot y_{B,surfremn} \cdot \frac{D(i,j)}{4} \cdot \frac{1}{\Delta_D \mathrm{TC}(i,j)} \tag{41}$$

$$\Delta_D TC(i,j) = \mathrm{TC}(i,j+1) - \mathrm{TC}(i,j) \tag{42}$$

$$\mathrm{TC}(i,j) = \mathrm{CE}(i,j) + \mathrm{FC}(i,j) \tag{43}$$

Selective uptake of CE from HDL is mediated by SR-B1 [14], [16], [18]. The size and composition have been found to influence selective uptake activity, with large, spherical HDL having a higher affinity than smaller HDL [18]. The rate of uptake in the model is, in addition to the size of the lipoprotein, also assumed to depend on absolute CE content and relative CE content to total core lipids. The rate equation thus consists of parameter c_{selA}, diameter $D(i,j)$, the ratio between CE and core lipids, and the absolute amount of core CE.

$$r_{sel|i,j} = c_{selA} \cdot D(i,j) \cdot \frac{\mathrm{CE}(j)}{\mathrm{CE}(j) + \mathrm{TG}(i)} \cdot \mathrm{CE}(j) \cdot \frac{1}{\Delta_A \mathrm{TC}(i,j)} \tag{44}$$

$$\Delta_A \mathrm{TC}(i,j) = \mathrm{TC}(i,j) - \mathrm{TC}(i,j-1) \tag{45}$$

For **uptake of triglycerides** by the HDL particle, similar as to the uptake of cholesterol from tissue by the particle, we assume that transportation of lipids from triglyceride-rich lipoproteins to HDL plays an important role. Therefore, the rate of surface remnant generation from VLDL determines, via parameter c_{trig}, the rate of triglyceride accumulation. Note that here, it is not the growth of the surface layer, but directly of the core, which is modelled, as now a core molecule is transported instead of a surface particle.

$$r_{trig|i,j} = c_{trig} \cdot y_{B,surfremn} \cdot \frac{1}{\Delta_W \mathrm{TG}(i,j)} \tag{46}$$

$$\Delta_W \mathrm{TG}(i,j) = \mathrm{TG}(i+1,j) - \mathrm{TG}(i,j) \tag{47}$$

Lipolysis in HDL is modelled with the equation (and the same parameters) for the general lipolysis as described in VLDL metabolism, equation (33).

HDL catabolism

HDL catabolism takes place in the kidney and liver and follows delipidation of ApoA [14]. Kidney catabolism of HDL involves glomular filtration and can therefore only take place with small particles [14]. In the model, catabolism of HDL is assumed to include kidney filtration of small HDL, delipidated ApoA catabolism and delipidation of HDL (as lipid-poor ApoA are not described in the model) and thus consists of several highly size-dependent processes. For this reason, HDL catabolism is modelled to depend on HDL size (equation 48).

$$r_{upt|i,j} = c_{uptA} \cdot \frac{1}{D(i,j)} \tag{48}$$

Following the definition of HDL catabolism, the complete ODE model has been defined. The model contains 6 main HDL processes, described by 6 equations (39, 41, 46, 44, 33 and 48). The VLDL sub-model yields an addition of 4 processes, but again represented by 6 equations (15, 34, 32, 33, 37, 38). The model contains a total of 16 parameters which remain to be determined, of which 6 are found only in the HDL sub-model and 8 are found only in the VLDL sub-model; 2 parameters appear in both models.

The sub-models are connected not only via the summed comparison to the FPLC, but also through the translocation of phospholipids from VLDL remodelling to HDL remodelling (equation 41) and through a common lipolysis process (equation 33).

Table 3 – Basal lipoprotein metabolism model equations

**VLDL sub-model**	
VLDL production	$u_{prod\|i,j} = scale_B \cdot \int_{\mathrm{TG}(i-0.5)}^{\mathrm{TG}(i+0.5)} \int_{\mathrm{CE}(j-0.5)}^{\mathrm{CE}(j+0.5)} prod_B(\#\mathrm{CE}, \#\mathrm{TG}, \mu, \Sigma) \quad d\#\mathrm{CE}\ d\#\mathrm{TG}$
VLDL lipolysis (1)	$r_{lip_B\|i,j} = \begin{cases} s(i,j) \ge s_{min} & \left(s(i,j) - s_{min}\right) \cdot c_{LPL} \cdot \mathrm{TG}(i) \cdot \dfrac{1}{\Delta_S \mathrm{TG}(i,j)} \\ s(i,j) \le s_{min} & 0 \end{cases}$
VLDL lipolysis (2)	$r_{lip_{A,B}\|i,j} = c_{lip,scaled} \cdot \left(\dfrac{\mathrm{TG}(i)}{\mathrm{TG}(i) + \mathrm{PL}(i,j)} \right)^n \cdot \mathrm{TG}(i) \cdot \dfrac{1}{\Delta_S \mathrm{TG}(i,j)}$ [a]
VLDL selective uptake	$r_{sel\|i,j} = c_{selB} \cdot D(i,j) \cdot \dfrac{\mathrm{CE}(j)}{\mathrm{CE}(j) + \mathrm{TG}(i)} \cdot \mathrm{CE}(j) \cdot \dfrac{1}{\Delta_A \mathrm{TC}(i,j)}$
VLDL uptake (1)	$r_{upt_{B,c}\|i,j} = c_{uptB}$
VLDL uptake (2)	$r_{upt_{B,E}\|i,j} = \dfrac{A_{upt}}{\sigma_{upt}} e^{-\frac{\left(s - \mu_{upt}\right)^2}{2\sigma_{upt}^2}}$
**HDL sub-model**	
HDL production	$u_{prod\|i,j} = scale_A \cdot \sum_{k=1}^{k_{max}} ratio_{A,k} \cdot prod_{A,k}(i,j)$ [b]
HDL selective uptake	$r_{sel\|i,j} = c_{selA} \cdot D(i,j) \cdot \dfrac{\mathrm{CE}(j)}{\mathrm{CE}(j) + \mathrm{TG}(i)} \cdot \mathrm{CE}(j) \cdot \dfrac{1}{\Delta_A \mathrm{TC}(i,j)}$ [c]
HDL C uptake	$r_{chol\|i,j} = c_{chol} \cdot y_{B,surfremn} \cdot \dfrac{D(i,j)}{4} \cdot \dfrac{1}{\Delta_D \mathrm{TC}(i,j)}$ [d]
HDL TG uptake	$r_{trig\|i,j} = c_{trig} \cdot y_{B,surfremn} \cdot \dfrac{1}{\Delta_W \mathrm{TG}(i,j)}$ [e]
HDL uptake	$r_{upt\|i,j} = c_{uptA} \cdot \dfrac{1}{D(i,j)}$

a. $\Delta_S \mathrm{TG}(i,j) = \mathrm{TG}(i,j) - \mathrm{TG}(i-1,j)$, note that $c_{lip,scaled} = c_{lip} \cdot \frac{1}{2} \cdot (n-1)$

b. $\sum_{k=1}^{k_{max}} ratio_{A,k} = 1$

c. $\Delta_A \mathrm{TC}(i, j) = T\mathrm{C}(i, j) - T\mathrm{C}(i, j-1)$

d. $\Delta_D \mathrm{TC}(i, j) = \mathrm{TC}(i, j+1) - \mathrm{TC}(i, j)$

e. $\Delta_W \mathrm{TG}(i, j) = \mathrm{TG}(i+1, j) - \mathrm{TG}(i, j)$

References

[1] Adiels M (2004) Compartmental models of lipoprotein kinetics. Ph.D. thesis, Chalmers University of Technology.

[2] Annema W, Tietge U (2011) Role of hepatic lipase and endothelial lipase in high-density lipoproteinmediated reverse cholesterol transport. Curr Atheroscler Rep 13: 257-265.

[3] Dallinga-Thie GM, Zonneveld-de Boer AJ, van Vark-van der Zee LC, van Haperen R, van Gent T, et al. (2007) Appraisal of hepatic lipase and lipoprotein lipase activities in mice. J Lipid Res 48: 2788-2791.

[4] Duong PT, Collins HL, Nickel M, Lund-Katz S, Rothblat GH, et al. (2006) Characterization of nascent HDL particles and microparticles formed by ABCA1-mediated efflux of cellular lipids to apoA-I. J Lipid Res 47: 832-843.

[5] Ehnholm C, editor (2009) Cellular Lipid Metabolism. Heidelberg: Springer, 394 pp.

[6] Grefhorst A, Elzinga BM, Voshol PJ, Plosch T, Kok T, et al. (2002) Stimulation of lipogenesis by pharmacological activation of the liver X receptor (lxr) leads to production of large, triglyceride-rich VLDL particles. J Biol Chem 277: 34182-34190.

[7] Grefhorst A, Hoekstra J, Derks TGJ, Ouwens DM, Baller JFW, et al. (2005) Acute hepatic steatosis in mice by blocking -oxidation does not reduce insulin sensitivity of very-low-density lipoprotein production. Am J Physiol Gastrointest Liver Physiol 289: G592-G598.

[8] Grefhorst A, Oosterveer MH, Brufau G, Boesjes M, Kuipers F, et al. (2012) Pharmacological LXR activation reduces presence of SR-B1 in liver membranes contributing to LXR-mediated induction of HDL-cholesterol. Atherosclerosis 222: 382 - 389.

[9] Peterson J, Bengtsson-Olivecrona G, Olivecrona T (1986) Mouse preheparin plasma contains high levels of hepatic lipase with low affinity for heparin. Biochim Biophys Acta 878: 65-70.

[10] Kumpula L (2011) Computational models and methods for lipoprotein research. Ph.D. thesis, Aalto University, Dept. of Biomedical engineering and Computational science.

[11] McCoy MG, Sun GS, Marchadier D, Maugeais C, Glick JM, et al. (2002) Characterization of the lipolytic activity of endothelial lipase. J Lipid Res 43: 921-929.

[12] Okazaki H, Goldstein JL, Brown MS, Liang G (2010) LXR-SREBP-1c-phospholipid transfer protein axis controls very low density lipoprotein (VLDL) particle size. J Biol Chem 285: 6801-6810.

[13] Packard CJ, Shepherd J (1997) Lipoprotein heterogeneity and apolipoprotein b metabolism. Arterioscler Thromb Vasc Biol 17: 3542-3556.

[14] Rader DJ (2006) Molecular regulation of hdl metabolism and function: implications for novel therapies. J Clin Invest 116: 3090-3100.

[15] Rensen P, Herijgers N, Netscher M, Meskers S, van Eck M, et al. (1997) Particle size determines the specificity of apolipoprotein e-containing triglyceride-rich emulsions for the ldl receptor versus hepatic remnant clearance in vivo. J Lipid Res 38: 1070-1084.

[16] Rothblat GH, Phillips MC (2010) High-density lipoprotein heterogeneity and function in reverse cholesterol transport. Curr Opin Lipidol 21: 0957-9672.

[17] Tiemann CA, Vanlier J, Oosterveer MH, Groen AK, Hilbers PAJ, et al. (2013) Parameter trajectory analysis to identify treatment effects of pharmacological interventions. PLoS Comput Biol 9: e1003166.

[18] Valacchi G, Sticozzi C, Lim Y, Pecorelli A (2011) Scavenger receptor class b type i: a multifunctional receptor. Ann N Y Acad Sci 1229: E1-E7.

[19] van Schalkwijk DB, de Graaf AA, van Ommen B, van Bochove K, Rensen RCN, et al. (2009) Improved cholesterol phenotype analysis by a model relating lipoprotein life cycle processes to particle size. J Lipid Res 50: 2398-2411.

[20] Xiang SQ, Cianone K, Kalant D, Sniderman AD (1999) Differential binding of triglyceride-rich lipoproteins to lipoprotein lipase. J Lipid Res 40: 1655-1662.

[21] Shen BW, Scanu AM, Kézdy FJ (1977) Structure of human serum lipoproteins inferred from compositional analysis. Proc Natl Acad Sci U S A 74: 837-841.
