# Supplementary material for: A Computational Model for the Analysis of Lipoprotein Distributions in the Mouse: Translating FPLC Profiles to Lipoprotein Metabolism
Source: PLoS Comput Biol. 2014 May 1;10(5):e1003579. doi: 10.1371/journal.pcbi.1003579 (PMC4006703; doi:10.1371/journal.pcbi.1003579)
Supplement: Text S3 — FPLC profile calculation. Steps 2 and 3 of the calculation of the FPLC profile. Both the calculation of the concentration FPLC profiles and the calculation of the flux FPLC profiles is explained. (PDF) [file pcbi.1003579.s003.pdf]

Supporting information – Text S3: FPLC profile calculation

A computational model for the analysis of lipoprotein distributions in the mouse: Translating FPLC profiles to lipoprotein metabolism

F. L. P. Sips, C. A. Tiemann, M. H. Oosterveer, A. K. Groen, P. A. J. Hilbers, N. A. W. van Riel

For calculation of the *in silico* FPLC profile, several calculations must be performed. As the size of the lipoproteins is used to calculate the profile, first the size of (1) lipoproteins in the computational framework and (2) lipoproteins in the FPLC profile must be determined. Following this, the amount of each type of lipid present in lipoproteins of the size corresponding a fraction must be calculated from the model (3).

Step 1 has been explained for the model in equation (1) (Main Text). In this section, we will first determine the size of lipoproteins in the FPLC profile. Finally, we will convert the modelled lipoprotein population to a comparable FPLC profile.

Step 2: Lipoprotein diameter in the FPLC fractions

The size of particles eluted from an FPLC can be estimated by determining the parameters a and b of the function in equation (1), [5]. For the obtained lipoprotein profiles, this relationship was calculated using the precise position of the main lipoprotein classes and the sizes of these classes, obtained from literature. Due to the need to compare with literature values, this calculation can be performed for the control/untreated mouse, and extrapolated to the treated mouse profiles.

$$^{10}log\,\mathrm{D} = a + b \cdot k_{av}' \qquad (1)$$

Where the variable k_{av}' is the relative elution coefficient, defined in our case to be between 0 and 1. Before further calculations are performed, every fraction boundary is expressed by the corresponding k_{av}' value; as there are 52 fractions, the k_{av}' values of the boundaries increase from 0 to 1 with steps of $\frac{1}{52}$. The centre of the first fraction can therefore be described by

$k_{av}'(1) = \frac{0.5}{52}$.

To most accurately determine the position of the VLDL, LDL and HDL peaks in the (control mouse) FPLC, the peaks were determined in both the cholesterol and triglyceride profiles simultaneously. A series of three Gaussian functions (equation (2)) is defined so that the means and standard deviations μ_{VLDL}, μ_{LDL}, μ_{HDL} and σ_{class} are the same for both the cholesterol and triglyceride profiles, but the amplitudes $A_{lipid,class}$ are defined separately for each combination of $lipid$, i.e. cholesterol or triglyceride, and $class$, i.e. VLDL, LDL and LDL.

$$G(\boldsymbol{\mu}, \boldsymbol{\sigma}, \mathbf{A}) = \sum_{class=1}^{3} \frac{A(class, lipid)}{\sigma(class)\sqrt{2\pi}} e^{-\frac{(x-\mu(class))^2}{2\sigma(class)^2}} \quad (2)$$

The two equations (a sum of Gaussian for both TG and TC) together thus contain 12 variables, which are estimated by fitting the equations on the data by minimizing the difference between the data in each fraction and the function via the Matlab function *lsqnonlin*. To account for differences in the order of magnitude between the triglyceride and cholesterol data, the difference is weighed by the root mean squared of the profile of the lipid. The obtained parameters are therefore the result of the minimization of a cost function as described in equation (3).

$$p_{opt} = arg \min_{p} CF(p, d) \quad (3)$$

with $\mathbf{p} = \begin{bmatrix} \boldsymbol{\mu} \\ \boldsymbol{\sigma} \\ \mathbf{A} \end{bmatrix}$, $CF(p,d) = \sum_{k=1}^{N} \frac{d_L - G(\mu, \sigma, A)}{w_L}$ and $w_L = \sqrt{\frac{1}{N}\sum_{k=1}^{N} d_L^2}$ if N is the number of fractions (52) and the function G is the function given in equation (2). Additionally, we define d_L as a data point - i.e. fraction lipid content measurement - of the lipid "L" which may be either triglyceride or cholesterol.

Lipoprotein size can be determined via multiple methods, and the method employed may influence the result [2],[4]. Size of the main classes of lipoproteins is also known to vary between strain of mice [1] and possibly gender [3]. To determine the size of these lipoproteins, therefore, a dataset of major lipoprotein class sizes of male C57Bl/6J mice that have been determined during an HPLC measurement were used (as published in [3]).

Calculating the parameters of equation 1 with the class sizes determined in [3] for male, control mice and means calculated as described with a weighted least squares method results in parameter values of $a = 1.97$ and $b = -1.95$ [1], with corresponding major class sizes of 41.5 nm, 22.6 nm and 11.3 nm for VLDL, LDL and HDL respectively.

Step 3: Calculation of profile from lipoprotein grids

For calculation of the profile from the lipoprotein grids, the lipids present in lipoproteins which have a size that falls between the sizes corresponding to the fraction boundaries must be calculated. Before further processing, the concentrations of lipoproteins are converted from μ mol / kg (fluxes are expressed in μ mol / kg / hour) to nmol / 200 μ L plasma. The untreated C57Bl/6J mice used in this study have a bodyweight of 24.2 grams and a plasma volume of 1.0 mL. The treated mice have a bodyweight of 23.75 grams and a plasma volume of 0.98 mL.

The boundaries of the FPLC fractions are calculated by applying equation 1 to the k_{av}' values of the fraction boundaries in order to calculate the boundary diameters. These boundaries, D_{bound} are used to define the function relating index i to index j so that the function describes the fraction boundary. Numerical integration over each cell was used to determine which ratio of a cell should empty into the appropriate fraction. An integration using $10^4 + 1$ values was used for numerical integration in each cell. For each cell, the relative content to belong to a fraction was multiplied with the lipoprotein content and lipid content of the cell to determine the amount of lipid that the current cell places in the current FPLC fraction. By summing this over all cells for each fraction, the total profile is obtained (equation 4)

$$L_k = \sum_{i=1}^{i=i_{max,A}} \sum_{j=1}^{j=j_{max,A}} frac_{a_{i,j} \to k} \cdot L(A,i,j) \cdot a_{(i,j)} + \sum_{i=1}^{i=i_{max,B}} \sum_{j=1}^{j=j_{max,B}} frac_{b_{i,j} \to k} \cdot L(B,i,j) \cdot b_{(i,j)} \qquad (4)$$

In this equation

---

[1] The location of the peaks was determined to be $k_{av}'(VLDL) = 0.18031$, $k_{av}'(LDL) = 0.31565$ and $k_{av}'(HDL) = 0.47086$. The measured size of the classes in [3] is: $D(VLDL) = 41.3 \pm 0.3$, $D(LDL) = 24.3 \pm 0.1$ and $D(HDL) = 10.9 \pm 0.1$. Values of $a = 1.96892588423361$ and $b = -1.94728821239495$ were used for the calculations.

- $i_{max}(A)$ and $j_{max}(A)$ are the maximal values of *i* and *j* in the HDL grid
- $frac_{a_{i,j} \rightarrow k}$ is the fraction of the area of cell (*i, j*) that falls within the boundaries of fraction k in terms of lipoprotein size. In this calculation, the axes and boundaries are expressed in terms of *i* and *j* - and the total area of a cell is therefore 1. Cell (*i, j*) is defined as between $i-0.5$ and $i+0.5$, and between $j-0.5$ and $j+0.5$. Thus, the total model runs from i-values of 0.5 (as the centre of the first cell is at $i=1$) to $i_{max}+0.5$).
- $L(A, i, j)$ is the number of lipids present in a lipoprotein particle found in cell (*i, j*) of the HDL grid. Note that this is always calculated via the centre of the cell, i.e. the CE content of the cell is $CE(i)$ and the TG content of the cell is $TG(j)$. Further lipoprotein properties of the cell are calculated from these central CE and TG values.
- $a_{i,j}$ is the concentration of lipoproteins in cell (*i, j*) of the HDL grid.
- The second term is the VLDL sub-model equivalent of the first, where every B points to the VLDL grid.

An analogous equation - where the number of lipids per particle is not included - yields the number of particles in a fraction:

$$L_k = \sum_{i=1}^{i=i_{max}(A)} \sum_{j=1}^{j=j_{max}(A)} frac_{a_{i,j} \rightarrow k} \cdot a_{(i,j)} + \sum_{i=1}^{i=i_{max}(B)} \sum_{j=1}^{j=j_{max}(B)} frac_{b_{i,j} \rightarrow k} \cdot b_{(i,j)} \quad (5)$$

Calculation of Flux-FPLC

In addition to calculation of concentration or particle number FPLC profiles, MuLiP can be used to create *flux* FPLC profiles. The calculation of flux FPLC profiles differs from equation (4), and are calculated as follows

$$L_{k,A} = \sum_{i=1}^{i=i_{max}(A)} \sum_{j=1}^{j=j_{max}(A)} frac_{a_{i,j} \rightarrow k} \cdot \phi_{A(i,j)} \Big/ \Phi_A$$
$$L_{k,B} = \sum_{i=1}^{i=i_{max}(B)} \sum_{j=1}^{j=j_{max}(B)} frac_{b_{i,j} \rightarrow k} \cdot \phi_{B(i,j)} \quad (6)$$

- Catabolism / production FPLC profiles are calculated by defining in equation (6) $\phi_{A(i,j)}$ as the rate at which lipoproteins disappear from / appear in a cell in μmol / kg / hour.
- Remodelling flux FPLC profiles are calculated by setting $\phi_{A(i,j)}$ as the rate at which the remodelling flux in a certain cell is resulting in the loss of a lipid – i.e. the rate at which TG is being removed from the particles due to the lipolysis remodelling flux out of a cell.
- HDL FPLC profiles are scaled with the total rate of lipoprotein production (μmol lipoproteins / kg / hour) Φ_A.

References

[1] Jiao S, Cole TG, Kitchens RT, Peger B, Schonfeld G (1990) Genetic heterogeneity of lipoproteins in inbred strains of mice: Analysis by gel-permeation chromatography. Metabolism 39: 155 - 160.

[2] Mora S (2009) Advanced lipoprotein testing and subfractionation are not (yet) ready for routine clinical use. Circulation 119: 2396-2404.

[3] Pratt SM, Chiu S, Espinal GM, Shibata NM, Wong H, et al. (2010) Mouse hepatic lipase alleles with variable effects on lipoprotein composition and size. J Lipid Res 51: 1035-1048.

[4] van Bochove K, van Schalkwijk DB, Parnell LD, Lai CQ, Ordovs JM, et al. (2012) Clustering by plasma lipoprotein profile reveals two distinct subgroups with positive lipid response to fenofibrate therapy. PLoS One 7: e38072.

[5] Amersham Biosciences (2002) Gel filtration: principles and methods, Edition AI. Handbook code no. 18-1022-18, Uppsala, Sweden: Amersham Biosciences. 123 p.
