# Supplementary material for: A Computational Model for the Analysis of Lipoprotein Distributions in the Mouse: Translating FPLC Profiles to Lipoprotein Metabolism
Source: PLoS Comput Biol. 2014 May 1;10(5):e1003579. doi: 10.1371/journal.pcbi.1003579 (PMC4006703; doi:10.1371/journal.pcbi.1003579)
Supplement: Text S4 — Wild-type model parametrisation. In this text the cost function, the parameter transformation, model simulation and the analysis of the results are discussed. Also, the resulting parameter values are reported. (PDF) [file pcbi.1003579.s004.pdf]

Supporting information – Text S4: Wild-type model parametrisation

A computational model for the analysis of lipoprotein distributions in the mouse: Translating FPLC profiles to lipoprotein metabolism

F. L. P. Sips, C. A. Tiemann, M. H. Oosterveer, A. K. Groen, P. A. J. Hilbers, N. A. W. van Riel

The model described in Text S2 can be generally written as:

$$\frac{dx(t,\theta)}{dt} = f(x(t,\theta),\theta,u(\theta)) \tag{1}$$

Where *x* are the state variables of the model, i.e. the lipoprotein concentrations in each cell of both grids (μ mol / kg), *t* represents time (hours), θ are the model parameters, and *u* are the model inputs of VLDL and HDL production, which are not time dependent but do depend on several model parameters (*D*, $scale_A$). The initial conditions, x_0 are defined at t = 0, $x(t_0,\theta) = x_0$.
The model outputs, i.e. the *in silico* lipoprotein profiles, can thus be written as:

$$y(t,\theta) = g(x(t,\theta),\theta,u(\theta)) \tag{2}$$

The complete model (based on the grid dimensions as given in Table 1 of Text S2) of 8x40 and 40x8, consists of 2x320 differential equations. 16 parameters (parameter vector θ) need to be defined and are estimated from control C57Bl/6J data, by minimizing the cost function S_d as follows:

$$\theta_{opt} = arg \min_{\theta} S_d(\theta) \tag{3}$$

In this text, we will consecutively describe (a) the cost function, (b) the parameter transformations, (c) the optimization procedure and (d) model acceptability.

Cost function

The cost function S_d is based on the sum of squared errors between the *in silico* FPLC and the data. In the absence of a measure of uncertainty of the data, the sum of squared errors is weighted by factors derived from the data and literature data. Two additional penalties are imposed to ensure a fast optimization and a steady state.

The cost function is defined as:

$$S_d(\theta) = \begin{cases} \sum_{k=1}^{k=N} \left(\frac{y_k(\theta) - d_k}{s_k} \right)^2 + P_k(\theta) & \text{if} Z(\theta) = 1 \\ 10^{12} & \text{if} Z(\theta) = 0 \end{cases} \tag{4}$$

Where N is the number of FPLC fractions included in the cost function, d_k is a vector containing both the lipid concentrations in each fraction of the dataset, $y_k(\theta)$ is a vector containing both the lipid concentrations in fractions of the *in silico* profile, s_k are the weighting factors which will be described below, $P_k(\theta)$ is a penalty for the appearance of particles in unphysiological boundaries of the grids and $Z(\theta) = 1$ if the model is in an approximate steady state, and $Z(\theta) = 0$ if the model is found not to be in steady state. A second check of steady state behaviour was performed for the obtained optima following optimization. The value of N is set to 35, as this is calculated to be the highest FPLC fraction that the HDL sub-model can fill.

The penalty $P_k(\theta)$ is defined as a multiplication of the sum of squared errors with the percentage of particles in a grid that is found in the unphysiological boundaries of the grids. The final value of the content in these boundary cells is $< 1\%$ in found optima, and the penalty is implemented only to speed up optimization as during parameter perturbation in optimization often large amounts of particles were found to reside against the boundaries.

$$P_k(\theta) = \left[\frac{\sum_{m=1}^{m=i_{max,A}} \sum_{n=1}^{n=j_{max,A}} a_{m,n,boundary}}{\sum_{m=1}^{m=i_{max,A}} \sum_{n=1}^{n=j_{max,A}} a_{m,n}} + \frac{\sum_{k=1}^{k=i_{max,B}} \sum_{l=1}^{l=j_{max,B}} b_{k,l,boundary}}{\sum_{k=1}^{k=i_{max,B}} \sum_{l=1}^{l=j_{max,B}} b_{k,l}} \right] \cdot \sum_{k=1}^{k=N} \left(\frac{y_k(\theta) - d_k}{s_k} \right)^2 \quad (5)$$

with

$$a_{m,n,boundary} = \begin{cases} 0 & \text{if} \quad m < i_{max} \quad OR \quad n < j_{max} \\ a_{m,n} & \text{if} \quad m == i_{max} \quad OR \quad n == j_{max} \end{cases} \quad (6)$$

$$b_{k,l,boundary} = \begin{cases} 0 & \text{if} \quad k > 1 \quad OR \quad l > 1 \\ b_{k,l} & \text{if} \quad k == 1 \quad OR \quad l == 1 \end{cases} \quad (7)$$

Therefore, the boundaries are defined as all cells in the HDL grid containing either the maximum triglyceride or the maximum cholesteryl ester content, and all cells in the VLDL grid containing either the minimal triglyceride or the minimal cholesteryl ester content.

The weighting factors (s_k) are based on a combination of two factors. First, to account for differences in triglyceride and cholesterol variability, literature values of plasma lipid measurements and their uncertainty are used to determine a relative weighting factor r_L. As shown in Table 1, r_L is determined by:

$$s_k = r_L \cdot d_k + a_L \quad (8)$$

Secondly, we take advantage of the data on FPLC fractions that do not correspond to model lipoproteins to determine an absolute factor a_L. According to the calculation of FPLC particle size, in fractions 38 and higher all particles have a diameter of less than 4 nm (twice the phospholipid bilayer) and therefore do not contain lipoproteins as described by the model. For each of these high fractions we calculate the mean in each fraction over all seven (control, 1d, 2d, 4d, 1w, 2w, 3w) profiles. We then calculate a standard deviation over all 15 included fractions (38-52) and time points, by subtracting from every measurement the mean of that fraction over seven profiles. This calculation yields two separate standard deviations: one for the cholesterol and one for the triglyceride measurements which are representative of the uncertainty of the measurements. By finally subtracting from this standard deviation the relative weighting factor times the mean

fraction content, we obtain a value (a_L) that is added to the relative weighting factor.

Table 1: Determination of weighing factors s .

Values marked with * - were taken from [3]. Further information is found in the text.

		Triglycerides	Total cholesterol
Plasma concentration	(mg/dL)	50.5 ± 17.8*	70.1 ± 4.8*
$\frac{\sigma}{\mu}$	-	0.3529	0.1937
r_L	-	0.3529	0.1937
a_L	(nmol/fraction)	0.1276	0.2196

Parameter transformation

In order to obtain a good parameter estimate, the model parameters as optimized by *lsqnonlin* should be in a similar order of magnitude. The rate constants in the model are presumed to be between an order of week^{-1} (a rate constant of $4 \cdot 10^{-3}$) and ms^{-1} (a rate constant of $2.5 \cdot 10^{6}$), and therefore for the parameters a range spanning many orders of magnitude is expected (See Table 2). Meanwhile, parameters with a physiological or mathematical definition, such as lipoprotein surface area or a ratio between particle types, can be expected to lie in a range bounded by physiological and mathematical constraints. For this reason, the parameters are separated into two categories for transformation, with different expected distributions and therefore different transformation functions.

The first category contains kinetic parameters. Due to the expected logarithmic distribution of kinetic parameter values they are transformed by taking the (natural) logarithm of the parameter. This results in a range of roughly between -20 and 25, depending on the untransformed values of the bounds (Table 2). In addition to the kinetic parameters, also parameter n is assumed logarithmically distributed.

$$\theta_{i,1,t} = \ln(\theta_{i,1}) \tag{9}$$

The second category contains parameters with a geometrical or mathematical relevance. These parameters will be divided by the upper bound of the parameter, for a range between 0 and 1.

$$\theta_{i,2,t} = \frac{\theta_{i,2}}{u_i} \tag{10}$$

When taking random samples of parameters, the lower and upper bounds and expected distributions are also taken into account. For this reason, we also define here a random sample for a logarithmically distributed parameter:

$$\theta_{i,1,t,init} = \ln(l_i) + rand \cdot (\ln(u_i) - \ln(l_i)) \tag{11}$$

where *rand* is a random sample from a uniform distribution between 0 and 1.
For the second category of parameters, the random samples are uniformly distributed via:

$$\theta_{i,2,t,init} = \frac{l_i + rand \cdot (u_i - l_i)}{u_i} \tag{12}$$

For the division of parameters into categories, we refer to Table 2, in which the category of the parameter is provided by stating whether transformation is linear or logarithmic.

Model simulation and parameterisation

The HDL and VLDL sub-models were implemented in Matlab (7.10, The MathWorks, Natick, Massachusetts). To limit computation time, calculation of conversion between model and *in silico* profile (Text S3), boundary components (equations (6) and (7)), scaling factors, geometrical lipoprotein properties and HDL production functions were calculated before simulation. Note that the VLDL production function could not be calculated before simulation, as it depends on the estimated parameter D. All values were expressed as (transformation) matrices and vectors, allowing calculation of fluxes to be performed by matrix manipulations.

The simulation of the lipoprotein sub-models was performed with Matlab function *ode15s*. To minimize simulation time, the VLDL sub-model was simulated first, and the HDL sub-model was simulated with the steady state value of the VLDL phospholipid release as an input. Both models were simulated for 1000 hours to allow a steady state to be reached. Following simulation, the concentrations in the model are scaled (Text S3) and *in silico* profiles and total fluxes due to a process are calculated.

For parameterisation of the model for the wild-type mouse, 1000 parameter sets were randomly drawn from a uniform distribution between the parameter boundaries (see equations 11 and 12). With each of these parameter sets (θ_i) as the initial value, the optimization in equation 3 was performed with the Matlab function *lsqnonlin* (Optimization toolbox, version 5.0). 'MaxFunEvals' was set to a value of 10000. Parameters were bounded to the values shown in Table 2.

(Local) optimization results showed a widely varying range of parameter values and final cost function values.

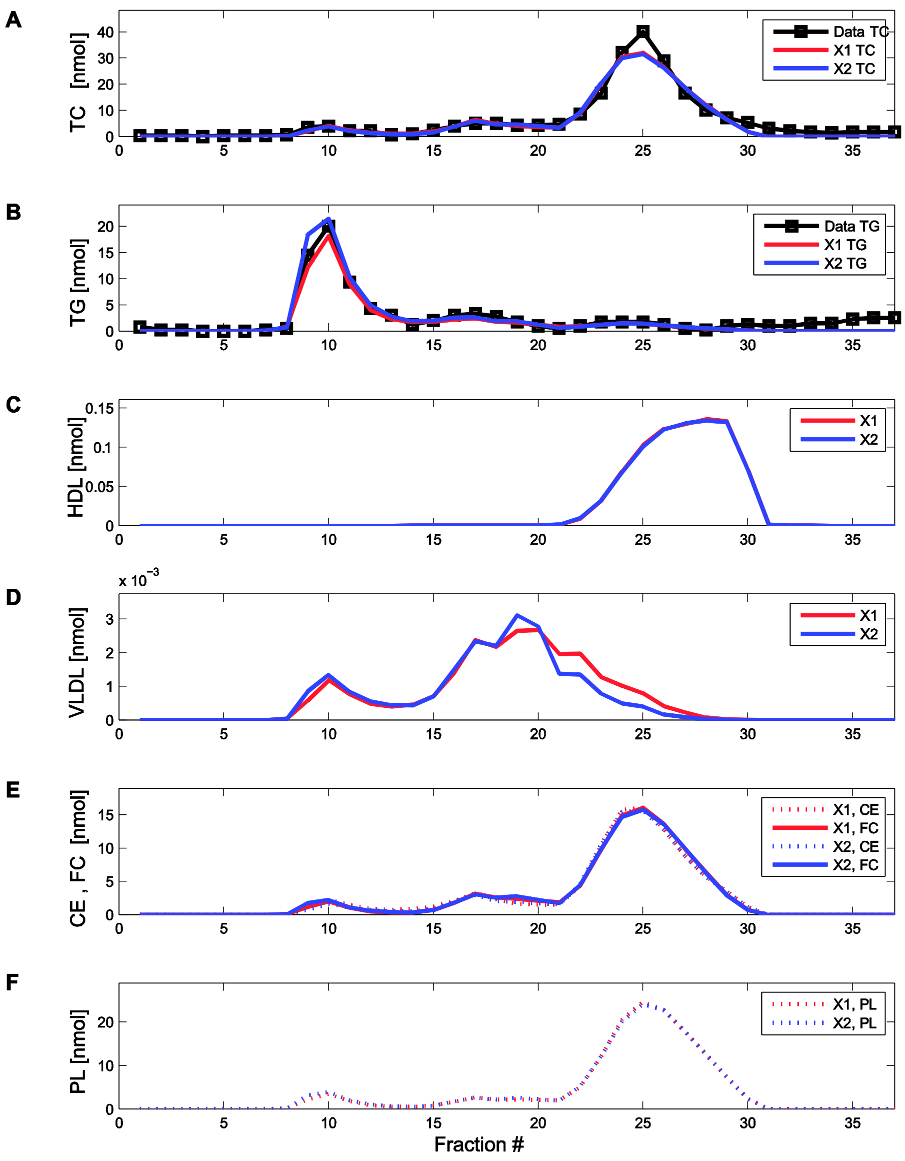

Figure 1 - FPLC profiles of all included lipid components and particles. FPLC profiles of all whole particles and lipid components in both found optima, as Figure 3 (Main Text) (X1 is shown in red, X2 is shown in blue). A. **Experimental and simulated FPLC profiles for cholesterol .** B. **Experimental and simulated profiles for TG.** C. **Simulated profiles for HDL particles.** D. **Simulated profile for VLDL particles.** E. **Simulated profiles for CE and FC.** F. **Simulated profiles for PL.**

Model acceptability

Following optimization, the *in silico* profiles of all 1000 resultant parameter sets (θ_{opt}) were evaluated. No measure of measurement uncertainty is known. The accepted parameter sets were selected based on characteristics of the profile.

Untreated mouse profiles were evaluated based on five measures:

- A lower cost function than the sum of squared errors of the best fit of the profile with constant values.
- A nascent VLDL diameter 10 % above VLDL peak size 41.3 nm and 5 % under VLDL grid maximum diameter 113.4 nm. A nascent VLDL diameter of 41.3 nm may result in a good fit of the VLDL peak, but is lower than the range of values found in literature ([1] - [2]). A nascent VLDL diameter of close to the grid maximum results in loss of the log-normal distribution of the nascent VLDL production, as the tail is severely truncated. While the explicit scaling of the VLDL production function by measured VLDL-TG precludes this to affect the VLDL-TG production, the shape of the production is unphysiological.
- A triglyceride peak in fraction number 10, i.e. the correct location of the VLDL peak in the TG profile. Evaluated by determining if fraction 10 is a peak in comparison to its neighbours.
- A cholesterol peak in fraction number 25, i.e. the correct location of the HDL peak in the C profile.
- The presence of the LDL peak and the shape of the VLDL peak. This final constraint was evaluated manually. Following the application of the first four constraints, 6 parameter sets were found. In four of these the LDL peak was partially or completely missing or the VLDL peak was a peak in comparison to its direct neighbours, but was not a clear peak in the full profile. These parameter sets were rejected.

Finally, two parameter sets were selected which fulfil all criteria. They will be referred to further as "X1" and "X2" and the values of the parameters can be found in Table 2. The *in silico* FPLC profiles simulated with each of these parameter sets are shown in Figure 1.

Table 2 –

Basal lipoprotein metabolism model parameter values and transformations

The two optimal solutions in this table represent the two originally found parameter sets, and therefore on example of each good fit. For HDL, it is important to remember that the HDL parameters are highly correlated and, in this table, unscaled. Kinetic parameters, denoted by *log* in the "T" (transformation) column, have an assumed logarithmic distribution and are transformed by taking the (natural) logarithm. Geometrical parameters, denoted by *lin*, have an assumed linear distribution and are transformed by dividing them by the upper bound. $scale_B$ is a derived model constant. $ratio_{A,1}$ - for k=1, $\mu_C = 24$ and $\sigma_C = 2$, for k=2, $\mu_C = 136.5$ and $\sigma_C = 14$. The lower (L) and upper(U) bounds of the parameters[2] are provided. These are also used for the initial sampling.

Parameter		X_1	X_2	L	U
(HDL)					
$scale_A$ *	$(\frac{\mu mol}{h \cdot kg})$	4.1466	0.7104	7.86 E-4	7.86 E8
$ratio_{A,1}$	$-$	0.9852	0.9625	0	1
c_{chol} *	$(\frac{kg}{\mu mol \cdot nm})$	102.5078	19.2178	8 E-5	5 E4
c_{selA} *	$(\frac{1}{h \cdot nm})$	1.7028	0.2644	1.3 E-6	2.5 E5
c_{lip}	(h^{-1})	1.4674	1.3580	4 E-3	2.5 E6
n	$-$	4.4379	6.1707	1	100
c_{trig} *	$(\frac{kg}{\mu mol})$	7.7397	1.6472	2 E-4	1.3 E5
$c_{uptakeA}$ *	$(\frac{nm}{h})$	233.9602	40.2213	3.2 E-2	4 E7
(VLDL)					
s_{min}	(nm^2)	$6.3075 \cdot 10^3$	$6.2882 \cdot 10^3$	0	S_{max}
c_{LPL}	$(\frac{1}{h \cdot nm^2}$	1.7514	0.5611	4 E-3	2.5 E6
$c_{uptakeB}$	(h^{-1})	0.0804	0.0106	4 E-3	2.5 E6
c_{selB}	$(\frac{1}{h \cdot nm})$	0.0115	0.0014	2.7 E-8	1.6 E3
μ_{upt}	(nm^2)	$4.0825 \cdot 10^3$	$4.8047 \cdot 10^3$	0	S_{max}
σ_{upt}	(nm^2)	614.5274	$1.1696 \cdot 10^3$	0	$S_{max}/2$
A_{upt}	$(\frac{nm}{h})$	$3.4609 \cdot 10^3$	$6.8145 \cdot 10^3$	1.6 E-3	$S_{max}/2 * 10^6$
D	(nm)	69.7391	56.0688	41.3	113.4036
$scale_B$	$(\frac{\mu mol}{h \cdot kg})$	$2 \cdot 10^{-3}$	$4 \cdot 10^{-3}$		

* These parameters are multiplied with a scaling factor ($\varphi_{experimental}$ / φ_{model}) to scale the model from a selective uptake of CE of 288.26 μmol/kg/ hour (X_1) or 44.56 μmol/kg/ hour (X_2) to scale the model to a selective uptake of CE of 5 μmol / kg / hour(=0.12 mmol/kg/day, [4]).

Parameter		T	$X_{1,t}$	$X_{2,t}$
(HDL)				
$scale_A$ *	$(\frac{\mu mol}{h \cdot kg})$	log	1.4223	-0.3419
$ratio_{A,1}$	$-$	lin	0.9852	0.9625
c_{chol} *	$(\frac{kg}{\mu mol \cdot nm})$	log	4.6299	2.9558
c_{selA} *	$(\frac{1}{h \cdot nm})$	log	0.5323	-1.3305
c_{lip}	(h^{-1})	log	0.3835	0.3060
n	$-$	log	1.4902	1.8198
c_{trig} *	$(\frac{kg}{\mu mol})$	log	2.0464	0.4991
$c_{uptakeA}$ *	$(\frac{nm}{h})$	log	5.4552	3.6944
(VLDL)				
s_{min}	(nm^2)	lin	0.1561	0.1556
c_{LPL}	$(\frac{1}{h \cdot nm^2}$	log	0.5604	0.5779
$c_{uptakeB}$	(h^{-1})	log	-2.5214	-4.5473
c_{selB}	$(\frac{1}{h \cdot nm})$	log	-4.4668	-6.6017
μ_{upt}	(nm^2)	lin	0.1010	0.1189
σ_{upt}	(nm^2)	lin	0.0304	0.0579
A_{upt}	$(\frac{nm}{h})$	log	8.1493	8.8268
D	(nm)	lin	0.6150	0.4944
$scale_B$	$(\frac{\mu mol}{h \cdot kg})$			

References

[1] Grefhorst A, Elzinga BM, Voshol PJ, Plosch T, Kok T, et al. (2002) Stimulation of lipogenesis by pharmacological activation of the liver X receptor (lxr) leads to production of large, triglyceride-rich VLDL particles. J Biol Chem 277: 34182-34190.

[2] Grefhorst A, Hoekstra J, Derks TGJ, Ouwens DM, Baller JFW, et al. (2005) Acute hepatic steatosis in mice by blocking -oxidation does not reduce insulin sensitivity of very-low-density lipoprotein production. Am J Physiol Gastrointest Liver Physiol 289: G592-G598.

[3] Pratt SM, Chiu S, Espinal GM, Shibata NM, Wong H, et al. (2010) Mouse hepatic lipase alleles with variable effects on lipoprotein composition and size. J Lipid Res 51: 1035-1048.

[4] van de Pas NC (2011) A physiologically based kinetic model for the prediction of plasma cholesterol concentrations in mice and man. Ph.D. thesis, Wageningen University
