# Supplementary material for: A Computational Model for the Analysis of Lipoprotein Distributions in the Mouse: Translating FPLC Profiles to Lipoprotein Metabolism
Source: PLoS Comput Biol. 2014 May 1;10(5):e1003579. doi: 10.1371/journal.pcbi.1003579 (PMC4006703; doi:10.1371/journal.pcbi.1003579)
Supplement: Text S5 — Wild-type model analysis. This text describes the performed re-sampling of parameter sets, the profile likelihood, the comparison of parameters with values from literature and additional information on the knock-out phenotypes. (PDF) [file pcbi.1003579.s005.pdf]

Supporting information – Text S5: Wild-type model analysis

A computational model for the analysis of lipoprotein distributions in the mouse: Translating FPLC profiles to lipoprotein metabolism

F. L. P. Sips, C. A. Tiemann, M. H. Oosterveer, A. K. Groen, P. A. J. Hilbers, N. A. W. van Riel

Following parameter estimation and evaluation, two parameter sets were found to describe the data well (these will be referred to as sets X1 and X2). Inspection of the *in silico* FPLC profiles (e.g. Figure 1 in Text S4) reveals that in the VLDL and LDL size range, the profiles of X1 and X2 do not overlap. For the HDL size-range, however, the profiles are very similar.

Keeping this observation in mind, the parameter sets themselves (Table 2 in Text S4) only partly collaborate these observations. The VLDL sub-model parameters indeed show great differences, e.g. in the production diameter and particle number (a factor of two difference in # of produced particles) as well as catabolism. The lipoprotein shape-dependent parameters (which are linearly scaled), correspond fairly well between parameter sets. This indicates the general structure of VLDL remodelling is similar between the two parameter sets.

For further analysis of the two parameter sets, we performed re-sampling of the found optimal parameter sets and calculated a profile likelihood (similar to [9], starting from both initial sets), as well as error plots of VLDL sub-model parameters.

Parameter space and parameter identifiability

Re-sampling of the optimal parameter sets

For a general idea of the parameter space near the optimal parameter sets, we re-sampled around the optimal sets. This was done be creating parameter sets with between -10 % and 10% and between -20% and 20% uniformly distributed random noise around the optimal parameter sets. Each set, for both 10% and 20%, was re-sampled >140 times. A total of 731 re-samplings were performed. All re-sampled parameter sets were optimized as described in Text S4. Analysis of the results revealed that again two types of acceptable results (according to the criteria defined in Text S4) were found, which could be classified as "X1" or "X2". Furthermore initial sets derived from "X1" were sometimes re-optimized to "X2" and vice versa.

Profile likelihood

A basal profile likelihood was performed. The object of the profile likelihood for parameter x is to find optimized values of all other parameters for a range of values of x and investigate for which x an acceptable result is attained, i.e. to investigate the parameter identifiability of the parameters in the model (e.g. [9]).

The profile of parameter x is initialized with the optimal set θ_{opt} (either X1 or X2). The parameter x is then fixed at values uniformly spanning the transformed parameter space between its optimized value and both parameter boundaries, after which a re-optimization takes place. For each new optimization the initial values of the free parameters (all but x) are chosen as the optimized parameter values of the previous optimization. This procedure is performed initially for every parameter in 29 steps (for a total of 30 sets) progressing from the optimized set to the upper and lower parameter bounds. This procedure was performed for all 16 parameters. If the resulting cost function profile is flat, i.e. the cost function has similar values for a large range of parameter values, the parameter cannot be uniquely identified [9].

Five kinetic parameters of the HDL sub-model are found to have an almost entirely flat profile. Identifiability of HDL parameters will therefore be assessed below. The proportion of HDL produced as the smaller type of nascent HDL is preferably equal to one. This indicates nearly all model HDL is produced as small HDL.

The profiles of VLDL sub-model parameters are in some cases (e.g. c_{uptB}, c_{LPL}, n) reasonably flat or only constrained in one direction. Superimposition of acceptability of these optimized parameter sets in terms of the position of HDL and VLDL peaks (as defined in Text S4) further constrains the parameters so that most VLDL sub-model parameters can be identified.

Parameter c_{selB} shows an interesting behaviour as the two parameter sets produce different profiles, with the parameter being identifiable in one optimum (X1) and unidentifiable in the other (X2). The analysis of D is shown in Figure 1.

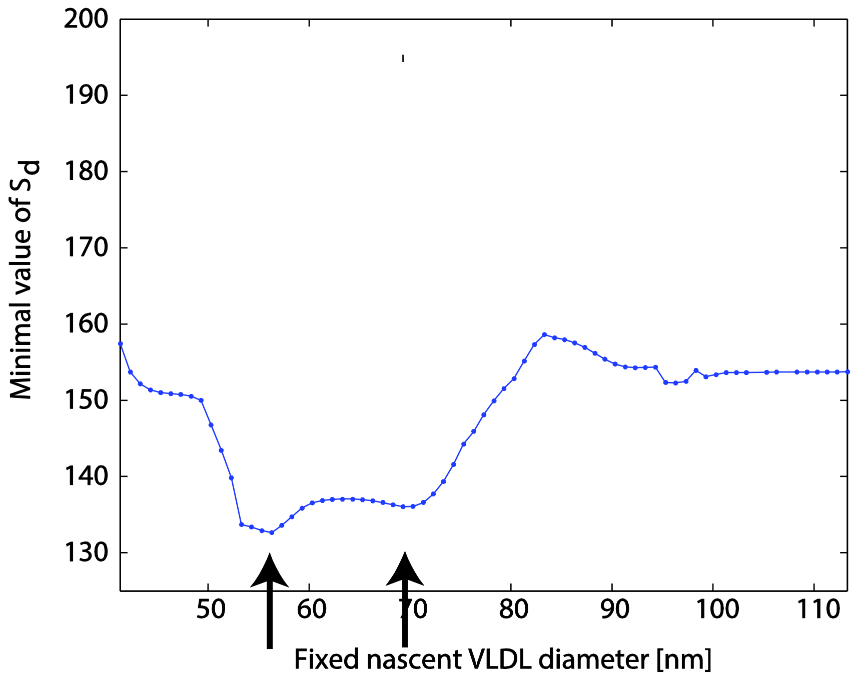

Figure 1. Profile likelihood.

Profile likelihood of parameter *D*. In this profile, fixed steps of 1 nm were taken. Two failed optimizations, with Sd > 1e10, were excluded. The two optima (X1 and X2) are located approximately at the arrows, with diameters of 69.74 and 56.07 nm respectively.

Conclusion

From these analyses a general conclusion about X1 and X2 can be drawn. From the crossing over from X1 to X2 both in the resampling and the profile of D, we conclude that most likely the two found optima, despite being found only once in the sampling, appear to be the only two local optima of the VLDL sub-model which produce an acceptable (Text S4) fit to the data.

The HDL sub-model

From the above analyses, it is apparent that 5 HDL sub-model parameters are unidentifiable and have a "flat" profile. In Figure 2 for the ratios of each parameters to parameter $scale_A$ has been plotted for all parameter sets of the two obtained (flat) profiles. This result indicates that the ratio between these five parameters is constant over all optimized sets. While the individual parameters are unidentifiable (due to the absence of absolute data on lipid fluxes in the HDL sub-model), the relation between the parameters is constrained. Without further information, however, HDL sub-model parameters and fluxes are known relative to each other.

The HDL lipolysis parameters do not scale with the remaining HDL parameters, indicating that lipolysis does not play an important role in the wild-type HDL sub-model.

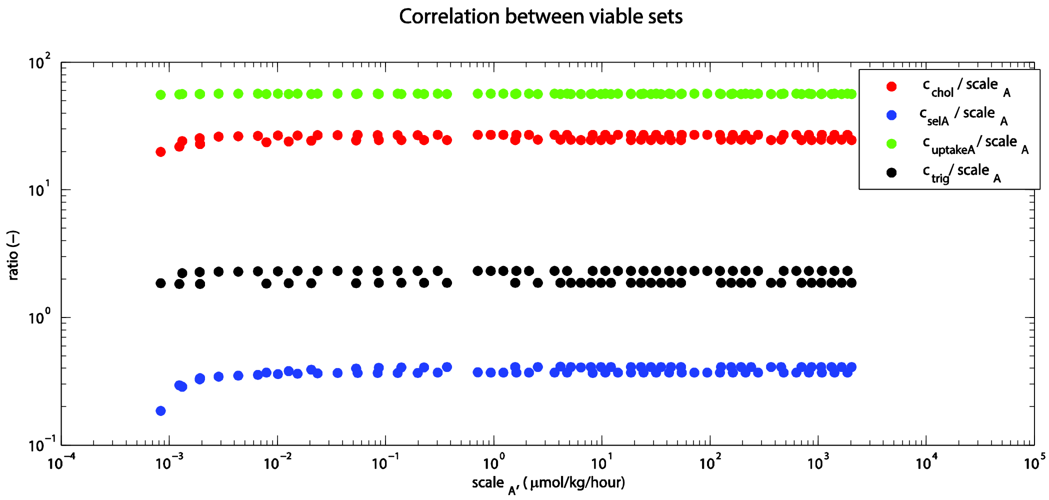

Figure 2 - Relative values of the HDL sub-model parameters, calculated for the profiles of c_{chol}**.**

The constant values show that apart from minor differences between X1 and X2 all HDL parameter sets are very similar. Presumably, the minor differences between the tails of the VLDL sub-model (Figure 1 in Text S4) cause a slightly different optimum of the HDL sub-model parameters. We note that both the c_{chol} **and** c_{trig} **parameters are multiplied by the amount of phospholipids released from VLDL. Because the difference is minor (20.5775 vs. 17.9554** μ**mol / kg / hour) the similarity between the datasets is already clearly seen in this uncorrected plot.**

HDL sub-model scaling

As concluded above in the analysis of the (wild-type) model, without further information on the magnitude of fluxes in the HDL sub-model, HDL sub-model results are known only relative to each other. For quantitative comparison of the VLDL and HDL sub-models, an absolute value of fluxes is necessary. For the wild-type mouse basal fluxes are available and scaling with a flux as known from literature can be performed to calculate absolute fluxes.

To scale the model, we take the known value of cholesterol uptake in the liver via selective uptake from HDL particles ([2], 5 μmol/kg/hour) and use this value to scale the model as shown in equation 1.

$$\theta_{s,r} = \frac{SelUp_d}{SelUp_{cm}} \cdot \theta_r \tag{1}$$

$SelUp_d$ is the literature value of the selective uptake flux, $SelUp_{cm}$ is the selective uptake flux in the model before parameter scaling, and θ_r are the 5 (proportional) parameters in the HDL sub-model, which will be scaled to $\theta_{s,r}$.

The VLDL sub-model

For the VLDL sub-model, the profile likelihood demonstrates a clear distinction between the two local optima. Differences include (a) the nascent VLDL diameter, (b) the VLDL/LDL selective uptake parameter c_{selB} and (c) the catabolism fluxes. X1 is an optimum with a higher initial diameter, in which parameter c_{selB} is more constrained than in X2.

This difference is demonstrated by the error plot in Figure 3. In these error plots, the value of the cost function S_d is calculated following the substitution in X1 or X2 of parameters D and c_{selB} *without* optimization. 125 x 125 values of each parameter, distributed uniformly between the bounds of D and log-uniformly between values of $log(c_{selB}) = -15$ and $log(c_{selB}) = 5$. This shows that c_{selB} is constrained in X1, but only partially constrained in X2. This is in agreement with the performed profile likelihood analysis.

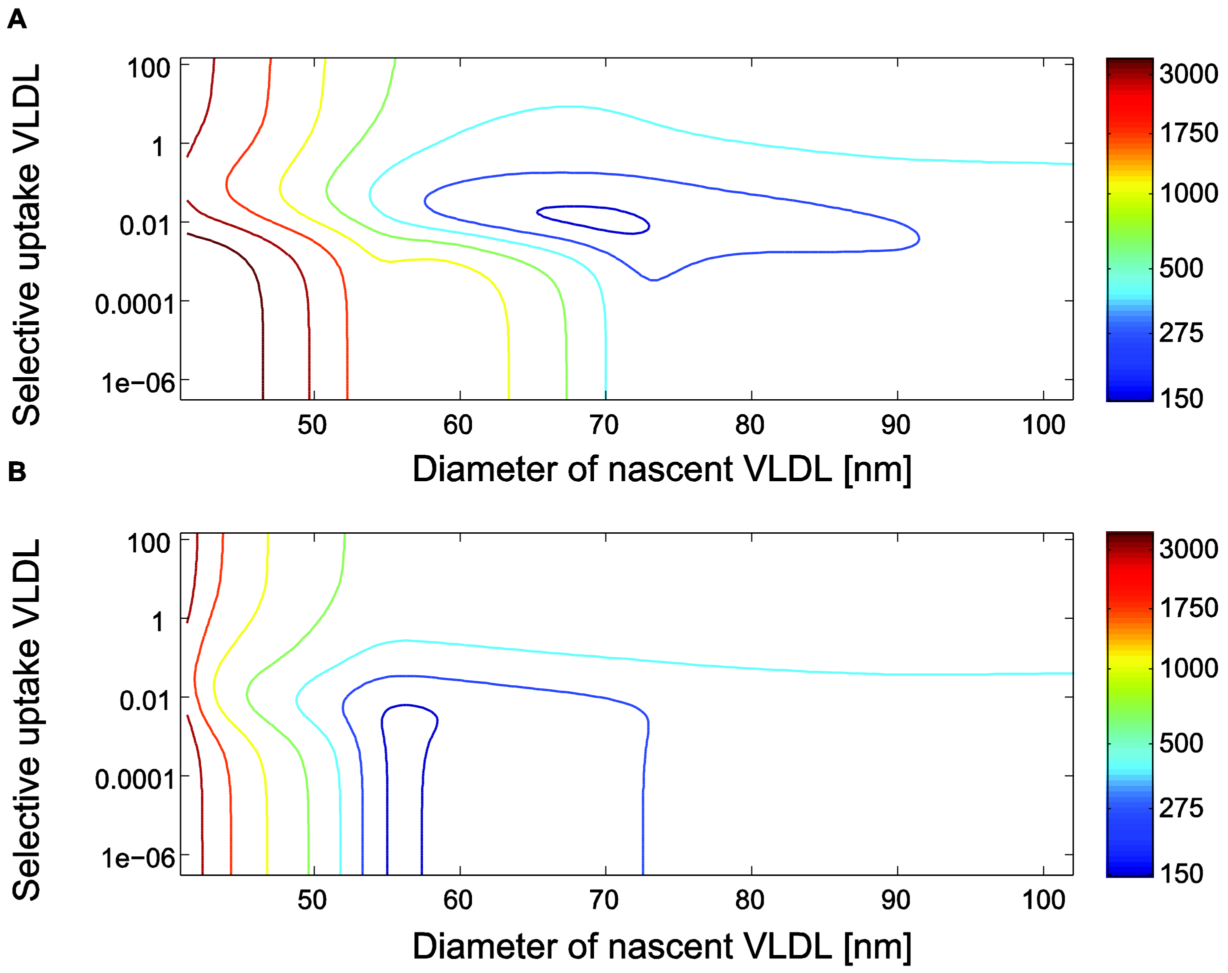

Figure 3 – Error plot of the c_{selB} and D parameters

Error plot for D vs. c_{selB}, calculated with a 125 x 125 grid of values. Error plots were generated by keeping all remaining parameters constant (to one of the optimal parameter sets) and fixing c_{selB} and D to values spanning a 125 by 125 grid over the pictured range. Contours show the value of S_d. The optima found are clearly visible . A. Parameter set X1 B. Parameter set X2

Comparison of parameter values to values from literature

Due to the phenomenological nature of the model equations in combination with practical difficulties in performing kinetic measurements in mice, comparison of parameters to literature values is only possible in a generic way. In the following section we evaluate the parameter values and, where possible, compare them with fractional catabolic rates found in literature. However, due constant change of lipoprotein kinetics following remodelling both *in vivo* and *in silico*, the following analysis is only a rough estimate of compatibility. In addition, the analysis of HDL sub-model parameters follows a crude scaling of the model.

HDL production rate *scaleA*

In order to compare the HDL parameters to literature, the 5 scalable parameters are first scaled such that the TC removal flux from HDL due to selective uptake equals 5 μmol/kg/hour.

$scaleA = 4.1466 \cdot \frac{5}{288.26} = 0.07$ μmol/kg/hour (X1) and 0.08 μmol/kg/hour (X2).

The production rate of spherical particles is unknown. According to Qin *et al* [8], the production rate of the main HDL apolipoprotein Apo A1 is 8.5 μg / g / hour. If we assume two ApoA1 per nascent HDL, and a molecular weight of $30.6 \cdot 10^3$ g / mol (UniProt, #Q00623) we can calculate an approximate experimental HDL production rate of $\frac{8.5 \cdot 10^3}{2 \cdot 30.6 \cdot 10^3} = 0.14$ μmol/kg/hour of nascent HDL production.

Nascent HDL shape parameter *ratioA,1*

The value of approximately one for the ratio of small to total nascent HDL can be interpreted as a high occurrence of nascent HDL with 2 ApoA-1 molecules, and a low occurrence of nascent HDL with 3-4 ApoA-1 molecules[1].

[1] please see Text S2 for the full description of nascent HDL

Cholesterol and triglyceride accumulation parameters c_{chol} and c_{trig}

While comparison of these values with literature is difficult, the fluxes through these pathways partly depend on the accuracy of the phospholipid transfer rate assumption. In Figure 4, the phospholipids as released from VLDL are compared with the phospholipids necessary for the predicted HDL growth (if the HDL sub-model is scaled as described). The results suggest that the assumptions of HDL surface remnant growth are reasonable.

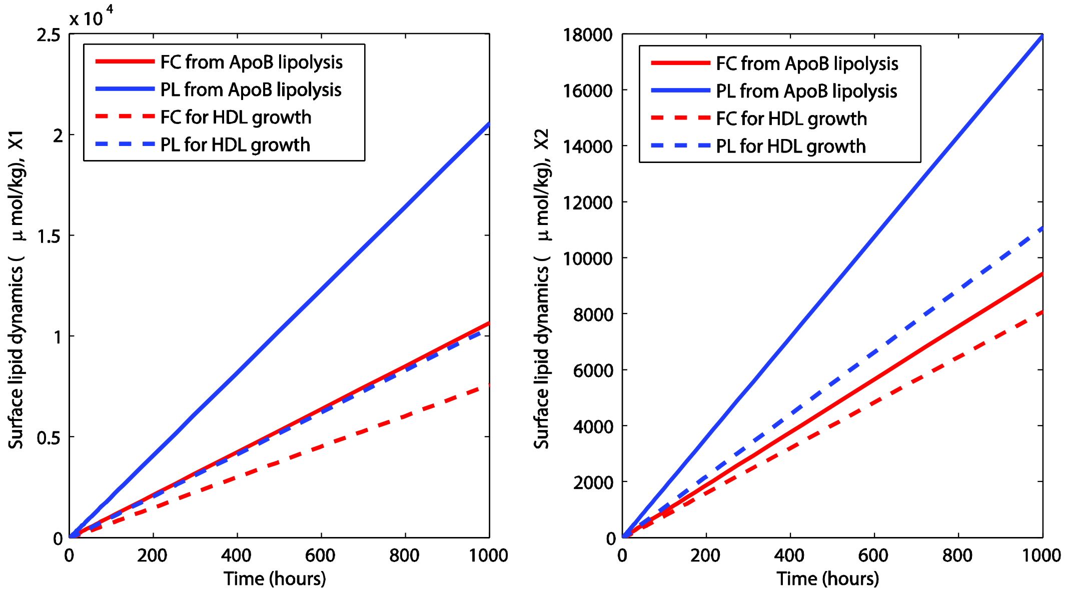

Figure 4: Comparison of surface remnants released *in silico* from VLDL due to lipolysis and taken up by (scaled) HDL. Left: parameter set X1, right: parameter set X2.

Selective uptake parameter c_{selA}

The fractional catabolic rate of (human) HDL-CE in mice was found by Brodeur *et al* [1] to be approximately 0.18 pools / hr. While in HDL metabolism the catabolism of the protein moiety does not necessarily impose catabolism of the lipid moiety, we can correct for the fractional catabolic rate of the protein moiety (0.077 / 0.078 pools/hr) to obtain an expected range of between 0.10 – 0.18 *pools / hr* uptake of HDL-CE.

In silico, as well as *in vivo*, the remodelling of lipoproteins depends on composition. As lipoproteins change the remodelling speed will also change. Lipoprotein CE FCR is a measure of selective uptake

lumped over the complete distribution of the lipoprotein class and integrated over a time period. Comparison of model parameters with global, lumped measures is therefore difficult. However here (and for the other parameters of lipolysis and selective uptake) we have computed the derivative of the loss of lipid – which approaches the FCR - for a typical lipoprotein. For HDL selective uptake, we first scale the selective uptake parameter $c_{selA} = 1.7028 \cdot \frac{5}{288.26} = 0.03 \frac{1}{nm \cdot hr}$ (X1). Assuming an HDL diameter of 11.3 nm and a TC to TG molar ratio of approximately 20:1 (as in the wild-type FPLC profile) this corresponds to an approximated FCR of $0.0295 \cdot 11.3 \cdot \frac{20}{21} = 0.32\, pools/hr$.

The *in silico* value is slightly higher than the *in vivo* measures, however we note that the *in silico* FCR will decrease as the lipoproteins become smaller. Also, the experimental measures are of human HDL3, which is smaller (~ 8 nm) [8] has a cholesterol to TG ratio of 45:9.5 [8] and therefore an *in silico* FCR of 0.19 *pools/hr*, but may also have a different affinity for SR-B1.

HDL uptake parameter $c_{uptakeA}$

The fractional catabolic rate of the protein moiety of (human) HDL was found in [1] to range between 0.07 and 0.1 pools / hr.

In silico, the catabolic rate of HDL is determined by the parameter $c_{uptakeA}$. This parameter, when scaled, has a value of $c_{uptakeA} = 233.96 \cdot \frac{5}{288.26} = 4.06\, nm/hr$ (X1) or 4.51 nm/hr (X2). For a typical HDL diameter of 11.3 nm, this is thus a fractional catabolic rate of 0.36 -0.40 pools / hr. While this is higher than the experimental value, we again note that due to the flexibility of the ApoA content of an HDL particle the fractional catabolic rate of the HDL protein moiety is not expected to be identical to the fractional catabolic rate of spherical HDL.

Nasent VLDL diameter *D*

Many estimates of nascent VLDL diameter are available in literature. We compare the optima of 69.7 and 56.1 nm with comparable results published in [4] and [5]. The values measured in these experiments for nascent VLDL-diameter are 94 ± 12 nm and 59.3 ± 3.6 nm , respectively. We conclude the parameter values are close to the expected range.

Size-dependent lipolysis parameters c_{LPL} and s_{min}

In vivo, the value of lipolysis parameter c_{LPL} is high and lipolysis of the secreted VLDL is practically immediate. This corresponds to the short half-life of VLDL-TG in plasma.

In silico, c_{LPL} = 0.56 – 1.75 $\frac{1}{h \cdot nm^2}$. For a VLDL diameter of e.g. 45 nm, this corresponds to a FCR of 34.6 – 108 pools/hr. We note that the FCR of larger particles may be much higher, e.g. the FCR of particles with a larger diameter of e.g. 60 nm is $2.8 \cdot 10^3 - 8.8 \cdot 10^3$ pools / hr. However in this case the difference between model parameters and FCR is clearly a factor as the rate of lipolysis will immediately decrease as the TG content of the particle decreases. The minimal surface area s_{min} corresponds to a minimal diameter of 44.8 nm, meaning the function will only affect the conversion of large particles.

Non-size dependent lipolysis parameters *n* and c_{lip}

Scaling c_{lip} – not within the HDL sub-model but to the value of a value of *n* as described in Text S2 – we retain a value of 2.5-3.5 per hr. Including the approximate composition of HDL or LDL (we assume that in LDL, TG:PL is about 2:1, in HDL it is about 5:1 [8]:

For LDL: $c_{lip,scaled} \cdot \left(\frac{\mathrm{TG}(i)}{\mathrm{TG}(i) + \mathrm{PL}(i,j)} \right)^n = 2.5 \cdot \left(\frac{2}{3}\right)^{4.44} = 0.42\ hr^{-1}$ for X1, $0.29\ hr^{-1}$ for X2

For HDL: $c_{lip,scaled} \cdot \left(\frac{\mathrm{TG}(i)}{\mathrm{TG}(i) + \mathrm{PL}(i,j)} \right)^n = 2.5 \cdot \left(\frac{1}{6}\right)^{4.44} = 9 \cdot 10^{-4}\ hr^{-1}$ for X1 to $5.5 \cdot 10^{-5}\ hr^{-1}$ for X2. More information on the magnitude of VLDL lipolysis is found below, please see "VLDL-TG production".

Size-dependent uptake parameters A_{upt}, μ_{upt}, and σ_{upt}

μ_{upt} = 4.08 – 4.8 10^3 translates to a diameter of 36.0 – 39.1 nm. This is indeed in the range between VLDL and LDL and therefore complies with the assumptions.

The maximal uptake A_{upt}/σ_{upt} = 5.6-5.8, is in the expected value for ApoE mediated uptake - e.g. 8 - 4.1 pools/h in [3].

Non size-dependent uptake parameter $c_{uptakeB}$

The fractional catabolic rate of the protein moiety of (human) LDL was found in [1] to range from an initial value of up to 4.5 pools/hr to a value of 0.1 pools / hr.

In silico, we find 0.08 – 0.01 pools / hr. This is lower than the experimental value of we consider only the size-independent equation. *In vivo* a combination of both mechanisms could be in play, leading to a higher FCR.

VLDL selective uptake parameter c_{selB}

In [1], injected LDL were found to have a 31 % decrease in CE to protein ratio following three hours of circulation. A decrease of 31 % per 3 hours corresponds approximately with a decrease of 12 % per hour. As we have concluded the parameter c_{selB} is not constrained in parameter set X2, we will only compare to the value in X1.
The value of c_{selB} is approximately 0.0115 $\frac{1}{hr \cdot nm}$. Assuming a cholesterol to TG ratio of approximately 2:1 in LDL (wild-type FPLC profile) this thus corresponds with a decrease of 0.0115 $\cdot$ 22.6 $\cdot \frac{2}{3}$ = 0.17 *pools/hr* for typical LDL.

VLDL – TG production

To establish whether the model can derive VLDL-TG production from Triton WR-1339 estimates, simulations of the model from steady state were performed. We note that while the size-dependent lipolysis equation (TextS2, equation (32)) is presumed to represent only LPL lipolysis, both lipolysis equations have been lumped and therefore the second lipolysis equation (Text S2, equation (33)) could contribute to peripheral VLDL lipolysis. Simulations in which either one of the equations is inhibited show that, while the relative contribution of the second lipolysis equation is small, it is large enough to prevent the expected linear increase in plasma VLDL-TG. Inhibition of both lipolysis functions leads to such a linear increase. The resulting difference between accumulation in case of no VLDL metabolism and accumulation in case of no VLDL lipolysis seems to concur with the experimentally observed decrease of the fractional catabolic rate of VLDL by 90 % [6] following

Triton administration. Whereas model implementation in which the Triton WR-1339 induced VLDL-TG production concurs with this flux is possible, the difference between the two fluxes is relatively small and it would be expected to lead to similar results.

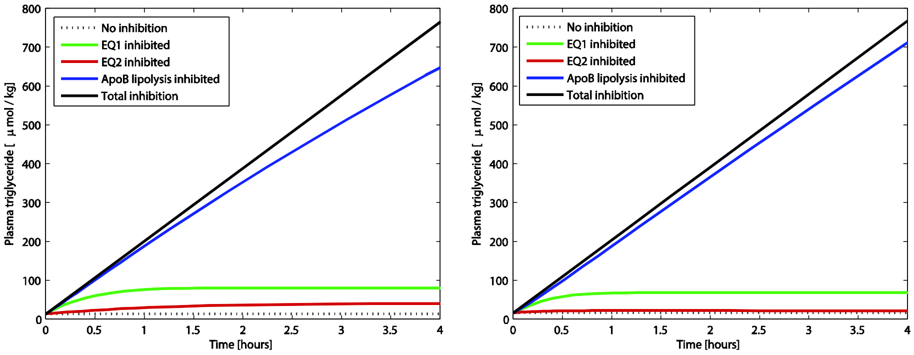

Figure 5: Simulations of plasma TG accumulation in 4 hours during inhibition of lipolysis or all VLDL metabolism. Left: Parameter set X1, Right: X2. Simulations were initialized at steady state. EQ1 is the size-dependent lipolysis equation, while EQ2 is the non size-dependent lipolysis equation.

Additional analysis of knock-out phenotypes

C concentrations in *in silico* knock-out phenotypes

In the SR-B1 knock-out mice (c_{selA} at 1e-10 % of original parameter value) the plasma cholesterol concentration rises by 73 % (X1) and 65 % (X2) of the wild-type value of the plasma cholesterol concentration. The HDL-TC concentration rises by 93 and 81 % respectively.

In the PLTP knock-out mouse, the plasma cholesterol concentration is decreased by 55 % ([2], Male wild-type vs. male PLTP KO, on a chow diet). In the model, choosing a value of c_{chol} which is 30 % of the wild-type value corresponds to a 55 % reduction of plasma cholesterol, for both X1 and X2.

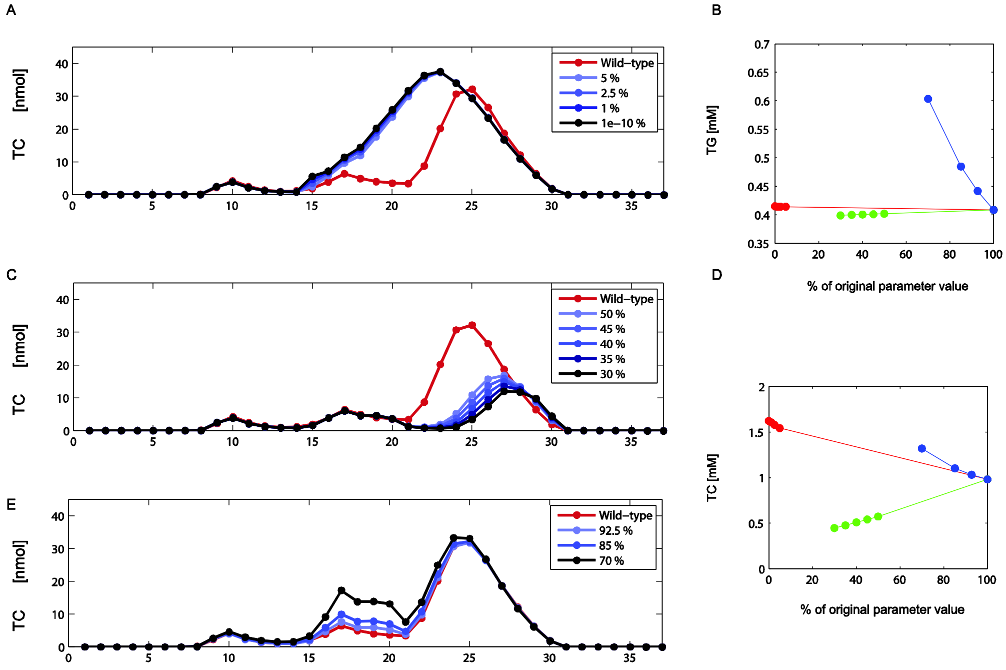

Figure 6. ***In silico*** **profiles of transgenic mice.**

As Figure 4 (Main text). Now pictured is parameter set X2. A. *in silico* SR-B1 knock-out mice. B. Plasma TG for knock-out phenotypes. SR-B1 knock-out is shown in red, PLTP knock-out is shown in green, and LDLr knock-out is shown in blue. C. PLTP deficiency. D. Plasma TC, as C. E. LDLr knock-out. Qualitatively, the knock-out profiles of X1 and X2 are similar. Quantitatively, small differences between the sets X1 and X2 are observed in the SR-B1 and PLTP knock-out simulations (e.g. the difference in plasma TC or TG). For the LDLr knock-out mouse, the lative rise in plasma cholesterol for reduction of the parameters is greater in X2. E.g. the maximal reduction of 30 % is this figure produces a quantitative response similar to the maximal reduction in Figure 4 of the main text, of 60 %.

References

[1] Brodeur MR, Luangrath V, Bourret G, Falstrault L, Brissette L (2005) Physiological importance of SR-BI in the in vivo metabolism of human HDL and LDL in male and female mice. J Lipid Res 46: 687-696.

[2] Cao G, Beyer TP, Yang XP, Schmidt RJ, Zhang Y, et al. (2002) Phospholipid transfer protein is regulated by liver X receptors in vivo. J Biol Chem 277: 39561-39565.

[3] Ebara T, Conde K, Kako Y, Liu Y, Xu Y, et al. (2000) Delayed catabolism of apoB-48 lipoproteins due to decreased heparan sulfate proteoglycan production in diabetic mice. J Clin Invest 105: 1807-1818.

[4] Grefhorst A, Elzinga BM, Voshol PJ, Plosch T, Kok T, et al. (2002) Stimulation of lipogenesis by pharmacological activation of the liver X receptor (lxr) leads to production of large, triglyceride-rich VLDL particles. J Biol Chem 277: 34182-34190.

[5] Grefhorst A, Hoekstra J, Derks TGJ, Ouwens DM, Baller JFW, et al. (2005) Acute hepatic steatosis in mice by blocking -oxidation does not reduce insulin sensitivity of very-low-density lipoprotein production. Am J Physiol Gastrointest Liver Physiol 289: G592-G598.

[6] John S Millar MGMDJR Debra A Cromley, Billheimer JT (2005) Determining hepatic triglyceride production in mice: comparison of poloxamer 407 with triton wr-1339. J Lipid Res 46: 2023-2028.

[7] van de Pas NC (2011) A physiologically based kinetic model for the prediction of plasma cholesterol concentrations in mice and man. Ph.D. thesis, Wageningen University.

[8] Qin S, Kawano K, Bruce C, Lin M, Bisgaier C, et al. (2000) Phospholipid transfer protein gene knock-out mice have low high density lipoprotein levels, due to hypercatabolism, and accumulate apoA-IV-rich lamellar lipoproteins. J Lipid Res 41: 269-276.

[9] A. Raue, C. Kreutz, T. Maiwald, J. Bachmann, M. Schilling, U. Klingmüller, and J. Timmer. (2009) Structural and practical identifiability analysis of patially observed dynamical models by exploiting

the profile likelihood. *Bioinformatics*, 25(15):1923–1929

[10] Shen BW, Scanu AM, Kézdy FJ (1977) Structure of human serum lipoproteins inferred from compositional analysis. Proc Natl Acad Sci U S A 74: 837-841.
