# Supplementary material for: A Computational Model for the Analysis of Lipoprotein Distributions in the Mouse: Translating FPLC Profiles to Lipoprotein Metabolism
Source: PLoS Comput Biol. 2014 May 1;10(5):e1003579. doi: 10.1371/journal.pcbi.1003579 (PMC4006703; doi:10.1371/journal.pcbi.1003579)
Supplement: Text S6 — LXR model development. Derivation and equations of the adaptations and extensions included in the computational models of the LXR activated mouse. (PDF) [file pcbi.1003579.s006.pdf]

Supporting information – Text S6: LXR model development

A computational model for the analysis of lipoprotein distributions in the mouse: Translating FPLC profiles to lipoprotein metabolism

F. L. P. Sips, C. A. Tiemann, M. H. Oosterveer, A. K. Groen, P. A. J. Hilbers, N. A. W. van Riel

This text describes the application of the model to the profiles of mice treated with T0901317. This is divided into three steps:

1. Adapting the model by changing model constants to describe the experimental data. The result is examined by evaluating the profiles of mice treated with T0901317 for 14 days.
2. Extending the HDL sub-model with three additional mechanisms. The result is - again - examined by evaluating the profiles of mice treated with T0901317 for 14 days.
3. Applying the extended models to profiles obtained from mice treated with T0901317 for 1, 2, 4, 7 and 21 days. In this step, VLDL production for days 1 and 2 is elaborated.

1. Model adaptation for LXR activation

The simulation of LXR activated mice requires several changes to model variables. Experimental data of plasma volume and body weight[1] are incorporated into model variables. Also, the measured VLDL-TG production shows an increase in the weeks following T0901317 treatment. The VLDL production function is changed accordingly.

However, because nascent VLDL produced following LXR activation is known to be of a larger size than nascent VLDL in untreated mice ([6], [3]) it does not suffice to increase the production rate. The lipid content of nascent VLDL is adapted to these observations.

In Grefhorst *et al.* (2002)[3], the increase in nascent VLDL size following four days of treatment with T0901317 is concluded to be completely explained by an increase of the triglyceride content. In the model, we will therefore assume that the VLDL production distribution is changed so that the triglyceride content of nascent VLDL increases by the ratio of treated VLDL-TG production to control VLDL-TG production. For this calculation the wild-type TG content of nascent VLDL is fixed at either the value in X1 or the value in X2.

$$E[\#\mathrm{TG}]_d = \frac{\mathrm{VLDL-TG}_d}{\mathrm{VLDL-TG}_{control}} \cdot E[\#\mathrm{TG}]_{control} \tag{1}$$

$$Var[\#\mathrm{TG}]_d = \left(\frac{\mathrm{VLDL-TG}_d}{\mathrm{VLDL-TG}_{control}} \cdot SD[\#\mathrm{TG}]_{control} \right)^2 \tag{2}$$

The parameters of the log-normal distribution of the VLDL production can be determined with these computed values. We note that following scaling of both the variance and mean, the value of the log-normal distribution parameter σ_{TG} does not change. As the difference in VLDL-TG production is captured by this increase in TG content, the value of the VLDL production variable $scale_B$ remains constant[2]. Also the diameter of nascent VLDL now depends on the diameter of VLDL in the control mouse - the diameter in the LXR activated model is therefore fixed, reducing the total number of model parameters from 16 to 15.

Following these changes, 50 initial parameter sets were generated based either on X1 (25) or X2

---

[1] Plasma volume for all treated mouse phenotypes is set at 0.98 mL, while bodyweight is fixed at 23.75 grams

[2] To ensure the correct VLDL-TG production in the model, even if some distribution truncation or distortion plays a role, the value of $scale_B$ is always recalculated.

(25), with the addition of uniformly distributed random noise of between -20 % and 20 % of the control profile parameter value. Optimisation was performed as described in Text S4. For the initial evaluation of the model's capability to describe the LXR activated data, only the dataset measured at 14 days was used. 50 optimized parameter sets were obtained. Analysis of the results (Figure 1) resulted in the rejection of the model due to the inability to describe the data qualitatively. The characteristic (see Main Text) second, large particle HDL peak was not described by the model. While in some cases a small secondary peak can be seen in Figure 1, these parameter sets were rejected because the magnitude of this second peak was insufficient and because the peak was found to result from LDL particles.

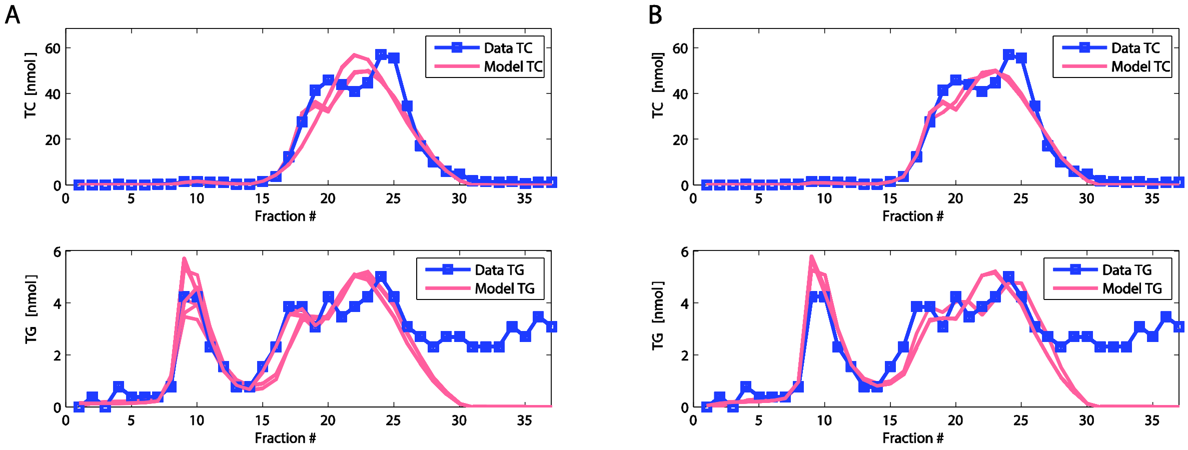

Figure 1 - *In silico* profiles generated by fitting the basal model to profiles from mice treated with T0901317 for 14 days

The experimental data (blue) and simulated profiles (red) of both the cholesterol (top) and triglyceride (bottom) profiles are shown. The results show that while the general size and composition of the HDL is well reproduced, the enlarged HDL does not appear. The small secondary peak seen in some local optima is an LDL peak and therefore does not fulfil the qualitative criteria wielded for these profiles. A. Diameter based on parameter set X1 B. Diameter based on parameter set X2

2. HDL sub-model extension for LXR activation

Adapting the wild-type model to the LXR activated state by applying changes to model constants based on experimental data was shown in the previous section to not produce an adequate description of the profiles obtained from treated mice. For further analysis of the profiles obtained from LXR agonist treated mice, the HDL sub-model was therefore be extended.

Underlying a phenotype change in lipoprotein metabolism may be an adaptation of particle production, remodelling or catabolism [2]. In this section, the proposed explanations will be described in more detail, and the equations will be introduced. In each extension, one equation in the HDL sub-model is elaborated.

Extension 1 is an addition to the cholesterol efflux equation (equation (41), Text S2), and is therefore a remodelling change. The altered protein content of the enlarged HDL will change their affinity for proteins such as those involved in peripheral cholesterol efflux (ATP binding cassettes A1 and G1, SR-B1 [9]). In particular, the Apo E which is known to be found on enlarged HDL could be the source of this higher affinity ([4]).

The equation associated with this adaptation assumes the altered affinity is a characteristic of enlarged HDL and is therefore modelled via an interaction that is only present for particles above a defined minimal surface area. The extended cholesterol uptake equation is given in equations 3 - 4 and includes 2 additional parameters, $s_{min,2}$ and c_{E1}.

$$r_{chol|i,j} = \left(c_{chol} \cdot y_{B,surfremn} \cdot \frac{D(i,j)}{4} + E1(s(i,j)) \right) \cdot \frac{1}{\Delta_D \mathrm{TC}(i,j)} \tag{3}$$

$$E1(s(i,j)) = \begin{cases} s(i,j) \geq s_{min,2} & (s(i,j) - s_{min,2}) \cdot c_{E1} \\ s(i,j) \leq s_{min,2} & 0 \end{cases} \tag{4}$$

In this equation, $s(i,j)$ is the surface area of the particle.

The second extension **Extension 2** for the observed phenotype change is based on a similar change in affinity between surface proteins and receptor proteins, in this case leading to an increased catabolism of lipoproteins. Apo E on HDL, as on VLDL, may interact with the LDL receptor and other receptors that interact with Apo E on triglyceride-rich lipoproteins [7], [1].

Therefore, the equation implemented for Apo E mediated uptake in the VLDL sub-model (Text S2, equation (38)) is applied again in the second extension of the HDL sub-model, and added to the equation for HDL catabolism (Text S2, equation (48)). The extended equation for HDL catabolism contains 3 additional parameters.

$$r_{upt|i,j} = c_{uptA} \cdot \frac{1}{D(i,j)} + c_{uptE2|i,j} \tag{5}$$

$$c_{uptE2|i,j} = \frac{A_{upt,E2}}{\sigma_{upt,E2}} e^{-\frac{\left(s(i,j)-\mu_{up,E2}\right)^2}{2\sigma_{upt,E2}^2}} \tag{6}$$

The final extension (**Extension 3**) of the HDL sub-model is based on observations made in *in vitro* experiments following treatment with T0901317 by Kurano *et al.* (2011) [5]. In the experiments performed by Kurano *et al. in vitro*, the observed HDL are of a larger initial size and different composition than the wild-type produced lipoproteins [5]. Following this observation, an additional production of lipoproteins in addition to the basal production is added to the HDL production equation (Text S2, equation (39)).

The large particle production is modelled in addition to basal production. As no further information on the composition of a large HDL production peak is available, the production function $prod_{A,E3}$ is modelled as a Gaussian function over the CE index *j*. For further analysis of this explanation, it is important to note that $scale_A$ and $scale_{E3}$ are now both inputs of the HDL grid. This extension contains 3 additional parameters.

$$u_{prod|i,j} = scale_A \cdot \sum_{k=1}^{k_{max}} ratio_{A,k} \cdot prod_{A,k}(i,j) + scale_{E3} \cdot prod_{A,E3}(i,j) \tag{7}$$

$$prod_{A,E3}(i,j) = \begin{cases} i > 1 & 0 \\ i = 1 & prod_{A,E3}(1,j) = \int_{j-0.5}^{j+0.5} f(j,\mu_{E3},\sigma_{E3})dj \end{cases} \tag{8}$$

Where $f(j,\mu_{E3},\sigma_{E3})$ is a normal distribution with parameters μ_{E3} and σ_{E3}.

It is important to note, however, that the equations above may have alternative mechanistical explanations. In the third extension of the model, for instance, the additional model input may be

the result of a remodelling step which results in a fast change of particle composition, leading to an observed input of particles. In each case, alternative, biologically plausible mechanisms leading to an indistinguishable equation may be available.

As described in the Main Text, all three extended models were fitted to the profiles of mice treated with T0901317 for 14 days (Main Text, Figure 6). 50 initial parameter sets were produced, consisting of the 15 parameters as determined for the control profile (25 x X1 and 25 x X2, with associated model constants for VLDL production) and random sampling of the newly introduced parameters. The extension parameters were sampled from between the bounds, as was previously described for the control profile (Text S4, for parameter category please see Text S7). Optimisation was performed as described in Text S4.

The parameter sets resulting from optimisation were evaluated manually. All three extended models were able to describe the data well both quantitatively and qualitatively. Parameters of representative fits of the 14 days treated profile are provided in Text S7. Parameter values and fluxes are also pictured in Text S8. The found accepted parameter sets will be used as a basis for the estimation of parameters in the third section of this text.

3. VLDL production extension for LXR activation

The accepted parameter sets from the previous section are now used as a basis for the estimation of parameters at the remaining time points. In more detail, the profiles of 7 and 21 days of treatment are fitted using the accepted 14 days parameter sets as initial values. The 4 days profile was fitted by using the results of the 7 days optimisation as a starting point. The initial fit of day 1 and day 2 data (created by a progression from day 4 parameter values) was found to inadequately describe the data, especially in terms of VLDL peak height. Following a release of several VLDL production assumptions (see below), the models were re-fitted by using the day 14 parameters as initial values[3]. Note that at not all time points VLDL-TG production is available; in these cases the value is (linearly) interpolated or extrapolated.

There may be several reasons for the models inability to correctly describe the data at day 1 and day 2 of treatment. First of all, during the first days of treatment the steady state assumption may not be applicable. However, the half-value time of Apo B containing lipoproteins is very small in mice [8].

In [3], the observations of VLDL composition change are performed after a treatment period of 4 days, whereas the data-points which the model is not found to describe were measured in the initial phase of treatment. An explanation we will explore here is therefore that the assumption of VLDL composition is not applicable in the first days of treatment. For the VLDL production on day 1 and 2 of treatment only, two parameters to be estimated were added to all models. These extend the model as described in equations 9-10.

$$[\mathrm{CE}] = \frac{pr1 \cdot [\mathrm{CE}]}{(pr1 \cdot [\mathrm{CE}]) + [\mathrm{TG}]} \tag{9}$$

The first equation perturbs the assumed distribution between CE and TG as described in the data (Text S2), resulting in a change of equations (23) and (24) in Text S2. In this equation, $pr1$ is a novel parameter.

$$D_{WT} = pr2 \cdot D_{WT} \tag{10}$$

The second adjustment is to provide more freedom in nascent VLDL size, by defining that the wild-type diameter is first multiplied with a parameter $pr2$, before the corresponding treated nascent VLDL diameter is computed via the first section of this text. D_{WT} is the wild-type VLDL

[3] For the additional parameters that will be described below, the default value of 1 - corresponding to no change from the initial assumption - was chosen in the initial set.

diameter. X1 and X2 each have a unique value of the D_{WT}.

Optimization is again performed via the procedure outlined in Text S4. The results were manually assessed. All accepted sets and resultant computed fluxes are pictured in Text S8. Notably, the VLDL production on day 1 and day 2 no longer has two optima, but instead is defined by the same curve.

Table 1: Additional production parameters

Bounds, initial values and type of parameters $pr1$ and $pr2$

Parameter	Initial value	Low bound	High bound	Type
pr1	1	10^{-3}	10^3	log
pr2	1	$\frac{43}{D_{WT}}$	$\frac{113}{D_{WT}}$	lin

Table 2 – Lipoprotein metabolism model extension equations

Equations for Extensions E1, E2 and E3. The extensions contain 2, 3 and 3 parameters. $s(i, j)$ **is the particle surface area in** nm^2, $s_{min,2}$ **is a parameter which describes the minimal surface for which the additional uptake takes place, and** c_{E1} **is a model constant.** $f(j, \mu_{E3}, \sigma_{E3})$ **is a normal distribution over *j* with parameters** μ_{E3} **and** σ_{E3}.

Extension 1	
$$r_{chol\|i,j} = \left(c_{chol} \cdot y_{B,surfremn} \cdot \frac{D(i,j)}{4} + E1(s(i,j)) \right) \cdot \frac{1}{\Delta_D \mathrm{TC}(i,j)}$$	(1)
$$E1(s(i,j)) = \begin{cases} s(i,j) \geq s_{min,2} & (s(i,j) - s_{min,2}) \cdot c_{E1} \\ s(i,j) \leq s_{min,2} & 0 \end{cases}$$	(2)
Extension 2	
$$r_{upt\|i,j} = c_{uptA} \cdot \frac{1}{D(i,j)} + c_{uptE2\|i,j}$$	(3)
$$c_{uptE2\|i,j} = \frac{A_{upt,E2}}{\sigma_{upt,E2}} e^{-\frac{\left(s(i,j) - \mu_{up,E2}\right)^2}{2\sigma^2_{upt,E2}}}$$	(4)
Extension 3	
$$u_{prod\|i,j} = scale_A \cdot \sum_{k=1}^{k_{max}} ratio_{A,k} \cdot prod_{A,k}(i,j) + scale_{E3} \cdot prod_{A,E3}(i,j)$$	(5)
$$prod_{A,E3}(i,j) = \begin{cases} i = 1 & prod_{A,E3}(1,j) = \int_{j-0.5}^{j+0.5} f(j, \mu_{E3}, \sigma_{E3}) dj \\ i > 1 & 0 \end{cases}$$	(6)

References

[1] Bencharif K, Hoareau L, Murumalla R, Tarnus E, Tallet F, et al. (2010) Effect of apoA-I on cholesterol release and apoE secretion in human mature adipocytes. Lipids Health Dis 9: 75.

[2] Borén J, Taskinen MR, Adiels M (2012) Kinetic studies to investigate lipoprotein metabolism. J Intern Med 271: 166-173.

[3] Grefhorst A, Elzinga BM, Voshol PJ, Plosch T, Kok T, et al. (2002) Stimulation of lipogenesis by pharmacological activation of the liver X receptor (lxr) leads to production of large, triglyceriderich VLDL particles. J Biol Chem 277: 34182-34190.

[4] Jiang XC, Beyer TP, Li Z, Liu J, Quan W, et al. (2003) Enlargement of high density lipoprotein in mice via liver X receptor activation requires apolipoprotein E and is abolished by cholesteryl ester transfer protein expression. J Biol Chem 278: 49072-49078.

[5] Kurano M, Iso-O N, Hara M, Ishizaka N, Moriya K, et al. (2011) LXR agonist increases apoe secretion from HepG2 spheroid, together with an increased production of VLDL and apoE-rich large HDL. Lipids Health Dis 10.

[6] Okazaki H, Goldstein JL, Brown MS, Liang G (2010) LXR-SREBP-1c-phospholipid transfer protein axis controls very low density lipoprotein (VLDL) particle size. J Biol Chem 285: 6801-6810.

[7] Rader DJ (2006) Molecular regulation of hdl metabolism and function: implications for novel therapies. J Clin Invest 116: 3090-3100.

[8] Rensen P, Herijgers N, Netscher M, Meskers S, van Eck M, et al. (1997) Particle size determines the specificity of apolipoprotein E-containing triglyceride-rich emulsions for the LDL receptor versus hepatic remnant clearance in vivo. J Lipid Res 38: 1070-1084.

[9] Rothblat GH, Phillips MC (2010) High-density lipoprotein heterogeneity and function in reverse cholesterol transport. Curr Opin Lipidol 21: 229-238.
