# Supplementary material for: A Computational Model for the Analysis of Lipoprotein Distributions in the Mouse: Translating FPLC Profiles to Lipoprotein Metabolism
Source: PLoS Comput Biol. 2014 May 1;10(5):e1003579. doi: 10.1371/journal.pcbi.1003579 (PMC4006703; doi:10.1371/journal.pcbi.1003579)
Supplement: Text S7 — LXR model parametrisation. Parameters of the extended LXR models. (PDF) [file pcbi.1003579.s007.pdf]

Supporting information – Text S7: LXR model parametrisation

A computational model for the analysis of lipoprotein distributions in the mouse: Translating FPLC profiles to lipoprotein metabolism

F. L. P. Sips, C. A. Tiemann, M. H. Oosterveer, A. K. Groen, P. A. J. Hilbers, N. A. W. van Riel

Table 1 – Lipoprotein metabolism model extension parameter values and transformations

Un-transformed parameters				
Parameter		E_1	E_2	E_3
(HDL)				
$scale_A$	$(\frac{\mu mol}{h \cdot kg})$	6.2168	6.0052E-03	2.2733E-03
$ratio_{A,1}$	–	1.0000	1.0000	0.96874
c_{chol}	$(\frac{kg}{\mu mol \cdot nm})$	75.307	0.068661	0.014295
c_{selA}	$(\frac{1}{h \cdot nm})$	3.4277	5.9194E-04	1.7073E-04
c_{lip}	(h^{-1})	42.363	6.5677	13.666
n	–	23.538	6.0797	5.8214
c_{trig}	$(\frac{kg}{\mu mol})$	13.443	0.019128	6.5196E-03
$c_{uptakeA}$	$(\frac{nm}{h})$	265.27	0.16992	0.098619
(VLDL)				
s_{min}	(nm^2)	6.7933E+03	6.3067E+03	6.4787E+03
c_{LPL}	$(\frac{1}{h \cdot nm^2})$	0.16577	0.13926	0.19584
$c_{uptakeB}$	(h^{-1})	0.18361	0.20048	0.31215
c_{selB}	$(\frac{1}{h \cdot nm})$	0.095955	4.2447E-03	0.017805
μ_{upt}	(nm^2)	3.7868E+03	3.7430E+03	3.7970E+03
σ_{upt}	(nm^2)	625.17	562.92	385.65
A_{upt}	$(\frac{nm}{h})$	1.0903E+04	9.2654E+03	1.4960E+04
D	(nm)	69.7391	69.7391	56.0688
$scale_B$	$(\frac{\mu mol}{h \cdot kg})$			$4.0413 * 10^{-3}$

Un-transformed parameters						
Parameter		E_1	E_2	E_3	*L*	*U*
E1						
c_{E1}	$(\frac{1}{h \cdot nm^2})$	83.373	-	-	4 E-3	2.5 E6
$s_{min,2}$	(nm^2)	450.08	-	-	0	$S_{max}/2$
E2						
$A_{upt,E2}$	$(\frac{nm}{h})$	-	3.3981	-	1.6 E-3	$S_{max}/2 * 10^6$
$\mu_{up,E2}$	(nm^2)	-	538.61	-	0	S_{max}
$\sigma_{upt,E2}$	(nm^2)	-	92.313	-	0	$S_{max} / 2$
E3						
$scale_{E3}$	$(\frac{\mu mol}{h \cdot kg})$	-	-	5.6595E-05	7.86 E-4	7.86 E8
μ_{E3}	(# *particles*)	-	-	29.318	15	40
σ_{E3}	(# *particles*)	-	-	2.4696	0	5

	Transformed parameters				
Parameter		*T*	*E1,t*	*E2,t*	*E3,t*
	(HDL)				
$scale_A$	$(\ln(\frac{\mu mol}{h \cdot kg}))$	log	1.8273	-5.1151	-6.0865
$ratio_{A,1}$	$(-)$	lin	1.0000	1.0000	0.96874
c_{chol}	$(\ln(\frac{kg}{\mu mol \cdot nm})$	log	4.3216	-2.6786	-4.2479
c_{selA}	$(\ln(\frac{1}{h \cdot nm}))$	log	1.2319	-7.4321	-8.6754
c_{lip}	$(\ln(h^{-1}))$	log	3.7463	1.8822	2.6149
n	$(\ln(-))$	log	3.1586	1.8050	1.7615
c_{trig}	$(\ln(\frac{kg}{\mu mol}))$	log	2.5984	-3.9566	-5.0329
$c_{uptakeA}$	$(\ln(\frac{nm}{h}))$	log	5.5808	-1.7724	-2.3165
	(VLDL)				
s_{min}	$(-)$	lin	0.16814	0.15610	0.16036
c_{LPL}	$(\ln(\frac{1}{h \cdot nm^2}))$	log	-1.7971	-1.9714	-1.6305
$c_{uptakeB}$	$(\ln(h^{-1}))$	log	-1.6949	-1.6070	-1.1643
c_{selB}	$(\ln(\frac{1}{h \cdot nm}))$	log	-2.3439	-5.4621	-4.0283
μ_{upt}	$(-)$	lin	0.093728	0.092643	0.093980
σ_{upt}	$(-)$	lin	0.030947	0.027866	0.019090
A_{upt}	$(\ln(\frac{nm}{h}))$	log	9.2968	9.1340	9.6131

	Transformed parameters				
Parameter		*T*	*E1,t*	*E2,t*	*E3,t*
E1					
c_{E1}	$(\ln(\frac{1}{h \cdot nm^2}))$	log	4.4233		-
$s_{min,2}$	$(-)$	lin	0.3647		-
E2					
$A_{upt,E2}$	$(\ln(\frac{nm}{h}))$	log		1.2232	-
$\mu_{up,E2}$	$(-)$	lin	-	0.21824	-
$\sigma_{upt,E2}$	$(-)$	lin	-	0.074810	-
E3					
$scale_{E3}$	$(\ln(\frac{\mu mol}{h \cdot kg}))$	log	-	-	-9.7796
μ_{E3}	$(-)$	lin	-	-	0.73294
σ_{E3}	$(-)$	lin	-	-	0.49391
