# Supplementary material for: A Computational Model for the Analysis of Lipoprotein Distributions in the Mouse: Translating FPLC Profiles to Lipoprotein Metabolism
Source: PLoS Comput Biol. 2014 May 1;10(5):e1003579. doi: 10.1371/journal.pcbi.1003579 (PMC4006703; doi:10.1371/journal.pcbi.1003579)
Supplement: Text S8 — Supplemental analyses of the LXR model. Additional visualisations of the parameters and fluxes of the LXR models. Simulations of a knock-out in the LXR activated models. (PDF) [file pcbi.1003579.s008.pdf]

Supporting information – Text S8: Supplemental analyses of the LXR model

A computational model for the analysis of lipoprotein distributions in the mouse: Translating FPLC profiles to lipoprotein metabolism

F. L. P. Sips, C. A. Tiemann, M. H. Oosterveer, A. K. Groen, P. A. J. Hilbers, N. A. W. van Riel

A. Time-dependent model changes following LXR activation

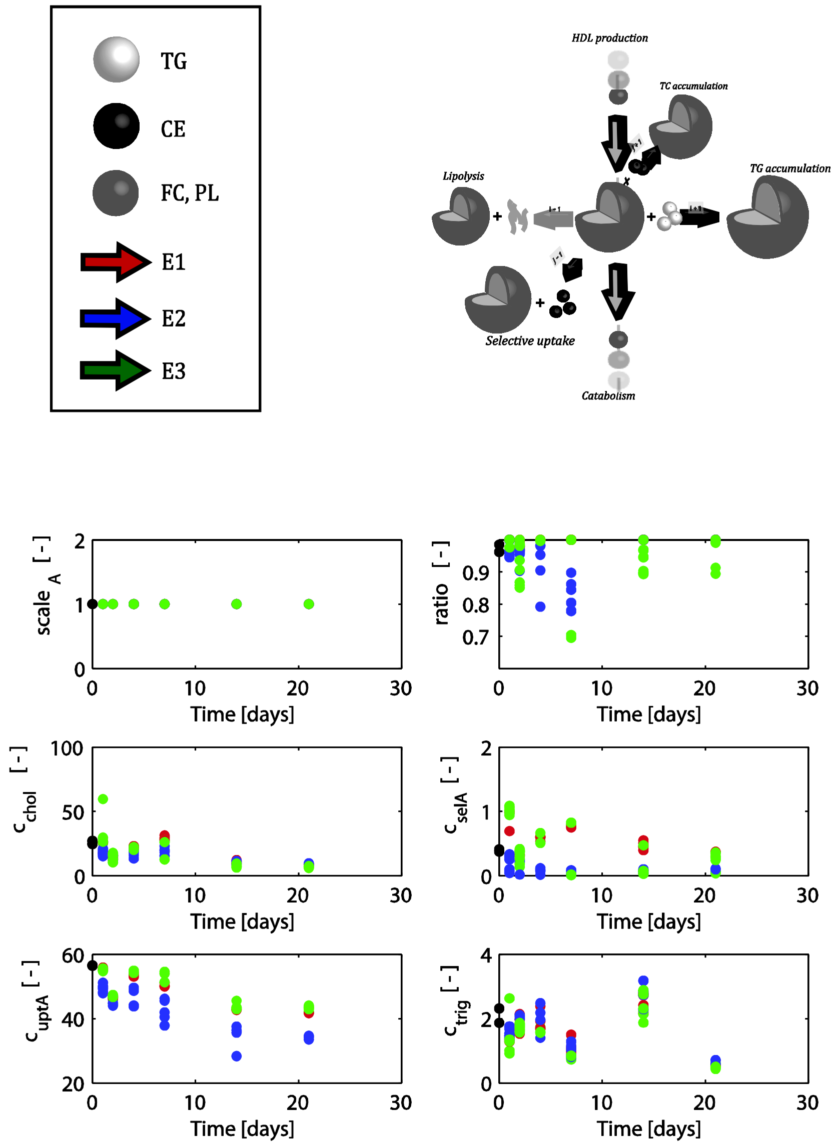

Figure S1: Overview of extended sub-model HDL results.

Legend (top, left) and the development of core model parameter values over time (bottom) as found in the HDL sub-model. The plots include all parameter sets that describe the profiles quantitatively and qualitatively (Text S6). Parameters in the E1 extended model are plotted in red, parameters in the E2 extended model are plotted in blue and parameters in the E3 extended model are plotted in green. In this figure, all assumed logarithmically distributed parameters are displayed relative to (i.e., divided by) parameter $scale_A$ while the geometrical, assumed linearly distributed parameters are displayed in the transformed format (i.e. divided by the upper bound).

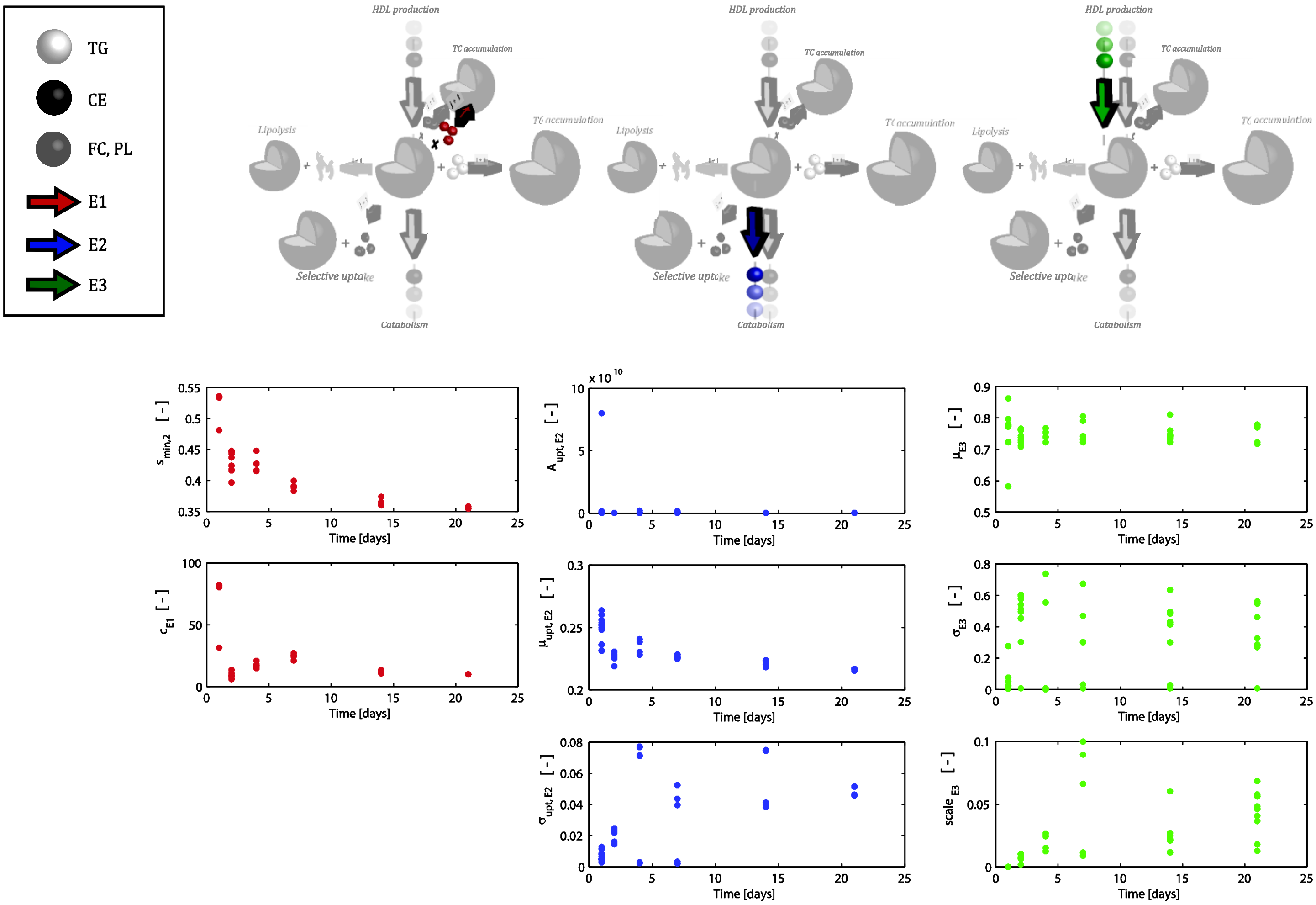

Figure 2: Overview of extended sub-model HDL results (2).

Legend (top) and extended model parameters specific to E1 (bottom, left), E2 (bottom, middle) and E3 (bottom, right). The plots include all parameter sets that describe the profiles quantitatively and qualitatively (Text S6). Parameters in the E1 extended model are plotted in red, parameters in the E2 extended model are plotted in blue and parameters in the E3 extended model are plotted in green. In this figure, all assumed logarithmically distributed parameters are displayed relative to (i.e., divided by) parameter $scale_A$ while the geometrical, assumed linearly distributed parameters are displayed in the transformed format (i.e. divided by the upper bound).

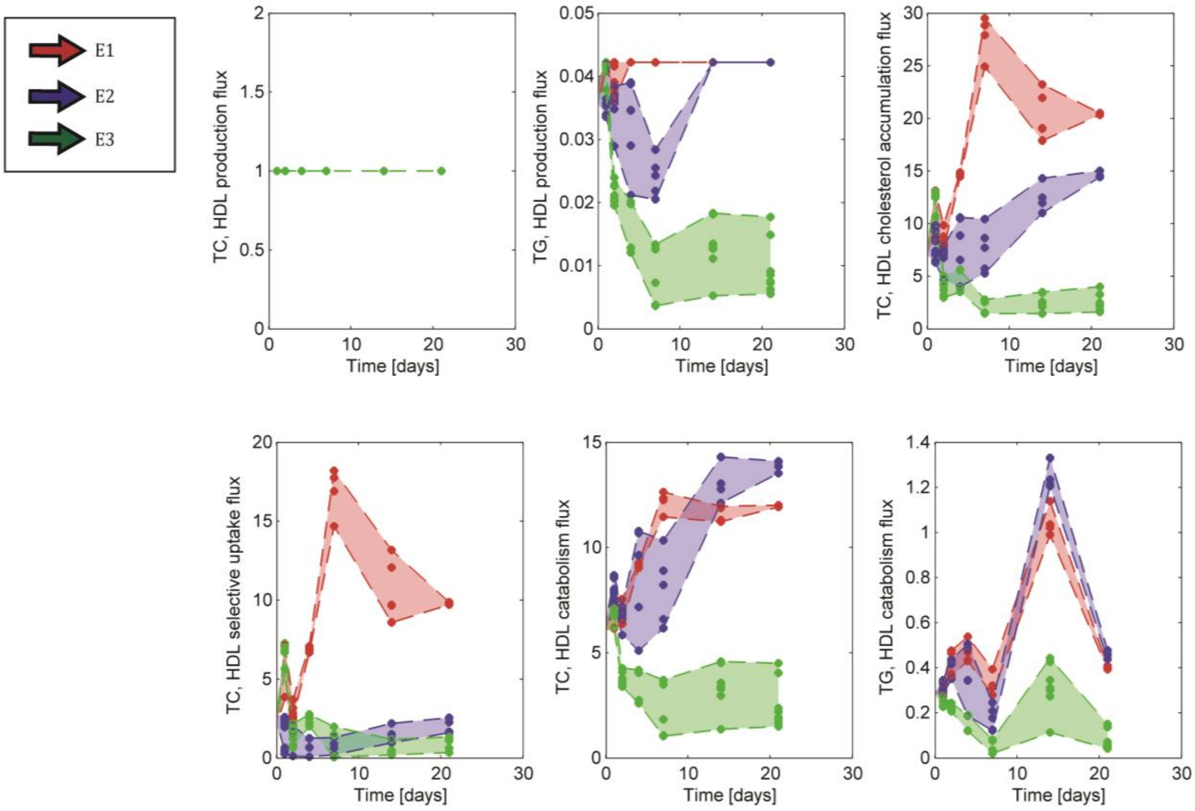

Figure 3: Overview of extended sub-model HDL results (3). Progression of fluxes in the extended models. Note that the flux through the extended pathway now contains the flux through both the original and new equations. Fluxes are scaled with the HDL production flux (as can be clearly seen in the HDL C production flux panel). Again, red is for E1, blue is for E2 and green is for E3.

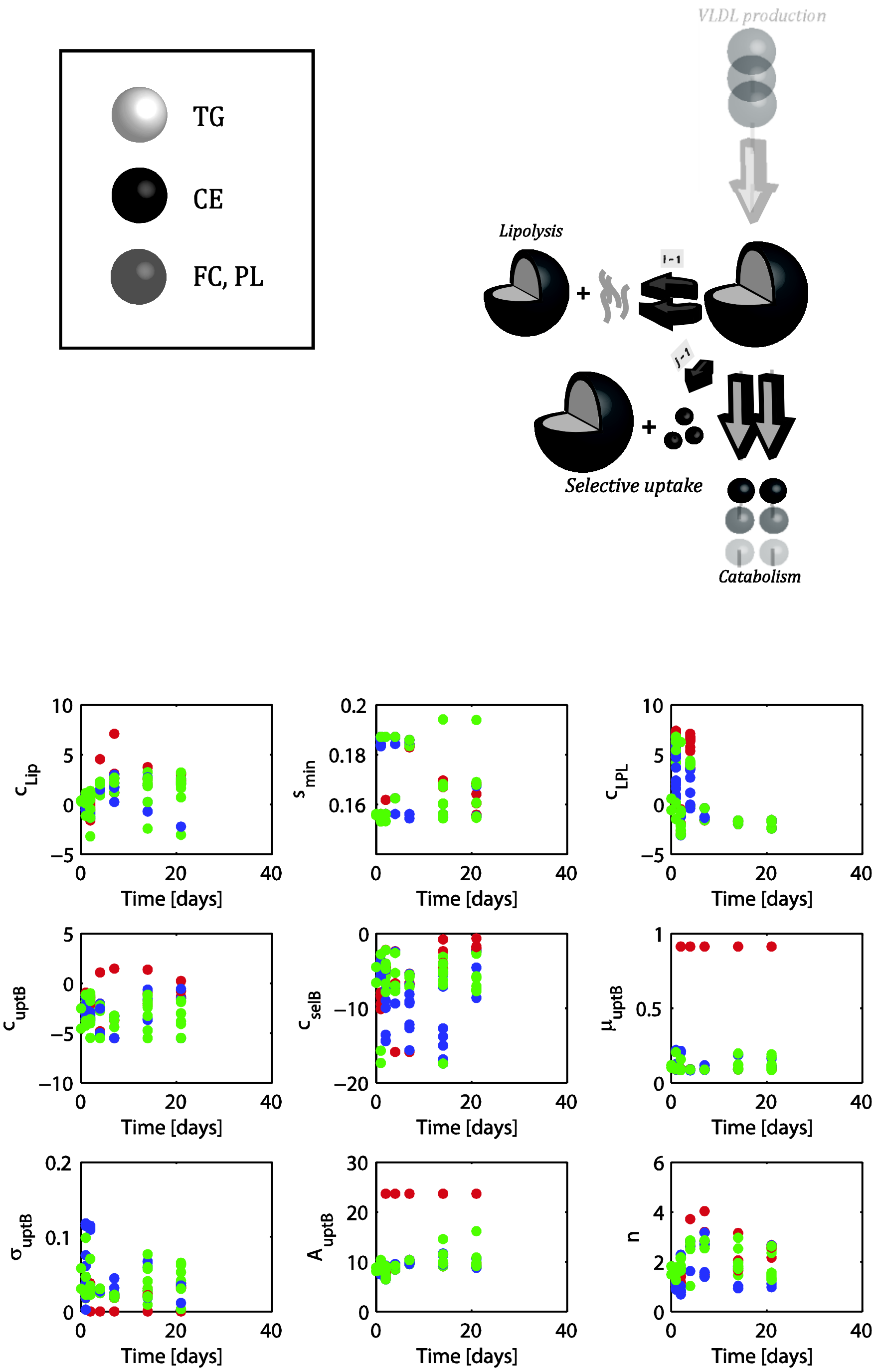

Figure 4 - Overview of VLDL sub-model results for LXR activation.
Complementary to Figure S1, this figure shows the course of VLDL sub-model parameters during LXR activation. Legend (top) and VLDL sub-model parameters (bottom), which unlike HDL sub-model parameter do not need scaling. Assumed logarithmically distributed parameters are plotted as the natural logarithm, while assumed linearly distributed parameters are plotted relative to the upper bound

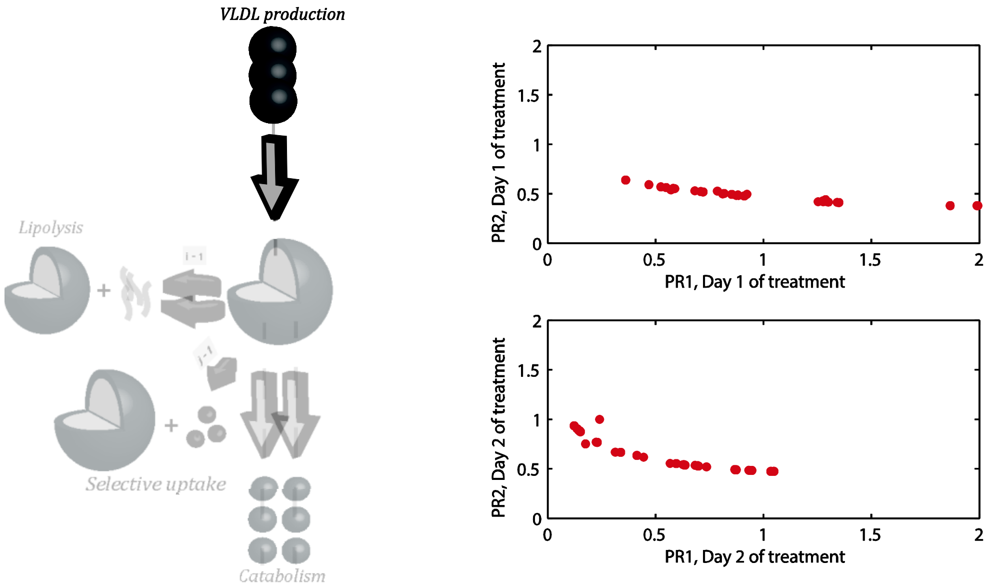

Figure 5 - Overview of VLDL sub-model results for LXR activation (2)
On the right, the additional production parameters are plotted for Day 1 (right, top) and Day 2 (right, bottom) of treatment. Here, the true value of PR1 [-] is plotted against the value of PR2, linearly scaled such that a value of 1 is a nascent VLDL size of the maximal size in the model.

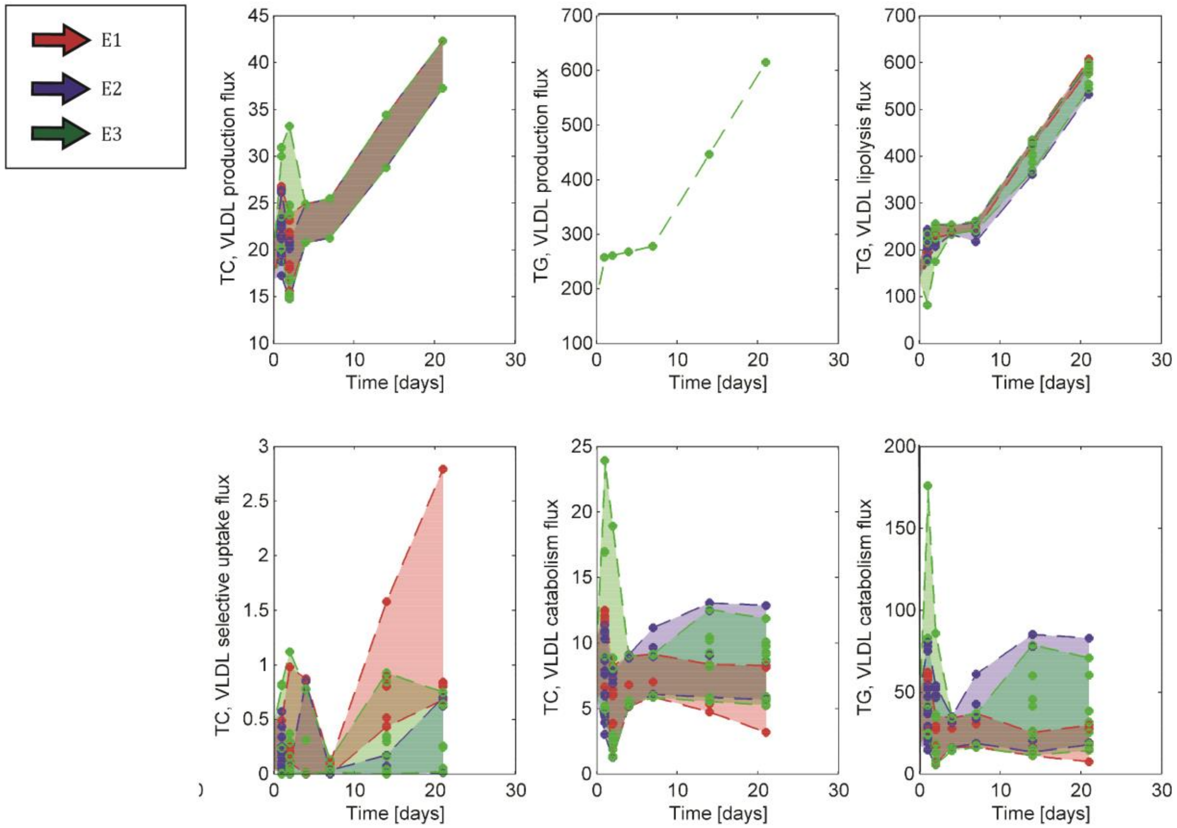

Figure 6 - Overview of VLDL sub-model results for LXR activation (3)

VLDL sub-model fluxes, in µmol/kg/hour. VLDL-TG production is fixed, as can be seen in the second panel.

B. LXR activation in *in silico* knock-out phenotypes

In this text, the combination of the two model perturbations as described in this study is shown. The simulations show the profiles the model predicts for a 14 days treated transgenic mouse (E1, E2 and E3; SR-B1 knock-out and PLTP knock-out). First, the wild-type model parameters were perturbed in order to simulate an untreated genetically modified mouse. The resultant profiles for both the SR-B1 knock-out mouse and the PLTP knock-out mouse are shown in the main text for one parameter set, and in Text S5 for X2. The profile of the SR-B1 deficient mouse displays an increase in HDL particle size and an increase in HDL cholesterol concentration.

***In silico* profiles of SR-B1 knock-out mice treated with T0901317**

Having created qualitatively accurate knock-out mice simulations from the wild-type mouse phenotype, we applied the same technique to simulate LXR activation of genetically modified mice. In [2], FPLC profiles of SR-B1 knock-out mice treated with T0901317 for 14 days were measured. Because the dosage and duration of treatment, as well as additional (laboratory) conditions are similar between these mice and the data used for parameterisation [2] we can apply the same parameter perturbation as in the main text to the extended models to simulate the LXR activated SR-B1 mouse. The results of this perturbation is seen in Figure 7A.

Note that the profiles of several of the knock-out simulations exit the plot at the top as they are accumulating in large numbers in fraction 15. Because the model has a closed boundary, in the case the HDL lipoproteins are modelled to grow at unphysiological rates the lipoproteins can accumulate at the upper boundary. In the combination of LXR activation and SR-B1 knock-out, this unimpeded growth can be seen in both E1 and E2 and accumulation of large amounts of HDL can be seen. As the experimental data suggests that the lipoproteins should be contained within the model boundaries ([2]), we do not show a knock-out activated plot with extended boundaries. However, the E1 model especially shows accumulation against the boundary even if the model is extended far beyond physiological boundaries. However, we note that a good description of the data may require and be reasonable with re-optimization of E1.

***In silico* profiles of PLTP knock-out mice treated with T0901317**

Cao et al, 2002 [1], performed a similar experiment on a PLTP knock-out phenotype. While the PLTP

knock-out mouse was treated with a different/higher dose of T0901317 than the parameterised LXR activated mouse for a duration of 1 week, the response should be characterised by the preservation of the two distinct peaks, with a decrease in the height of both. The results are shown in Figure 7 B.

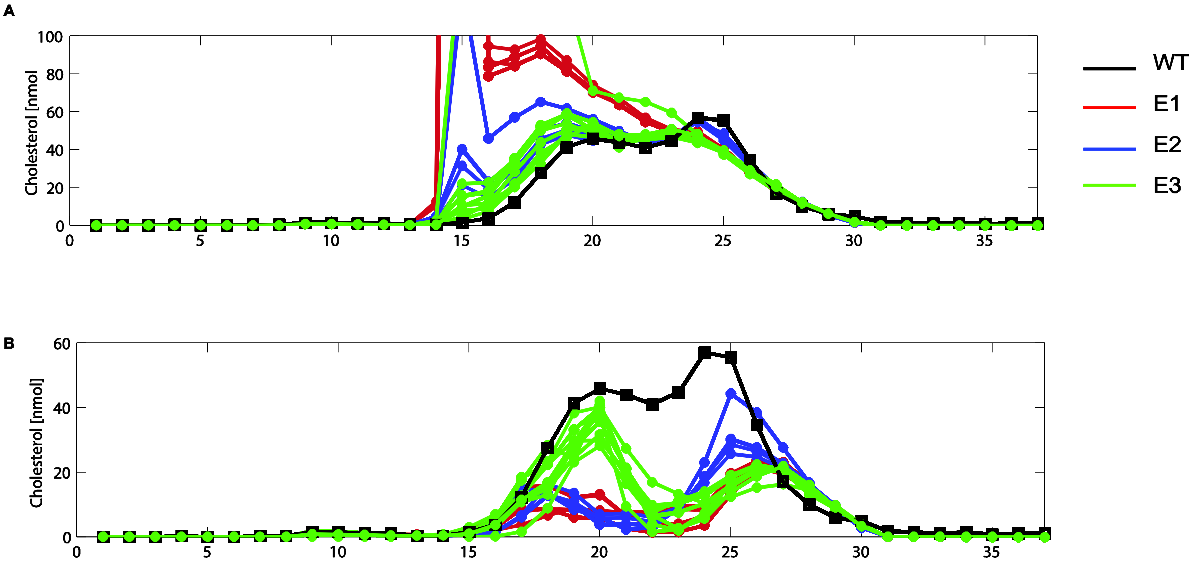

Figure 7 - *In silico* TC profiles of 14-day LXR activated SR-B1 and PLTP knock-out mice

A. SR-B1 knock-out (10^{-10} % of original parameter value), 14 days of T0901317 treatment. The experimental data of 14-days LXR activation is shown for comparison. Red = E1, Blue = E2, Green = E3. **B.** PLTP knock-out (30 % of original parameter value), 14 days of T0901317 treatment. Red = E1, Blue = E2, Green = E3. The experimental data of 14-days LXR activation is shown for comparison.

References

[1] Cao G, Beyer TP, Yang XP, Schmidt RJ, Zhang Y, et al. (2002) Phospholipid transfer protein is regulated by liver X receptors in vivo. J Biol Chem 277: 39561-39565.

[2] Grefhorst A, Oosterveer MH, Brufau G, Boesjes M, Kuipers F, et al. (2012) Pharmacological LXR activation reduces presence of SR-B1 in liver membranes contributing to LXR-mediated induction of HDL-cholesterol. Atherosclerosis 222: 382 - 389.

[3] Jiang XC, Beyer TP, Li Z, Liu J, Quan W, et al. (2003) Enlargement of high density lipoprotein in mice via liver X receptor activation requires apolipoprotein E and is abolished by cholesteryl ester transfer protein expression. J Biol Chem 278: 49072-49078.
