# Supplementary material for: A Computational Model for the Analysis of Lipoprotein Distributions in the Mouse: Translating FPLC Profiles to Lipoprotein Metabolism
Source: PLoS Comput Biol. 2014 May 1;10(5):e1003579. doi: 10.1371/journal.pcbi.1003579 (PMC4006703; doi:10.1371/journal.pcbi.1003579)
Supplement: Text S9 — Flux FPLC profiles. Flux FPLC profiles of wild-type, knock-out, and 14 days LXR activated models. (PDF) [file pcbi.1003579.s009.pdf]

Supporting information – Text S9: Flux FPLC profiles

A computational model for the analysis of lipoprotein distributions in the mouse: Translating FPLC profiles to lipoprotein metabolism

F. L. P. Sips, C. A. Tiemann, M. H. Oosterveer, A. K. Groen, P. A. J. Hilbers, N. A. W. van Riel

In this supplemental text, we provide illustration of the relations between the processes in the various phenotypes. As the interpretation of the grid itself is difficult due to the non-linearity, we have chosen to illustrate the distribution of fluxes over the FPLC profile.

The calculation of such a flux FPLC is explained in Text S3.

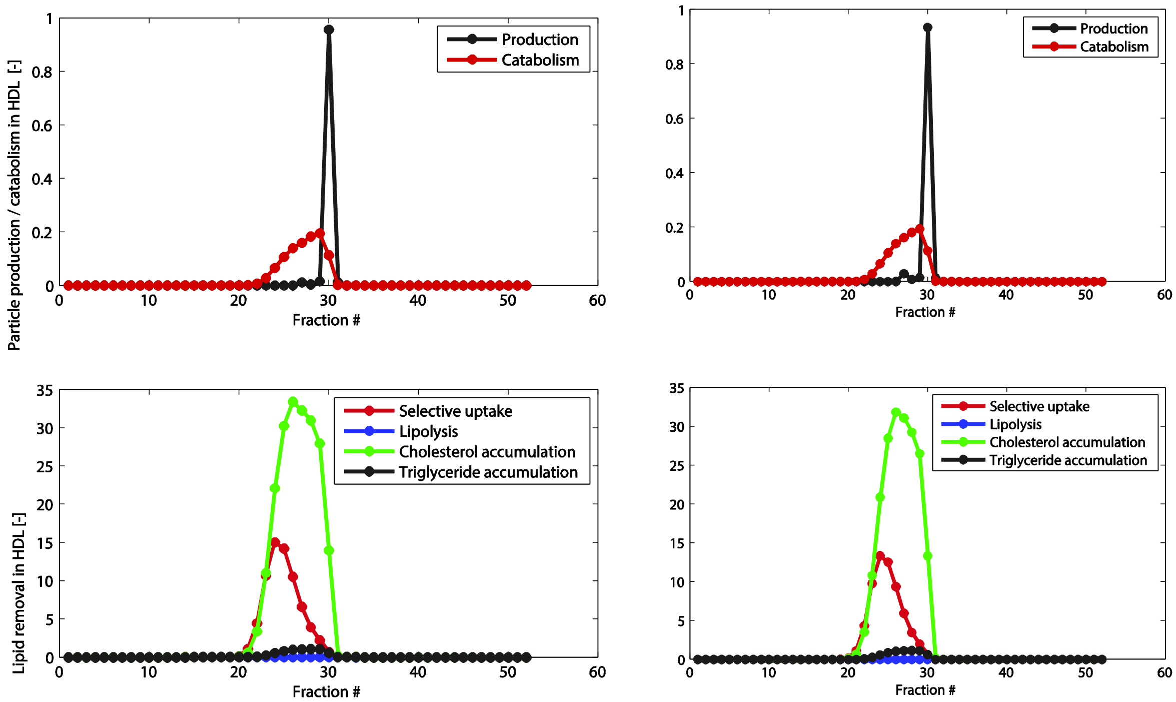

Figure 1: Distribution of fluxes over the FPLC profile in wild-type *in silico* HDL metabolism. Left: parameter set X1, Right: parameter set X2

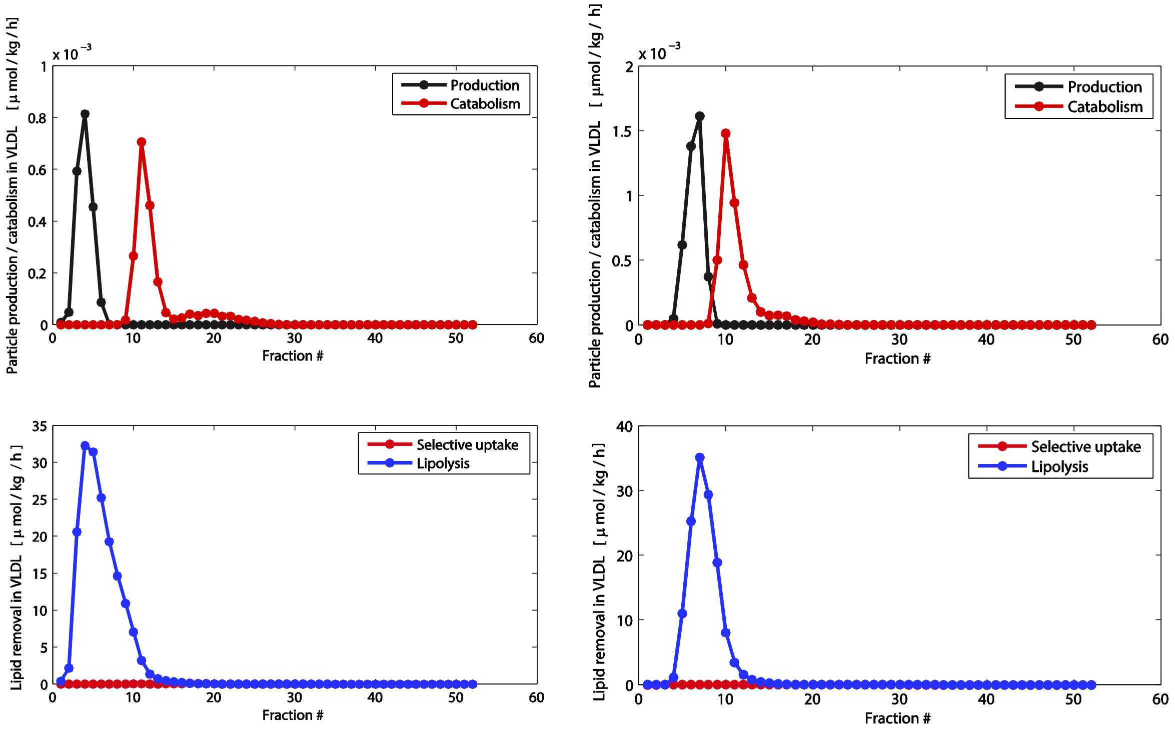

Figure 2: Distribution of fluxes over the FPLC profile in wild-type *in silico* VLDL metabolism.
Left: parameter set X1, Right: parameter set X2

From comparison of the wild-type VLDL production curves in Figure 2, the difference in nascent VLDL diameter is apparent.

The SR-B1 profiles show a clear absence of selective uptake (Figure 3, left). The reduction of PLTP deficient mouse cholesterol accumulation is less apparent, however a comparison of the relative scale of the figures reveals that cholesterol accumulation is much lower than in the wild-type mouse (Figure 3). In the LDLr knock-out mouse, the reduction of catabolism (and subsequent accumulation of LDL) is clearly visible.

In Figures 4-6, the acceptable parameter sets at t=14 days of LXR activation are plotted for E1 (Figure 4), E2 (Figure 5) and E3 (Figure 6).

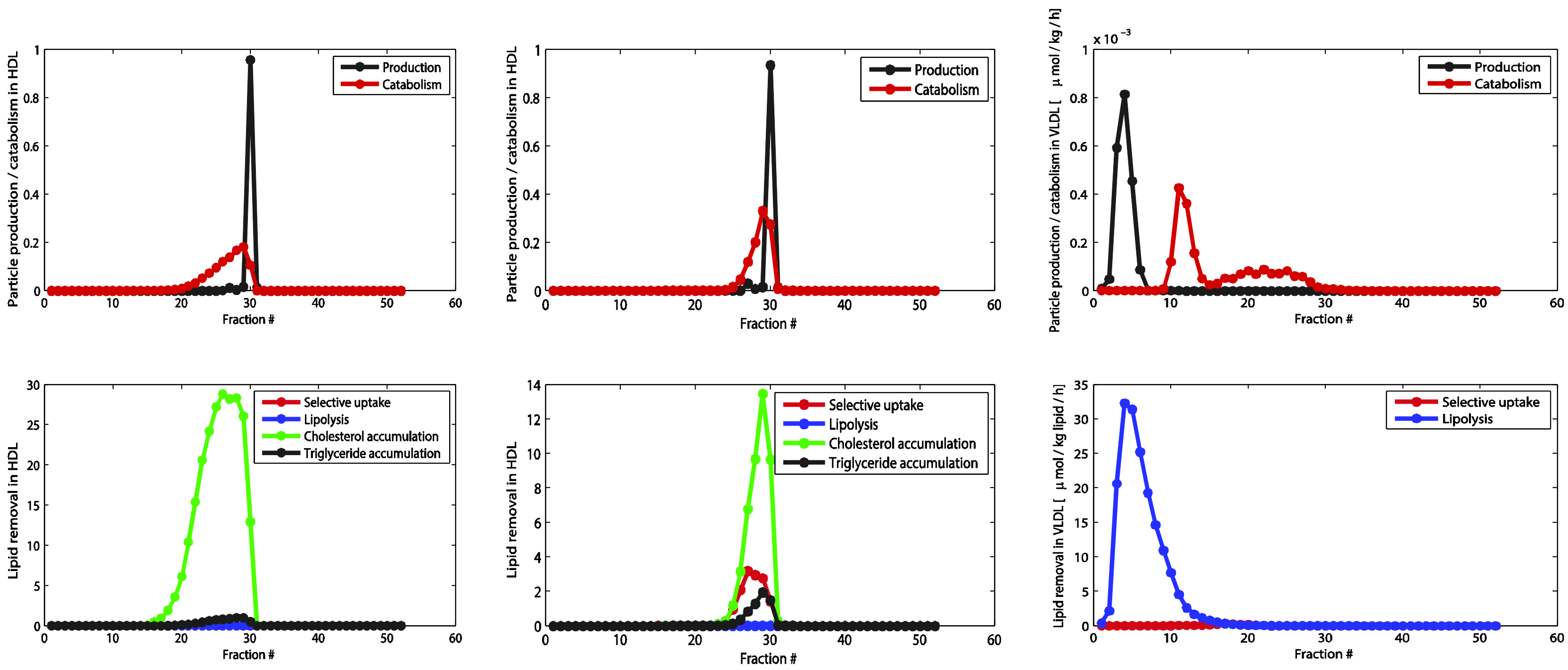

Figure 3: Distribution of fluxes in the knock-out phenotypes. Left: HDL metabolism in the *in silico* SR-B1 knock-out mouse. The figure was generated with parameter set X1, and a value of 10^{-10} times the original parameter c_{selB}. Middle: HDL metabolism in the *in silico* PLTP knock-out mouse. The figure was generated with parameter set X1 and a value for c_{chol} of 30 % of the original value. Right: VLDL metabolism in the *in silico* LDLr-KO mouse. The figure was generated with X1 and a value of 40 % of the original value of both Apo B uptake parameters.

The following figures depict the distribution of fluxes over the FPLC profile in the LXR-activated case – in particular, here we only depict the 14 days' time point.

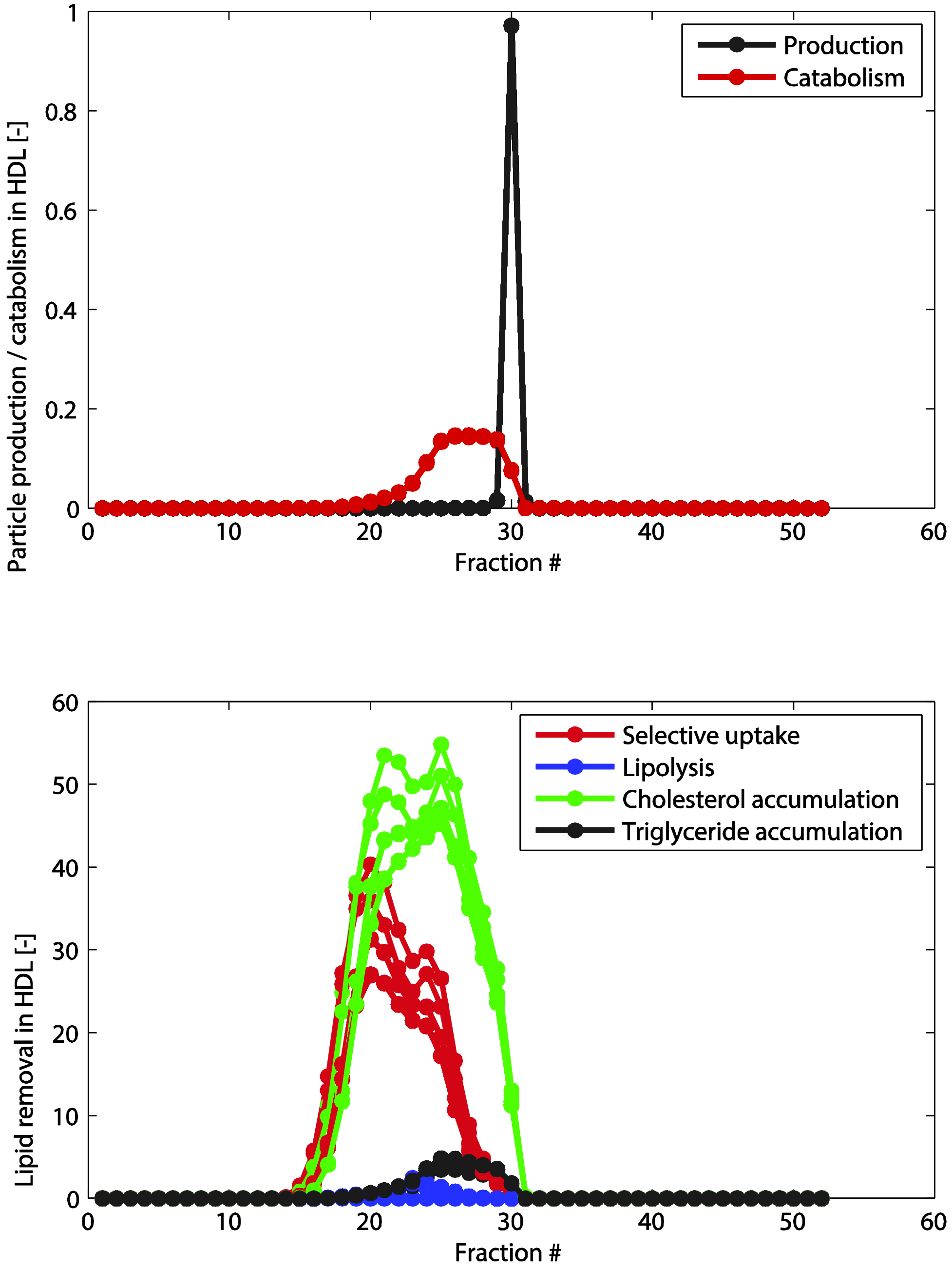

Figure 4: Distribution of fluxes in the HDL sub-model in the *in silico* 14 days LXR activated case, assuming E1. All acceptable parameter sets are included. HDL Flux FPLCs are scaled to total HDL production, in # of particles.

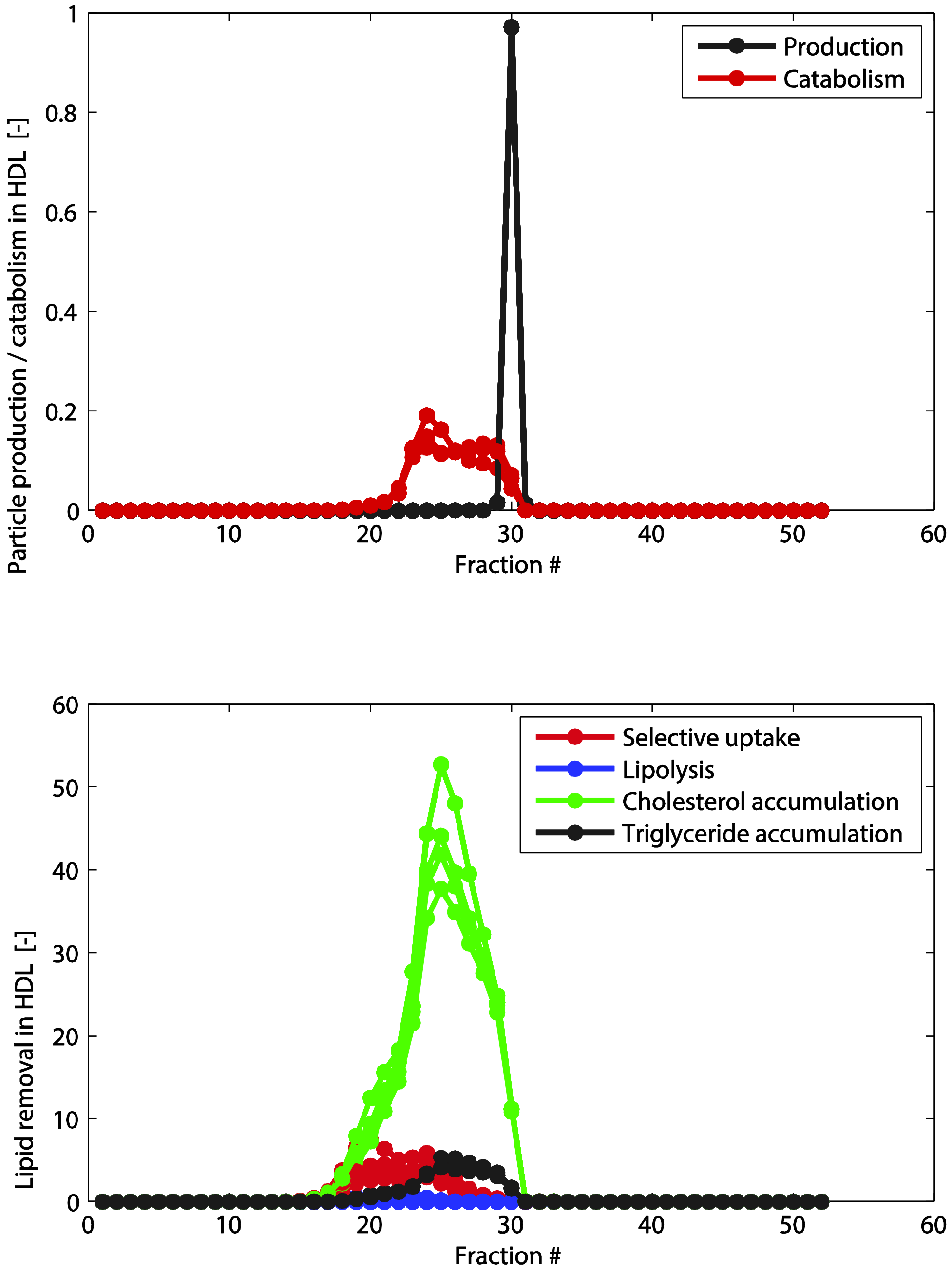

Figure 5: Distribution of fluxes in HDL sub-model the *in silico* 14 days LXR activated case, assuming E2. All acceptable parameter sets are included. HDL Flux FPLCs are scaled to total HDL production, in # of particles.

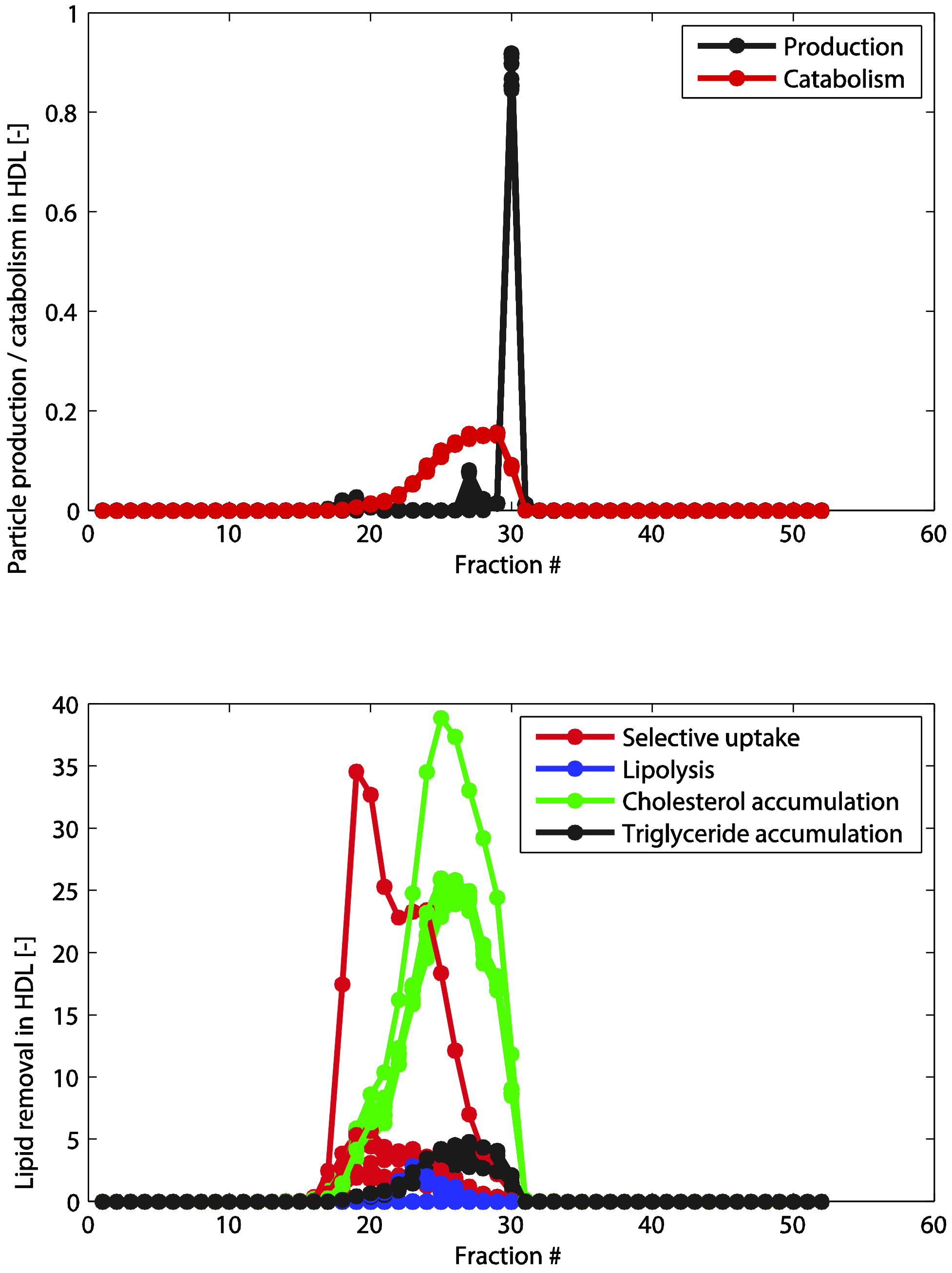

Figure 6: Distribution of fluxes in HDL sub-model the *in silico* 14 days LXR activated case, assuming E3. All acceptable parameter sets are included. HDL Flux FPLCs are scaled to total HDL production, in # of particles.
